# Promoter Variation of the Key Apple Fruit Texture Related Gene *MdPG1* and the Upstream Regulation Analysis

**DOI:** 10.3390/plants12071452

**Published:** 2023-03-26

**Authors:** Mengmeng Wu, Zhengrong Luo, Shangyin Cao

**Affiliations:** 1 Zhengzhou Fruit Research Institute, Chinese Academy of Agriculture Sciences, Zhengzhou 450009, China; 2 National Key Laboratory for Germplasm Innovation & Utilization of Horticultural Crops, Huazhong Agricultural University, Wuhan 430070, China

**Keywords:** *MdPG1*, *Malus* × *domestica*, MdCBF2, *lncRNA_PG1_*, *cis*-regulation

## Abstract

*MdPG1* encoding polygalacturonase in apple (*Malus* × *domestica*) is a key gene associated with fruit firmness and texture variations among apple cultivars. However, the causative variants of *MdPG1* are still not known. In this study, we identified a SNP^A/C^ variant within an ERF-binding element located in the promoter region of *MdPG1*. The promoter containing the ERF-binding element with SNP^A^, rather than the SNP^C^, could be strongly bound and activated by MdCBF2, a member of the AP2/ERF transcription factor family, as determined by yeast-one-hybrid and dual-luciferase reporter assays. We also demonstrated that the presence of a novel long non-coding RNA, *lncRNA_PG1_*, in the promoter of *MdPG1* was a causative variant. *lncRNA_PG1_* was specifically expressed in fruit tissues postharvest. *lncRNA_PG1_* could reduce promoter activity when it was fused to the promoter of *MdPG1* and a tobacco gene encoding Mg-chelatase H subunit (*NtCHLH*) in transgenic tobacco cells but could not reduce promoter activity when it was supplied in a separate gene construct, indicating a *cis*-regulatory effect. Our results provide new insights into genetic regulation of *MdPG1* allele expression and are also useful for the development of elite apple cultivars.

## 1. Introduction

Fruit softening is a complex process of cell wall disassembly that is induced by ethylene in climacteric fruits. The biosynthesis of ethylene, a gaseous plant hormone, starts just prior to the initiation of ripening in the climacteric fruit, such as apple, and produces an ethylene burst as ripening progresses [1,2,3]. After the ethylene burst, the fruit becomes soft and loses its storage ability and then its commercial value.

Ethylene biosynthesis is sequentially catalyzed by two enzymes, 1-aminocyclopropane-1-carboxylate synthase (ACS) and 1-aminocyclopropane-1-carboxylate oxidase (ACO). In apple, *MdACS1* and *MdACO1* are highly expressed during fruit ripening and responsible for the production of ethylene. Genetic variants that affect gene expression level have been identified in the two genes. For example, a SINE type transposable element in the *MdACS1* promoter region is known to inhibit *MdACS1* expression [4]. Similarly, a deletion of a 62 bp fragment in the third intron of *MdACO1* reduces *MdACO1* expression [5]. Apple cultivars that are homozygous for these *MdACS1* and *MdACO1* variants produce little ethylene during fruit ripening and have a prolonged storage and shelf life [5,6,7]. Ethylene signaling relies on a linear signal transduction pathway. The ethylene response factors (ERFs) that act downstream are the last component of the ethylene signal transduction pathway to regulate the ethylene-responsive gene’s expression. They function as transcriptional modulators by binding to the promoter elements such as GCC box or DRE/CRT motif in many plant species [8,9,10,11]. In apple, EIL and COLD BINDING FACTOR (CBF) type ERFs could transactivate the promoter of *MdPG1* (polygalacturonase 1) in response to cold- and ethylene-induced fruit ripening and promote fruit softening [12]. MdERF2 directly suppresses the *MdACS1* expression by binding to a DRE motif of the *MdACS1* promoter [13], and MdERF4 affects the ethylene signaling pathway and fruit firmness by directly binding to the promoter of *ERF3* [14].

Ethylene induces the expression of genes encoding the cell wall degradation enzymes that causes fruit softening. The cell wall degradation enzymes include polygalacturonase (PG), xyloglucan endotransglycosylase (XET), pectate lyase (PL), α-arabinofuranosidase (α-AFase), galactanase (β-Gase) and pectin methylesterase (PME) [15,16,17,18]. In apple, *MdPG1* is co-located with a fruit firmness QTL on chromosome 10 [19]. The transcription level of *MdPG1* is positively correlated with the rate of fruit softening. Overexpression of *MdPG1* in apple trees disrupts leaf cell organization and stomata structure, thus promoting water loss through stomatal transpiration [20]. Conversely, suppression of *MdPG1* expression reduces pectin depolymerization and water loss from postharvest fruit, changes the structure of hypodermal cell layers, alters cell fracture properties, and leads to firmer apple fruit [21]. Several molecular markers for different *MdPG1* alleles have been developed for the selection of cultivars with improved fruit texture [22,23,24,25]. However, the causative genetic variants in these alleles are largely unknown.

Long non-coding RNAs (lncRNAs) have been shown to affect gene expression through a wide range of mechanisms and are considered as important regulators in many essential biological processes [26,27,28,29,30,31,32,33,34,35,36,37,38,39,40,41,42,43,44,45,46,47,48]. Several studies have demonstrated that lncRNAs may act as regulators of fruit ripening. Silencing two tomato intergenic lncRNAs (*lncRNA1459* and *lncRNA1840*) delays fruit ripening [49,50]. Silencing two sea buckthorn lncRNAs (*LNC1* and *LNC2*) reduces anthocyanin biosynthesis during fruit ripening [51]. Through genome and transcriptome analyses, lncRNAs have been predicted to suppress photosynthesis and cell wall biogenesis in strawberry [52], to promote the effect of abscisic acid on ethylene biosynthesis and fruit softening in kiwifruit [53], and to regulate auxin signal transduction, sucrose biosynthesis, and metabolism during fruit development and ripening in *Cucumis melo* [54]. However, the potential functions of these lncRNAs are waiting to be analyzed.

Apple lncRNAs have been identified from different tissues including young fruits, shoot tips, stem phloem, root tips, and peel [55]. However, functional study of apple lncRNAs is limited to anthocyanin biosynthesis. Two apple lncRNAs (MLNC3.2 and MLNC4.6) are shown to function as endogenous target mimics (eTMs) for miR156a, thus preventing miR156a from cleaving the mRNA of *SQUAMOSA PROMOTER BINDING PROTEIN-LIKE 33* (*SPL33*) and *SPL2-like* genes during light-induced anthocyanin biosynthesis [47]. In addition, *MdLNC499* is shown to bridge the function of MdWRKY1 and MdERF109 to regulate early-stage light-induced anthocyanin accumulation [48].

This study was focused on *MdPG1* due to its importance in apple fruit cell well degradation. By using molecular biology approaches, we identified a causative SNP within an ERF-binding element located in the *MdPG1* gene promoter region and showed that this SNP affected *MdPG1* expression through altering the binding of ERF proteins to the *MdPG1* promoter. In addition, a novel lncRNA, *lncRNA_PG1_*, was shown to be located in the *MdPG1* promotor region and inhibited the expression of *MdPG1* by *cis*-action.

## 2. Results

### 2.1. Genetic Variants in the Promoter of MdPG1

**Figure 1:**
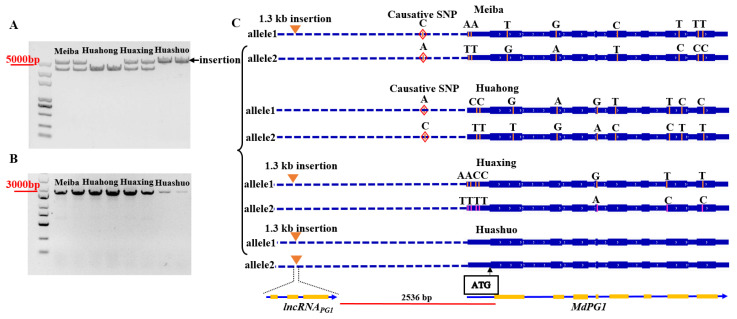
Genetic variants of *MdPG1* alleles in four apple cultivars. (**A**,**B**) DNA fragments covering the promoter (**A**) and coding regions (**B**) in “Meiba”, “Huahong”, “Huaxing”, and “Huashuo”. PCR analysis of the four cultivars detected a larger DNA fragment in three cultivars indicating DNA insertion in the *MdPG1* promoter. The arrow indicates a 1.3 kb insertion in the larger fragment of the *MdPG1* promoter region in “Meiba”, “Huaxing”, and “Huashuo”. (**C**) The SNPs and *lncRNA_PG1_* were phased into allele 1 and allele 2 after genomic PCR fragments covering the promoter and coding regions of the two alleles of *MdPG1* were cloned and fully sequenced. The triangle represents a 1.3 kb insertion (*lncRNA_PG1_*) at 2356 bp upstream of the ATG start codon. The diamond represents an ERF-binding element, and the causative SNP was within the ERF-binding element at 411 bp upstream of the ATG start codon.

To identify the causative genetic variants that may be responsible for *MdPG1* expression variations in different apple cultivars, the DNA sequences covering the coding and promoter regions of *MdPG1* alleles in four apple cultivars (“Meiba”, “Huahong”, “Huaxing” and “Huashuo”) (Table S1) were PCR-amplified. A 4.8 kb and a 3.5 kb DNA promoter fragments in different apple cultivars were obtained. The four apple cultivars could be classified into three insertion genotypes, homozygous showing the 4.8 kb DNA fragment, heterozygous showing both 4.8 kb and 3.5 kb fragments, and without insertion showing the 3.5 kb fragment (Figure 1A). The size of DNA fragments covering the coding regions in the four cultivars was the same (Figure 1B). The PCR fragments of two *MdPG1* alleles in all the cultivars were cloned and fully sequenced (Figure S1). The alignment of the two allele sequences in the “Meiba” and “Huaxing” promoter showed a 1.3 kb insertion in the larger fragment (named as allele 1) compared to the small fragment (named as allele 2), and the insertion was flanked by an 8-bp direct repeat (Figure S1A,E). “Huashuo” was shown to be homozygous for the insertion (Figure S1G). After confirming the presence of an insertion at 2.5 kb upstream from the start codon ATG of *MdPG1* in some apple cultivars, variations were further identified in the 2.5 kb region upstream from the start codon of *MdPG1*. These variations included 3 indels and 34 SNPs in the promoter sequences of *MdPG1* (Figure S1A,C,E). In the same upstream region, three AP2/ERF and six EIN3/EIL *cis*-elements (Figure S1C) were identified using the Plantpan2 database (http://plantpan2.itps.ncku.edu.tw, accessed on 1 June 2020) [56]. One of the SNPs identified above was within an ERF-binding element (AGAGTCGGC*A*/(*C*)A) at 411 bp upstream of the ATG start codon (Figure S1C). Furthermore, two alleles of MdPG1 for differential TF binding were scanned using the CIS-BP database (http://cisbp.ccbr.utoronto.ca/TFTools.php, accessed on 1 March 2023) [2], and the results showed that AP2 was more likely to bind to the sequence (AGTCGGCAA, −418–−410) of allele 1, but not allele 2 (Table S2). This SNP may be a key variation related to *MdPG1* expression. The haploid type containing the insertion and the SNP within the ERF-binding element for all four apple cultivars are shown in Figure 1C.

### 2.2. Differentially Expression of MdPG1 Alleles Is Regulated by MdCBF2

**Figure 2:**
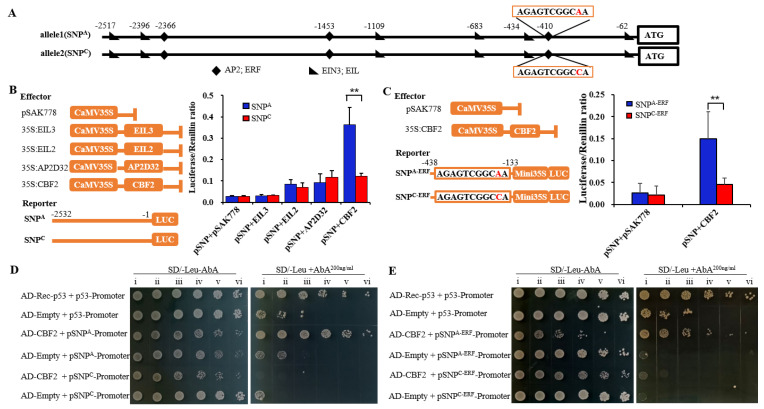
The SNP site in an ERF−binding element is differentially regulated by MdCBF2. (**A**) Cis-acting regulatory elements for AP2/ERF and EIN3/EIL transcription factors were identified in the *MdPG1* promoter of “Huahong” using the Plantpan2.0 (http://plantpan2.itps.ncku.edu.tw, accessed on 1 June 2020). An SNP^A/C^ site in an ERF-binding element was located 411 bp upstream of the start codon. (**B**,**C**) Dual-luciferase assays were carried by co-infiltration of *Nicotiana benthamiana* leaves with effector gene constructs (CaMV35S promoter driving no CDS or is driving the CDS of MdEIL2, MdEIL3, MdCBF2 or MdAP2D32) and reporter gene constructs (2.5 kb with SNP^A^ or SNP^C^, and 0.3 kb plus minimal CaMV 35S with SNP^A^ or SNP^C^ fused to the *LUC* coding sequence). The error bars are standard deviations of the mean from six repeats. Asterisks indicate significant differences by *t*-test: ** *p* < 0.01. (**D**,**E**) Yeast-one-hybrid assay showed that MdCBF2 specifically bound to 2.5 kb (**D**) and 0.3 kb (**E**) promoter sequence of *MdPG1* allele containing the SNP^A^ within the ERF-binding element. Yeast cells co-transformed with the constructs named on the left were cultured on non-selective medium SD/-Leu/-AbA (left panel) and selective medium SD/-Leu/+AbA^200 ng/mL^ (right panel), in a dilution series of 5^0^, 5^−1^,5^−2^, 5^−3^, 5^−4^, and 5^−5^ (i–vi). Rec-p53 and the p53-promoter were used as positive controls. The empty vector and the promoter fragments of *MdPG1* were used as negative controls.

To determine if this SNP within the ERF-binding element affected *MdPG1* expression, “Huahong” was selected as material for analyzing because it contained this key SNP but no structure variations that may also affect gene expression. MdEIL2 and MdCBF2 were shown to *trans*activate the *MdPG1* promoter in the presence of ethylene or under cold conditions in a previous study [12]. We tested MdEIL2, MdEIL3, MdCBF2 and MdAP2D32 to determine whether they differently activate the *MdPG1* promoters of “Huahong” with different nucleotides at the SNP site within the ERF-binding element. MdEIL2 and MdEIL3 are closely related to AtEIL2 (Figure S2A), and MdCBF2 and MdAP2D32 are closely related to AtCBFs (Figure S2B). In dual-luciferase transient expression assays, the luciferase gene was driven by the 2.5 kb promoter of the SNP^A^ or SNP^C^ allele of *MdPG1* (Figure 2A–C). MdEIL3 did not significantly increase the luciferase activity compared to the pSAK778 empty vector control. MdEIL2 and MdAP2D32 slightly enhanced the luciferase activity driven by the 2.5 kb promoter of both *MdPG1* alleles. Interestingly, MdCBF2 significantly enhanced the luciferase activity driven by the promoter of the SNP^A^ allele (Figure 2B). Furthermore, the above experiment was repeated using a fusion promoter of minimal CaMV35S and a short (0.3 kb) *MdPG1* promoter containing the SNP site. MdCBF2 also significantly enhanced the luciferase activity driven by the 0.3 kb promoter sequences of the SNP^A^ allele compared to the SNP^C^ allele (*p* < 0.01) (Figure 2C). These results suggested that the SNP^A^ within an ERF-binding element has a significant effect on the activation of *MdPG1* expression via interaction with MdCBF2 transcription factor.

To further determine whether SNP^A/C^ could alter MdCBF2 binding ability to the ERF-binding element, we performed yeast-one-hybrid experiments. The results showed that the yeast cells transformed with the SNP^A^ allele (2.5 kb or 0.3 kb) grew well under aureobasidin A (200 ng/mL) selection, whereas the yeast cells transformed with the SNP^C^ allele grew poorly under the same selection condition (Figure 2D,E), indicating that MdCBF2 binding was much stronger to the promoter of the SNP^A^ allele than to the promoter of the SNP^C^ allele of *MdPG1*. Taken together, these results suggest that MdCBF2 directly binds to the promoter of the SNP^A^ allele of *MdPG1* to enhance *MdPG1* expression.

### 2.3. Identification and Characterization of an lncRNA in the Promoter of MdPG1

**Figure 3:**
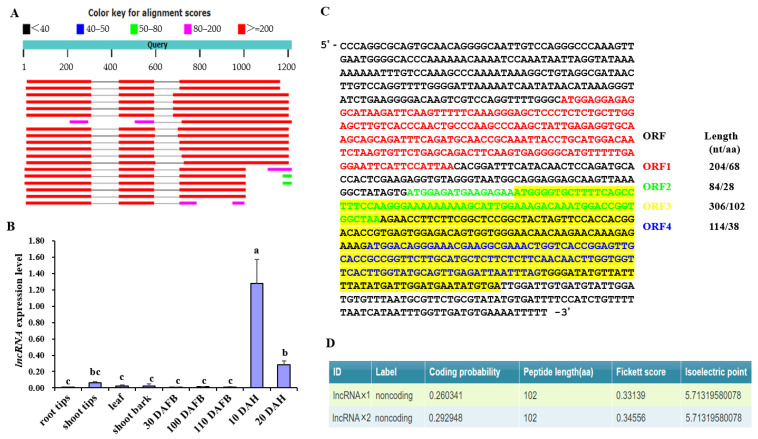
Identification of a *lncRNA* in the promoter of *MdPG1*. (**A**) Blast search showed homologs of the insertion sequence containing two introns and three exons. (**B**) Relative transcript levels of *lncRNA_PG1_* were determined in the root tips, shoot tips, leaf, shoot bark, and fruit of “Huashuo” by qRT-PCR. Fruits were harvested at 30, 100, and 110 days after full bloom (DAFB). The fruit harvested at 110 DAFB were stored at room temperature for 10 and 20 days after harvest (DAF). The error bars show standard deviations of two biological replicates. Significant difference at *p* < 0.05 level is indicted by different lowercase letters based on Fisher’s Least Significant Difference (LSD) test. (**C**) Four short ORFs were predicted in the *lncRNA_PG1_* cDNA and marked with four different colors. ORF length is shown on the right panel. (**D**) The coding potential of *lncRNA_PG1_* was predicted by the Coding Potential Calculator (http://cpc2.gao-lab.org, accessed on 1 March 2021) [62].

The 1.3 kb insert identified above showed a high level of sequence homology to a previously identified but not characterized lncRNA in apple and pear when it was Blast searched against Genbank databases [57] (Table 1). The *lncRNA* gene contained two introns and three exons (Figure 3A). qRT-PCR analyses showed that the *lncRNA* was transcribed in mature apple fruit tissues after harvest (Figure 3B). The transcript sequence of the *lncRNA* was PCR-amplified from cDNA of “Huashuo” fruit flesh and cloned for sequencing. Of the four sequenced clones, one contained a 911 bp and the other three contained an 894 bp cDNA fragment (Figure S3). As “Huashuo” was shown to be homozygous for the lncRNA gene, identification of two cDNA fragments with different length indicated alterative splicing. The *lncRNA* transcript was analyzed for the presence of open reading frames (ORFs). Four short ORFs were found, ORF1–4, potentially encoding peptides of 68, 28 102, and 38 amino acids in length, respectively. ORF4 is enclosed within the sequence of ORF3 but uses a different reading frame (Figure 3C). All protein sequences of these ORFs had no significant similarity to any other proteins as determined by BLAST [57]. Furthermore, we used this sequence to BLAST search GDDH13 (v1.1) [58] and HFTH (v1.0) [59] apple reference genomes [60]. The search identified five homologs of this sequence with 96.25% to 99.55% homology and located on chromosome 0, 1, 4, 5, and 11 (Table S3), but not located on chromosome10, suggesting that there was no insertion in the promoter region of MdPG1 (chromosome10) in “Golden Delicious” and “Hanfu” cultivar. The four homologs were PCR-amplified from genomic DNA of “Huashuo”. The locus was heterozygous on chromosome 0 and 1, homozygous on chromosome 5 and 11 (Table S4). Sequence alignments showed that there were several SNPs and indels among these sequences (Figure S4), so that these homologs can be distinguished from each other. According to the apple lncRNA database information (https://www.tobaccodb.org/plncdb/, accessed on 1 March 2023) [61], six highly similar sequences were annotated as putative nonprotein-coding genes (Figure S5). The protein-coding potential of the *lncRNA* was predicted as non-coding by the Coding Potential Calculator (http://cpc2.gao-lab.org, accessed on 1 March 2021) (Figure 3D) [62], further suggesting that the transcript should be considered as an lncRNA, which was named as *lncRNA_PG1_*.

The expression pattern of *lncRNA_PG1_* was further examined in different apple tissues including root tips, shoot tips, leaf, shoot bark, and fruit flesh of “Huashuo” using qRT-PCR analysis. *LncRNA_PG1_* transcript was detected in fruit at 10 and 20 days after harvest (DAH), with the highest level at 20 DAH. Conversely, *lncRNA_PG1_* transcript was hardly detected in root tips, shoot tips, leaf, shoot bark, or fruits before harvest at 30, 100 and 110 days after full bloom (DAFB) (Figure 3B). These results indicated that *lncRNA_PG1_* was expressed in fruit after harvest.

### 2.4. lncRNA_PG1_ Inhibited MdPG1 Promoter Activity

**Figure 4:**
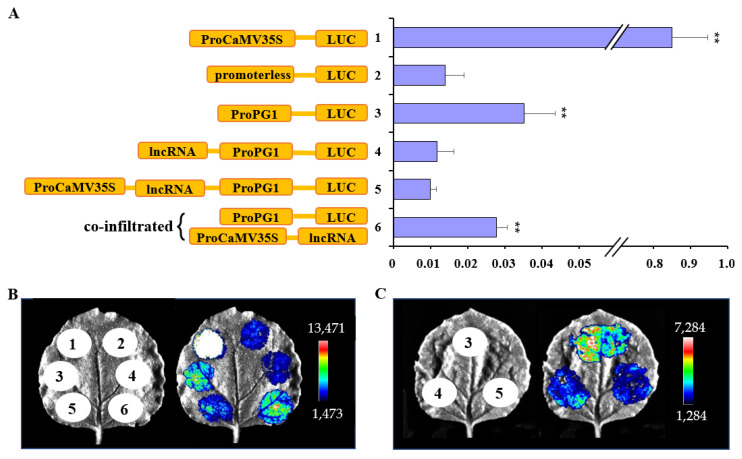
Luciferase activity assay of *MdPG1* promoter. (**A**) The program shows Luc/Ren ratios in N. benthamiana leaves that were transiently transformed by infiltration of Agrobacterium containing five different constructs (1–5) or a combination of two constructs (6). Error bars represent standard deviation of three biological replicates. Asterisks indicate significant differences to promoterless-LUC control (2) by *t*-test: ** *p* < 0.01. (**B**,**C**) Luciferase imaging assays show the luminescence signals in the benthamiana leaves transformed with the same constructs as described in (**A**). The different colors indicate the luminescence intensity.

To directly verify the importance of *lncRNA_PG1_* in suppressing *MdPG1* expression, the *MdPG1* promoter (2.5 kb with SNP^A^) was fused to the LUC reporter gene in pGreenII-0800-LUC to form the *ProPG1-LUC* reporter construct. The luciferase activity from this reporter construct in tobacco leaves was compared to that from *CaMV35S-LUC* and *promoterless-LUC* construct after Agrobacterium infiltration. *CaMV35S-LUC* construct as a positive control showed an extremely high level of luciferase activity. Conversely, the *promoterless-LUC* construct as a negative control showed low basal activity. The *ProPG1-LUC* construct showed significantly higher luciferase activity than the *promoterless-LUC* construct (*p* < 0.01) (Figure 4A). However, when the *lncRNA_PG1_* was linked to the upstream to the *MdPG1* promoter, luciferase activity of the *lncRNA_PG1_-ProPG1-LUC* construct was reduced to the level similar to that of the *promoterless-LUC* construct (Figure 4A). Therefore, *lncRNA_PG1_* has a significant repression effect on the activity of the *MdPG1* promoter. This inhibitory effect was slightly enhanced when *CaMV35S-lncRNA_PG1_* was linked to the upstream to the *MdPG1* promoter in the construct *CaMV35S-lncRNA_PG1_-ProPG1-LUC* (Figure 4A).

To test whether the function of the *lncRNA_PG1_* is related to its *cis*-location, *CaMV35S-lncRNA_PG1_* in a separate vector was co-infiltrated together with *ProPG1-LUC* into *Nicotiana benthamiana* leaves. This co-infiltration did not reduce the luciferase activity driven by the *MdPG1* promoter (Figure 4A). The above experiments were repeated using an imaging system to reveal luciferase activity in tobacco leaves. The luminescence signal of *CaMV35S-LUC* construct was strongest, followed by *ProPG1-LUC* alone or co-infiltration of *35S-lncRNA_PG1_*-*ProPG1-LUC*. The signal was weak for the other three constructs, *promoterless-LUC*, *lncRNA_PG1_-ProPG1-LUC*, and *CaMV35S-lncRNA_PG1_-ProPG1-LUC* (Figure 4B). The similar results were obtained by using only threes constructs to compare the *ProPG1-LUC* to *lncRNA_PG1_-ProPG1-LUC* or *CaMV35S-lncRNA_PG1_-ProPG1-LUC* (Figure 4C). These results together indicate that *lncRNA_PG1′_*s inhibitory effect on *MdPG1* promotor depends on its *cis*-location to the gene promoter.

### 2.5. lncRNA_PG1_ Inhibited NtCHLH Promoter Activity in Transgenic Tobacco Plants

**Figure 5:**
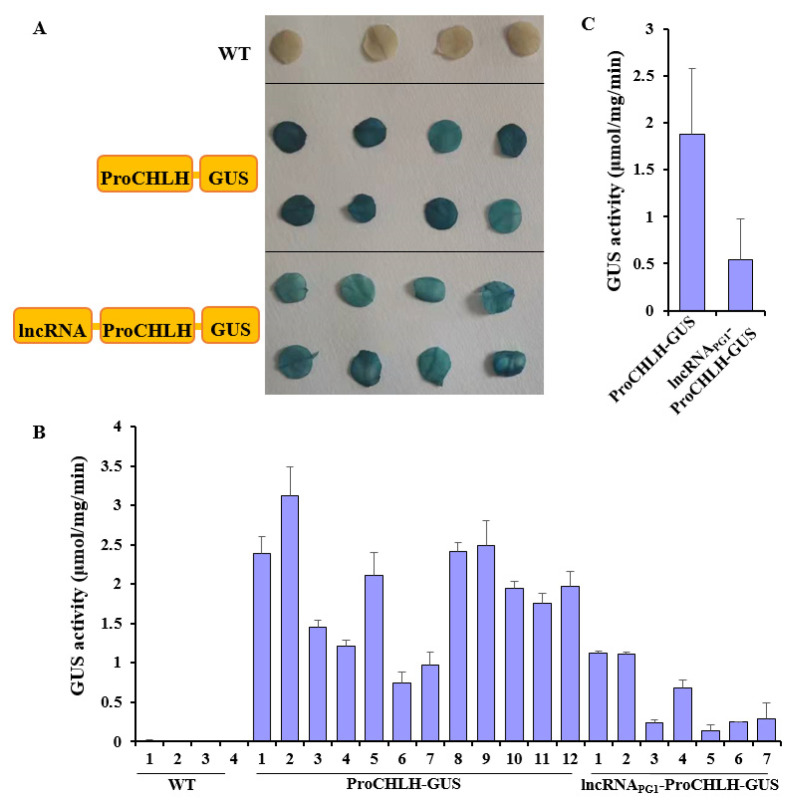
*lncRNA_PG1_* inhibited *NtCHLH* promoter activity in transgenic tobacco plants. (**A**) Leaf discs images show GUS staining results of four wide-type (WT) plants and eight stable transgenic lines harboring the *ProCHLH-GUS* or *lncRNA_PG1_*-*ProCHLH-GUS* construct. (**B**) GUS activities were determined in leaves of four WT plants, 12 *ProCHLH-GUS*, and seven *lncRNA_PG1_*-*ProCHLH-GUS* transgenic lines. The results represent means of three technical replicates. Error bars indicate standard deviation. (**C**) Average GUS activity of 12 *ProCHLH-GUS* lines is compared with the average of seven *lncRNA_PG1_*-*ProCHLH-GUS* lines.

To test whether *lncRNA* inhibits promoter activity of other genes, we generated stable transgenic tobacco plants expressing the β-glucuronidase (GUS) report under the control of the promoter of the gene encoding Mg-chelatase H subunit (*NtCHLH*) (*ProCHLH*-*GUS*) or the *lncRNA_PG1_* connected upstream of *ProCHLH* (*lncRNA _PG1_-ProHLH*-*GUS*) (Figure 5A). *NtCHLH* was chosen because it encodes a key enzyme involved in chlorophyll synthesis and its promoter drives strong gene expression in tobacco leaves. For each construct, eight independent T_0_ transgenic plants were examined by GUS staining. Blue GUS staining was detected in leaf tissues from the eight transgenic plants of each construct but not in leaf tissues from four WT plants. The blue staining was stronger in leaves containing the *ProCHLH*-*GUS* construct than in leaves containing the *lncRNA_PG1_-ProCHLH*-*GUS* construct (Figure 5A).

Furthermore, GUS activity was quantified in the four WT plants, 12 transgenic lines of *ProHLH*-*GUS*, and seven transgenic lines of *lncRNA_PG1_-ProHLH*-*GUS* by using the 4-Methylumbelliferyl-beta-D-glucuronide (MUG) assay. The four WT plants showed no GUS activity, and 10 of the 12 *ProHLH*-*GUS* transgenic lines showed higher GUS activity than all seven *lncRNA_PG1_-ProHLH*-*GUS* transgenic lines (Figure 5B). The mean GUS activity of the 12 *ProHLH*-*GUS* lines was 3.4 folder higher than that of the seven *lncRNA_PG1_-ProHLH*-*GUS* lines (Figure 5C). Together, these results revealed that *lncRNA_PG1_* reduced but not completely inhibited the promoter activity of *NtCHLH* in tobacco leaves.

## 3. Discussion

### 3.1. An SNP in an ERF-Binding Element of MdPG1 Promoter Causes Changes of MdPG1 mRNA Level

Although DNA markers were developed for different *MdPG1* alleles that were associated with different transcript levels of *MdPG1* and fruit texture properties [22,23,25], the causative genetic variants were still unknown. A previous QTL analysis showed that an SNP in the *MdPG1* coding sequence was associated with fruit firmness. The SNP was heterozygous (G/T) in cultivars with soft fruit, whereas it was homozygous (T/T) in cultivars with firm fruit [19]. Our study showed a similar result with this SNP position (Figure 1C). As the SNPs in the coding sequence are unlikely to cause changes of gene expression, we investigated a potential *cis*-regulatory DNA element that may affect *MdPG1* expression.

We identified variations in the 2.5 kb region upstream start codon of *MdPG1* by comparing the sequences of four apple cultivars. These variations included 3 indels, and 34 SNPs in the promoter sequences of *MdPG1* (Figure S1). One of the SNPs was within an ERF-binding element (Figure 2A) and was shown to be a key variant altering *MdPG1* expression. Promoter elements play an important role in regulating gene expression and any change of sequences in these elements may alter gene expression levels and cause phenotypic variations [63,64]. For example, a 3 bp deletion within a putative W-box element of the *ALMT9* promoter abolishes the binding between the promoter and the transcription factor WRKY42 that is a negative regulator of *ALMT9* expression, and promotes a high level of malate accumulation in tomato fruit [65]. An A/G SNP within the TCT-motif of the *GBP1* promoter increases the *GBP1* expression levels and leads to earlier flowering time and maturity in soybean [66]. An A/G SNP created a TCA element in the *PbrmiR397a* promoter inducing the *PbrmiR397a* expression and reducing the lignin content and stone cell number in pear fruit [67]. Our result added another example for SNP variation in promoter affecting gene expression.

### 3.2. MdCBF2 Regulates MdPG1 Expression by Binding to the ERF Element

After showing that the SNP^C^ variant reduces the binding of the transcription factor MdCBF2, thus reducing *MdPG1* expression, we carried out experiments to answer how the SNP may affect *MdPG1* expression. Based on the knowledge that *MdPG1* gene expression responds to ethylene signals and the SNP is located in an ERF-binding element, we decided to test whether the SNP changes the binding ability of EILs and ERFs. After testing two EILs and two ERFs, we showed that the ERF transcription factor MdCBF2 could strongly bind to and activate the promoter fragments containing the SNP^A^ but not the promoter containing the SNP^C^ (Figure 2). *MdPG1* is known to be *trans*-activated by MdCBF2 from a previous study [12]. Our study further showed that this activation is dependent on a correct sequence of the ERF-binding element in the promoter of *MdPG1*. In *Arabidopsis*, it has been shown that the ethylene signal cascade ultimately leads to the stabilization of the primary responsive transcription factors EIN3/EILs, which have been shown to bind and active the secondary responsive transcription factors ERFs [68]. The AP2/ERF family is a large group of plant-specific transcription factors involved in plant developmental processes and multiple environmental stimuli. This family also includes the famous members CBFs, which are strongly cold-regulated [69]. AtEIN3 acts as a negative regulator of freezing stress by directly regulating the expression of *AtCBF1*–*AtCBF3* [70]. The data presented here show that EIL2 and EIL3 hardly transactivate the *MdPG1* promoter in a transient system. There is a possibility that the endogenous tobacco EILs are inhibited, so that CBF2 can play its role in transactivating *MdPG1.* Further work will be needed to explore whether other transcription factors are also involved in the regulation of *MdPG1* expression, and whether they work in synergy with MdCBF2.

ERFs are known to bind to *cis*-acting elements, such as GCC box (GCCGCC), DRE (TACCGACAT), and CTR (TGGCCGAC) motifs, to regulate gene expression. For example, in apple, MdERF2 suppresses *MdACS1* expression, whereas MdERF3 promotes its expression by binding to the DRE motif in its promoter. In tomato, LeERF2 activates the expression of ethylene biosynthesis genes by binding to the GCC box or DRE motif in their promoters. In papaya, CpERF9 represses CpPME1/2 and *CpPG5* expression by binding to the GCC box in their promoters [8]. In peach, PpERF3 promotes the expression of the ABA biosynthesis gene *PpNCED2/3* by binding to the ERF-binding motif in its promoter [9]. Our study identified and functionally tested a new ERF-binding element in apple.

### 3.3. LncRNA_PG1_ cis-Regulates Nearby Genes

Although lncRNAs may *cis*- or *trans*-regulate gene expression [71], we demonstrated here that *lncRNA_PG1_ cis*-regulated the expression of the nearby gene *MdPG1* (Figure 4). Luciferase transient expression assay in tobacco leaves showed that luciferase activity was reduced by fusing *lncRNA_PG1_* to the *MdPG1* promoter driving luciferase coding sequence. However, this reduction in luciferase activity was not detected when *lncRNA_PG1_* was separated from the *MdPG1* promoter by using two different constructs (Figure 4A,B). Our results further showed that *lncRNA* could also reduce the promoter activity of *NtCHLH* in stable transgenic tobacco plants when the *lncRNA* and promoter were fused within one gene construct (Figure 5). The data presented in this study suggest that *lncRNA_PG1_* act as a *cis*-regulator.

There are at least three potential mechanisms for lncRNAs *cis*-acting gene expression. First, lncRNAs may modulate the action of the protein-coding genes involved in epigenetic patterning and chromatin remodeling or function as scaffolds. A plant lncRNA, *COLDAIR*, acts in the same way as *Xist* [72], which serves as scaffolds for the recruitment of PRC2 complexes to specific loci and induces epigenetic silencing [73]. Second, transcription or splicing of lncRNA may modulate gene expression in *cis*. This mechanism is exemplified by lncRNA *Blustr* (bivalent locus [Sfmbt2] is upregulated by splicing and transcribing an RNA). Both deletion and insertions in the promoter of the *Blustr* substantially reduced the expression of a neighboring gene [74,75]. Third, lncRNA locus may contain DNA elements acting as an enhancer to regulate neighboring gene expression. Examples for this type of lncRNAs include *lincRNA-p21* in human [76], *Bendr* in mouse [74], and *cis-NAT_PHO1;2_* in rice [40]. Their functions can largely be ascribed to conventional *cis*-acting DNA elements embedded within their gene body sequences.

In our case, the presence of a high level of *lncRNA_PG1_* transcripts may not be required for its inhibitory effect. The inhibitory effect on the *MdPG1* promotor was shown when a promotorless *lncRNA_PG1_* was used but was not further enhanced when *lncRNA_PG1_* was expressed from the strong *CaMV35S* promoter (Figure 4A). Studies have shown that some lncRNA functions could result from processes that are not mediated by the lncRNA transcripts themselves, but instead involved general processes associated with their production, including enhancer-like activity of gene promoters, the process of transcription, and the splicing of the transcript [74]. It is also worth noting that, in the establishment of transcriptional gene silencing by *cis*-acting lncRNAs, continuous transcription might be more important than the production of mature RNA [74,77,78].

## 4. Materials and Methods

### 4.1. Cloning MdPG1 Promoter and Coding Sequence

Genomic DNA was extracted from apple cultivar “Meiba”, “Huahong”, “Huaxing” and “Huashuo” using a DNeasy Plant Mini Kit (Tiangen, Beijing, China) and used to amplify the DNA fragment of two *MdPG1* alleles. The overlapping fragments covering the coding and promoter regions of each allele were amplified using two pairs of gene-specific primers PG-F1 and PG-R1, PG-F2 and PG-R2 (Table S5). The amplified products were separated by electrophoresis on 1% agarose gel and photographed under UV illumination, and the amplified fragments of each *MdPG1* allele in the four apple cultivars were cloned into the p-blunt vector (TranGen, Beijin, China) and finally sequenced. Multiple alignments of DNA sequences that cover the promoter and coding regions of *MdPG1* were performed using Geneious software v9.1.4 [79].

### 4.2. Cis-Element Analysis

The AP2/ERF and EIN3/EIL cis-elements in the 2.5 kb promoter sequence of both MdPG1 alleles were identified using the Plantpan2.0 database (http://plantpan2.itps.ncku.edu.tw, accessed on 1 June 2020) [56] and CIS-BP database (http://cisbp.ccbr.utoronto.ca/TFTools.php, accessed on 1 March 2023) [80]. We accessed the MdPG1 promoter via the promoter analysis function in Plantpan2.0 database. Transcription factor binding sites were predicted from all species in the database. In addition, we directly used sequences of two alleles and compared their recognition scores through estimating respective false positive rates. Scan two sequences for differential TF binding tool were used and Malus_domestica specie was selected in CIS-BP database. This method identifies TFs with maximum E-score (which provide comprehensive scores for all possible eight base sequences) >0.45 for one allele, and maximum E-score < 0.45 for the other.

### 4.3. Dual-Luciferase Reporter Assay

A 2.5 kb fragment (upstream of the ATG start codon) of the *MdPG1* promoter containing the ERF-binding SNP was amplified using PCR primers listed in Table S5, as described above. A fusion promoter of a 0.3 kb *MdPG1* fragment (between −133 to −438 from the ATG start codon, containing the SNP site) and the minimal CaMV 35S promoter sequence (−46 to −1) [81] were synthesized by Tsingke Biotechnology Co., Ltd. (Beijing, China). These four promoters (2.5 kb with SNP^A^ or SNP^C^, and 0.3 kb plus minimal CaMV 35S with SNP^A^ or SNP^C^) were cloned into the reporter vector pGreenII 0800-LUC. The full-length coding sequence of *MdCBF2*, *MdAP2D32*, *MdEIL2*, and *MdEIL3* were amplified from fruit flesh cDNA of “Huahong” and cloned into the effector vector pSAK778 under the control of the CaMV35S promoter. The reporter and effector vectors were separately transferred into *Agrobacterium tumefaciens* GV3101 (pSoup) cells. A mixture of *Agrobacterium* cells containing the reporter and effector constructs (1:8, reporter: effector) was used to infiltrate young *Nicotiana benthamiana* leaves [82]. After infiltration for 72 h, LUC (Firefly luciferase) and REN (Renilla luciferase) activities were assayed using the Dual-Luciferase^®^ Reporter Assay System (Promega, Madison, WI, USA) and the SpectraMax^@^i3x Platform (MOLECULAR DEVICES, San Jose, CA, USA). Six biological replicates were performed for each assay.

### 4.4. Yeast One-Hybrid Assay

The yeast one-hybrid assay was performed using a Matchmaker^TM^ Gold Yeast One-Hybrid Library Screening System Kit (Clontech, San Francisco, CA, USA). The full-length coding sequence of *MdCBF2* was cloned into the pGADT7 vector, and the 2.5 kb and 0.3 kb promoter sequences of *MdPG1* with SNP^A^ or SNP^C^ were cloned into pAbAi vector to construct the bait vectors. The bait vector was introduced into Y1HGold yeast cells that were subsequently selected on SD/-Ura medium. The yeast cells containing the *MdPG1 promoter*-pAbAi bait vector were re-transformed with the MdCBF2-pGADT7 prey vector and selected on SD/-Leu medium. Yeast colonies that were confirmed to contain both bait and prey vectors were grown to reach a cell density of OD_600_ 1.5 and then diluted in 5-fold gradient. After the dilution, 5μL of suspension cells was spotted on SD/-Leu medium with or without the addition of AbA (aureobasidin A) and incubated for 3 to 5 days at 30 °C.

### 4.5. Analyzing lncRNA_PG1_ Sequence and Expression Pattern

Fruit of “Huashuo” were harvested at 30, 100, and 110 days after full bloom (DAFB). The fruit harvested at 110 DAFB were stored at room temperature for 10 and 20 days after harvest (DAF). For quantitative reverse transcription polymerase chain reaction (qRT-PCR) analysis, different tissues including root tips, shoot tips, leaf, shoot bark and fruits (30 DAFB, 100 DAFB, 110 DAFB, 10 DAF and 20 DAF) were collected from “Huashuo”. Total RNA was isolated using a Total RNA Kit (Sangon, Shanghai, China) according to the manufacturer’s instructions. First-strand cDNA was synthesized from the RNA samples with a Reverse Transcriptase Kit (Tiangen, Beijing, China). Real-time PCR was performed using SYBR Green PCR Master Mix on Roche LightCycler 480 system (Roche LightCycler, Roche, Basel, Switzerland). Relative gene expression was analyzed using apple reference genes *MdEF1a* and *MdActin* [83]. Normalization factors were calculated by taking the geometric mean of the two reference genes as determined by geNorm v3.4 [84]. The primers used for qRT-PCR were listed in Table S5.

*lncRNA_PG1_* cDNA was amplified from mRNA of “Huashuo” fruit at 10 DAF using primers lncRNA-F2 and lncRNA-R2 (Table S5). The amplified fragments were cloned into the p-blunt vector and sequence as described above. The open reading frames (ORFs) of the lncRNA*_PG1_* were searched by ORF finder (https://www.ncbi.nlm.nih.gov/orffinder/, accessed on 1 March 2021) [57], and the coding potential was predicted using the Coding Potential Calculator (http://cpc2.gao-lab.org, accessed on 1 March 2021) [62]. Multiple sequence alignments of *lncRNA_PG1_* DNA and *lncRNA_PG1_* cDNA sequences were performed using Geneious software v9.1.4 [79].

### 4.6. Blast Search of lncRNA_PG1_

The National Center for Biotechnology Information (NCBI) BLAST server (https://blast.ncbi.nlm.nih.gov/Blast.cgi, accessed on 1 April 2020) [57] and GENOME DATABASE FOR ROSACEAE (GDR) database (www.rosaceae.org, accessed on 1 March 2021) [60] were used for searching the homologs of *lncRNA_PG__1_*. These sequences in apple were amplified from genomic DNA of “Huashuo” using five pairs of gene-specific primers listed in Table S5. Multiple sequence alignments were performed using Geneious software v9.1.4 [79].

### 4.7. Transient Assay of MdPG1 Promoter in Nicotiana benthamiana

To verify the inhibitory effect of *lncRNA_PG1_* on the *MdPG1* promoter, dual-luciferase reporter assays were carried out using six gene constructs. The 2.5 kb *MdPG1* promoter fragment was fused to the LUC report gene in pGreenII 0800 [82] to form the *ProPG1*-*LUC* construct. The *CaMV35S*-*lncRNA_PG1_* construct was generated by cloning 1.3 kb *lncRNA_PG1_* gene sequence into pSAK778 [85] between the *CaMV35S* and OCS terminator in a sense orientation. *CaMV35S*-*lncRNA_PG1_* and *lncRNA_PG1_* were separately inserted into the *ProPG1-LUC* vector to form two vectors, *CaMV35S*-*lncRNA_PG1_-ProPG1*-*LUC* and *lncRNA_PG1_-ProPG1*-*LUC*. The positive and negative control vectors, *CaMV35S*-*LUC* and *promoterless*-*LUC*, were previously constructed [86]. The above constructs were transferred into *Agrobacterium tumefaciens* GV3101 cells by electroporation and were infiltrated into *Nicotiana benthamiana* leaves, as described previously [87]. At 72 h after infiltration, Firefly and *Renilla* luciferase activities were assayed using the Dual-Luciferase^®^ Reporter Assay System (Promega, USA) according to the manufacturer’s instructions, and the activity of the *MdPG1* promoter was expressed as the ratio of firefly to *Renilla* luciferase activities. The luciferase assay was carried out using SpectraMax^@^i3x Platform (MOLECULAR DEVICES, San Jose, CA, USA) in three independent experiments. In each experiment, three biological replicates were analyzed. The luminescence signal was also detected on images by using a Tanon-5200Multi machine (Biotanon, Shanghai, China). The primers used for making the gene constructs were listed in Table S5.

### 4.8. Tobacco Transformation

The 1.6 kb promoter sequence of Mg-chelatase H subunit (*NtCHLH*) was amplified from genomic DNA of *Nicotiana tabacum* “NC89” and inserted into pBI121-GUS vector [88] to replace the *CaMV35S* promoter. The sequence of *lncRNA_PG1_* was inserted upstream of the *NtCHLH* promoter sequence into the above construct. The resulting *ProCHLH*-*GUS* or *lncRNA_PG1_-ProCHLH*-*GUS* constructs were transferred into *Agrobacterium tumefaciens* GV3101 cells separately by electroporation, and then into tobacco “NC89” plants using a leaf disk transformation protocol as described previously [89]. The transformed tobacco plants were selected using 200 mg/L kanamycin. Transgenic and wide-type (WT) tobacco plants were grown in a growth chamber (temperature of 23 °C, 16 h light/8 h dark, and ~80% relative humidity).

### 4.9. Histochemical GUS Staining and Fluorometric Assays

For histochemical staining of GUS, 1 cm leaf discs of transgenic tobacco leaves were immediately treated with 5-bromo-4-chloro-3-indolyl b-D-glucuronide (X-Gluc) at 37 °C for 24 h using the Gusblue kit (Huayueyang Biotech Co., Ltd., Beijing, China). Stained samples were bleached with 70% (*v*/*v*) ethanol to remove the chlorophyll before photographing. GUS activity was determined by measuring the fluorescence of 4-methylumbelliferone produced by GUS cleavage of 4-methylumbelliferyl-β-D-glucuronide using the GUS Gene Quantitative Detection Kit (Coolaber, Beijing, China) according to the manufacturer’s instructions. The protein concentration in the supernatant was determined using the Bradford procedure with bovine serum albumin (Sigma, St. Louis, MO, USA) as a standard.

### 4.10. Statistical Analyses

All analyses were carried out using analysis of variance (ANOVA) of statistical analysis system, and SPSS 17.0 Statistics (SPSS Inc., Chicago, IL, USA) [90]. Significance levels in comparison of the means were determined by *p* < 0.01 (Student’s *t*-test). Post hoc differences between means were determined using Fisher’s least significant difference (LSD) test [91] at the 5% significance level.

## 5. Conclusions

**Figure 6:**
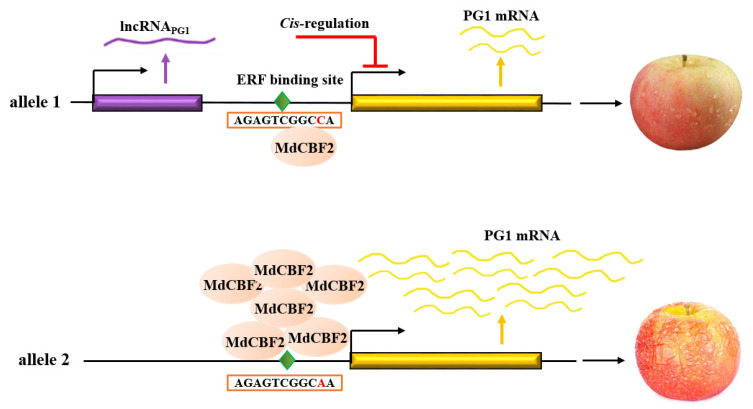
Proposed mechanism of *MdPG1* allele-specific expression in apple fruit softening. MdCBF2 directly bind to the promoter of the SNP^A^ allele of *MdPG1* to enhance *MdPG1* expression. *lncRNA_PG1_* suppresses *MdPG1* mRNA level through a *cis*-regulation mechanism. The diamond represents an ERF-binding element.

In conclusion, despite *MdPG1* playing an important role in apple fruit softening, the molecular mechanism regulating *MdPG1* remains largely unclear. Here, we have identified a causative SNP within an ERF-binding element in the *MdPG1* promoter to affect *MdPG1* expression through altering the binding of MdCBF2 (Figure 6). In addition, we also identified a novel lncRNA, *lncRNA_PG1_*, which is located in the promoter of *MdPG1*, and demonstrated that *lncRNA_PG1_* negatively regulates the expression of *MdPG1* through *cis*-regulation. These findings establish a novel lncRNA_PG1_-MdPG1 regulatory network in apple fruit softening (Figure 6).

## Figures and Tables

**Table 1:** Hits of Blast search GenBank using the 1.3 kb insertion sequence.

Description	Query Cover	Per. Ident	Accession
Malus domestica, transcript variant X1, ncRNA	67%	99.12%	XR_003773574.1
Malus domestica, ncRNA	68%	98.02%	XR_528796.3
Pyrus × bretschneideri, transcript variant X3, ncRNA	72%	92.72%	XR_669814.2
Pyrus × bretschneideri, transcript variant X4, ncRNA	72%	92.54%	XR_001952184.1
Pyrus × bretschneideri, transcript variant X4, ncRNA	73%	92.55%	XR_001954604.1
Pyrus × bretschneideri, transcript variant X5, ncRNA	73%	92.36%	XR_001952185.1
Pyrus × bretschneideri, transcript variant X1, ncRNA	71%	92.59%	XR_669811.2
Pyrus × bretschneideri, transcript variant X1, ncRNA	71%	92.40%	XR_665590.2
Pyrus × bretschneideri, transcript variant X2, ncRNA	70%	92.50%	XR_669812.2

## Data Availability

Not applicable.

## Supplementary Materials

The following supporting information can be downloaded at: https://www.mdpi.com/article/10.3390/plants12071452/s1, Figure S1. Sequence alignments of *MdPG1* alleles of “Meiba”, “Huahong”, “Huaxing” and “Huashuo”. (**A**–**H**) The sequences covering the promoter (**A**,**C**,**E**,**G**) and coding regions (**B**,**D**,**F**,**H**) of two alleles of *MdPG1* were PCR-amplified from genomic DNA of “Meiba” (**A**,**B**), “Huahong” (**C**,**D**), “Huaxing” (**E**,**F**) and “Huashuo” (**G**,**H**). The amplified fragments were cloned and sequenced. Pairwise sequence alignment of the promoter and gene body regions of *MdPG1* was performed using Geneious software v9.1.4. The start (ATG) and stop codon (TAA) of *MdPG1* gene are shown in red box. AP2/ERF and EIN/EIL3 binding elements are highlighted with yellow and red color, respectively. An SNP within an ERF-binding element is using blue color. The SNPs in the 5′ UTR and CDS of two *MdPG1* alleles are highlighted. The red sequence represents the 1.3 kb insertion (**A**,**E**,**G**); Figure S2. Phylogenetic analysis of the EIL and AP2 proteins. Rooted neighbor-joining phylogenetic tree were constructed using protein sequences of members of the EIL (**A**) and AP2 (**B**) family from apple, *Arabidopsis* and kiwifruit. The GenBank or Arabidopsis TAIR accession numbers or Gene ID in GDR used are listed in Table S6; Figure S3. cDNA sequence of two *lncRNA_PG1_* transcript isoforms. Two *lncRNA* cDNA sequences of 911 bp and 894 bp were cloned from “Huashuo” fruit flesh. *lncRNA* DNA and *lncRNA* cDNA sequences were aligned using Geneious software v9.1.4. The blue diamond showed the three exons of *lncRNA*; Figure S4. Sequence alignments of lncRNA_PG1_ homologs. LncRNA_PG1_ homolog sequences were identified from on chromosome 0, 1, 5, 11 and 10 by Blast search, PCR-amplified from genomic DNA of “Huashuo”, cloned, sequenced, and aligned using Geneious software v9.1.4; Figure S5. Six highly similar sequences of lncRNA were annotated as putative nonprotein-coding genes. According to the apple lncRNA database information, six highly similar sequences of lncRNA were annotated as putative nonprotein-coding genes. PLnc DB (https://www.tobaccodb.org/plncdb/, accessed on 1 March 2023); Table S1. Information of the four apples; Table S2. Scan two alleles of *MdPG1* for differential TF binding; Table S3. *LncRNA_PG1_* homologs identified in apple genome; Table S4. Genotyping of *lncRNA_PG1_* homologs in eight apple cultivars; Table S5. The list of primer sequences used in this study; Table S6. Accession numbers of proteins to generate the phylogenetic tree.

## References

[1] Oeller P.W., Lu M.W., Taylor L.P., Pike D.A., Theologis A. (1991). Reversible inhibition of tomato fruit senescence by antisense RNA. Science.

[2] Liu M.C., Pirrello J., Chervin C., Roustan J.P., Bouzayen M. (2015). Ethylene control of fruit ripening: Revisiting the complex network of transcriptional regulation. Plant Physiol..

[3] Johnston J.W., Kularajathaven G., Paul P., Wang M., Schaffer R.J. (2009). Co-ordination of early and late ripening events in apples is regulated through differential sensitivities to ethylene. J. Exp. Bot..

[4] Sunako T., Sakuraba W., Senda M., Akada S., Ishikawa R., Niizeki M., Harada T. (1999). An allele of the ripening-specific 1-aminocyclopropane-1-carboxylic acid synthase gene (*ACS1*) in apple fruit with a long storage life. Plant Physiol..

[5] Costa F., Stella S., Van de Weg W.E., Guerra W., Cecchinel M., Dallavia J., Koller B., Sansavini S. (2005). Role of the genes *Md-ACO1* and *Md-ACS1* in ethylene production and shelf life of apple (*Malus domestica* Borkh). Euphytica.

[6] Zhu Y.M., Barritt B.H. (2008). *Md-ACS1* and *Md-ACO1* genotyping of apple (*Malus* × *domestica* Borkh.) breeding parents and suitability for marker-assisted selection. Tree Genet. Genomes.

[7] Marić S., Lukić M. (2014). Allelic polymorphism and inheritance of *MdACS1* and *MdACO1* genes in apple (*Malus* × *domestica* Borkh.). Plant Breed..

[8] Fu C.C., Han Y.C., Qi X.Y., Shan W., Chen J.Y., Lu W.J., Kuang J.F. (2016). Papaya CpERF9 acts as a transcriptional repressor of cell-wall-modifying genes *CpPME1/2* and *CpPG5* involved in fruit ripening. Plant Cell Rep..

[9] Wang X.B., Zeng W.F., Ding Y.F., Wang Y., Niu L., Yao J.L., Pan L., Lu Z.H., Cui G.C., Li G.H. (2019). PpERF3 positively regulates ABA biosynthesis by activating *PpNCED2/3* transcription during fruit ripening in peach. Hortic. Res..

[10] Xiao Y.Y., Chen J.Y., Kuang J.F., Wei S., Hui X., Jiang Y.M., Lu W.J. (2013). Banana ethylene response factors are involved in fruit ripening through their interactions with ethylene biosynthesis genes. J. Exp. Bot..

[11] Ohta M., Matsui K., Hiratsu K., Shinshi H., Ohme-Takagi M. (2001). Repression domains of class II ERF transcriptional repressors share an essential motif for active repression. Plant Cell.

[12] Tacken E., Ireland H., Gunaseelan K., Karunairetnam S., Wang D., Schultz K., Bowen J., Atkinson R.G., Johnston J.W., Putterill J. (2010). The role of ethylene and cold temperature in the regulation of the apple *POLYGALACTURONASE1* gene and fruit softening. Plant Physiol..

[13] Li T., Jiang Z.Y., Zhang L.C., Tan D.M., Wei Y., Yuan H., Li T.L., Wang A.D. (2016). Apple (*Malus domestica*) MdERF2 negatively affects ethylene biosynthesis during fruit ripening by suppressing *MdACS1* transcription. Plant J..

[14] Hu Y.N., Han Z.Y., Sun Y.Q., Wang S., Wang T., Wang Y., Xu K.N., Zhang X.Z., Xu X.F., Han Z.H. (2020). ERF4 affects fruit firmness through TPL4 by reducing ethylene production. Plant J..

[15] Brummell D.A., Harpster M.H. (2001). Cell wall metabolism in fruit softening and quality and its manipulation in transgenic plants. Plant Mol. Biol..

[16] Candelas P., Sara P., Morris V.J., Kirby A.R., Quesada M.A., Mercado J.A. (2014). Fruit softening and pectin disassembly: An overview of nanostructural pectin modifications assessed by atomic force microscopy. Ann. Bot..

[17] Wang D.D., Yeats T.H., Uluisik S., Rose J.K.C., Seymour G.B. (2018). Fruit softening: Revisiting the role of pectin. Trends Plant Sci..

[18] Sénéchal F., Wattier C., Rustérucci C., Pelloux J. (2014). Homogalacturonan-modifying enzymes: Structure, expression, and roles in plants. J. Exp. Bot..

[19] Costa F., Peace C.P., Stella S., Serra S., Musacchi S., Bazzani M., Sansavini S., Van de Weg W.E. (2010). QTL dynamics for fruit firmness and softening around an ethylene-dependent polygalacturonase gene in apple (*Malus* × *domestica* Borkh.). J. Exp. Bot..

[20] Atkinson R.G., Schröder R., Hallett I.C., Cohen D., MacRae E.A. (2002). Overexpression of polygalacturonase in transgenic apple trees leads to a range of novel phenotypes involving changes in cell adhesion. Plant Physiol..

[21] Atkinson R.G., Sutherland P.W., Johnston S.L., Gunaseelan K., Hallett I.C., Mitra D., Brummell D.A., Schröder R., Johnston J.W., Schaffer R.J. (2012). Down-regulation of *POLYGALACTURONASE1* alters firmness, tensile strength and water loss in apple (*Malus* × *domestica*) fruit. BMC Plant Biol..

[22] Longhi S., Cappellin L., Guerra W., Costa F. (2013). Validation of a functional molecular marker suitable for marker-assisted breeding for fruit texture in apple (*Malus* × *domestica* Borkh.). Mol. Breed..

[23] Poles L., Gentile A., Giuffrida A., Valentini L., Endrizzi I., Aprea E., Gasperi F., Distefano G., Malfa S.L., Costa F. (2020). Role of fruit flesh cell morphology and *MdPG1* allelotype in influencing juiciness and texture properties in apple. Postharvest Biol. Technol..

[24] Longhi S., Moretto M., Viola R., Velasco R., Costa F. (2012). Comprehensive QTL mapping survey dissects the complex fruit texture physiology in apple (*Malus* × *domestica* Borkh.). J. Exp. Bot..

[25] Longhi S., Hamblin M.T., Trainotti L., Peace C.P., Velasco R., Costa F. (2013). A candidate gene based approach validates *Md-PG1* as the main responsible for a QTL impacting fruit texture in apple (*Malus* × *domestica* Borkh). BMC Plant Biol..

[26] Song J.H., Cao J.S., Yu X.L., Xiang X. (2007). *BcMF11*, a putative pollen-specific non-coding RNA from *Brassica campestris* ssp. chinensis. J. Plant Physiol..

[27] Song J.H., Cao J.S., Wang C.G. (2013). *BcMF11*, a novel non-coding RNA gene from Brassica campestris, is required for pollen development and male fertility. Plant Cell Rep..

[28] Huang L., Dong H., Zhou D., Li M., Liu Y.H., Zhang F., Feng Y.Y., Yu D.L., Lin S., Cao J.S. (2018). Systematic identification of long non-coding RNAs during pollen development and fertilization in *Brassica rapa*. Plant J..

[29] Ding J.H., Lu Q., Ouyang Y., Mao H.L., Zhang P.B., Yao J.L., Xu C.G., Li X.H., Xiao J.H., Zhang Q.F. (2012). A long noncoding RNA regulates photoperiod-sensitive male sterility, an essential component of hybrid rice. Proc. Natl. Acad. Sci. USA.

[30] Fan Y.R., Yang J.Y., Mathioni S.M., Yu J.S., Yang X.F., Wang L., Zhang Q.H., Cai Z.X., Xu C.G., Li X.H. (2016). *PMS1T*, producing phased small-interfering RNAs, regulates photoperiod-sensitive male sterility in rice. Proc. Natl. Acad. Sci. USA.

[31] Zhao X.Y., Li J.R., Lian B., Gu H.Q., Li Y., Qi Y.J. (2018). Global identification of Arabidopsis lncRNAs reveals the regulation of *MAF4* by a natural antisense RNA. Nat. Commun..

[32] Swiezewski S., Liu F., Magusin A., Dean C. (2009). Cold-induced silencing by long antisense transcripts of an *Arabidopsis* Polycomb target. Nature.

[33] Wang Y.Q., Fan X.D., Lin F., He G.M., Terzaghi W., Zhu D.M., Deng X.W. (2014). *Arabidopsis* noncoding RNA mediates control of photomorphogenesis by red light. Proc. Natl. Acad. Sci. USA.

[34] Kakar K., Zhang H., Scheres B., Dhonukshe P. (2013). CLASP-mediated cortical microtubule organization guides PIN polarization axis. Nature.

[35] Wunderlich M., Groß-Hardt R., Schöffl F. (2014). Heat shock factor HSFB_2a_ involved in gametophyte development of Arabidopsis thaliana and its expression is controlled by a heat-inducible long non-coding antisense RNA. Plant Mol. Biol..

[36] Bardou F., Ariel F., Simpson C., Romero-Barrios N., Laporte P., Balzergue S., Brown J.S., Crespi M. (2014). Long noncoding RNA modulates alternative splicing regulators in *Arabidopsis*. Dev. Cell.

[37] Wang M.J., Yuan D.J., Tu L.L., Gao W.H., He Y.H., Hu H.Y., Wang P.C., Liu N., Lindsey K., Zhang X.L. (2015). Long noncoding RNAs and their proposed functions in fibre development of cotton (*Gossypium* spp.). New Phytol..

[38] Fedak H., Palusinska M., Krzyczmonik K., Brzezniak L., Yatusevich R., Pietras Z., Kaczanowski S., Swiezewski S. (2016). Control of seed dormancy in Arabidopsis by a *cis*-acting noncoding antisense transcript. Proc. Natl. Acad. Sci. USA.

[39] Franco-Zorrilla J.M., Valli A., Todesco M., Mateos I., Puga M.I., Rubio-Somoza I., Leyva A., Weigel D., García J.A., Paz-Ares J. (2007). Target mimicry provides a new mechanism for regulation of microRNA activity. Nat. Genet..

[40] Jabnoune M., Secco D., Lecampion C., Robaglia C., Shu Q., Poirier Y. (2013). A rice *cis*-natural antisense RNA acts as a translational enhancer for its cognate mRNA and contributes to phosphate homeostasis and plant fitness. Plant Cell.

[41] Sun Y.Q., Hao P.B., Lv X.M., Tian J., Wang Y., Zhang X.Z., Xu X.F., Han Z.H., Wu T. (2020). A long non-coding apple RNA, MSTRG.85814.11, acts as a transcriptional enhancer of *SAUR32* and contributes to the Fe-deficiency response. Plant J..

[42] Qin T., Zhao H.Y., Cui P., Albesher N., Xiong L.M. (2017). A nucleus-localized long non-coding RNA enhances drought and salt stress tolerance. Plant Physiol..

[43] Cui J., Jiang N., Meng J., Yang G.L., Liu W.W., Zhou X.X., Ma N., Hou X., Luan Y. (2019). LncRNA33732-respiratory burst oxidase module associated with WRKY1 in tomato- *Phytophthora infestans* interactions. Plant J..

[44] Cui J., Luan Y.S., Jiang N., Bao H., Meng J. (2017). Comparative transcriptome analysis between resistant and susceptible tomato allows the identification of lncRNA16397 conferring resistance to *Phytophthora infestans* by co-expressing glutaredoxin. Plant J..

[45] Zhang Y., Wang S.N., Li W., Wang S.Y., Hao L., Xu C.R., Yu Y.F., Xiang L., Li T.Z., Jiang F. (2022). A long noncoding RNA *HILinc1* enhances pear thermotolerance by stabilizing *PbHILT1* transcripts through complementary base pairing. Commun. Biol..

[46] Jiang N., Cui J., Shi Y.S., Yang G.L., Zhou X.X., Hou X.X., Meng J., Luan Y.S. (2019). Tomato lncRNA23468 functions as a competing endogenous RNA to modulate NBS-LRR genes by decoying miR482b in the tomato-*Phytophthora infestans* interaction. Hortic. Res..

[47] Yang T., Ma H.Y., Zhang J., Wu T., Song T.T., Tian J., Yao Y.C. (2019). Systematic identification of long noncoding RNAs expressed during light-induced anthocyanin accumulation in apple fruit. Plant J..

[48] Ma H.Y., Yang T., Li Y., Zhang J., Wu T., Song T.T., Yao Y.C., Tian J. (2021). The long noncoding RNA MdLNC499 bridges MdWRKY1 and MdERF109 function to regulate early-stage light-induced anthocyanin accumulation in apple fruit. Plant Cell.

[49] Zhu B.Z., Yang Y.F., Li R., Fu D.Q., Wen L.W., Luo Y.B., Zhu H.L. (2015). RNA sequencing and functional analysis implicate the regulatory role of long non-coding RNAs in tomato fruit ripening. J. Exp. Bot..

[50] Li R., Fu D.Q., Zhu B.Z., Luo Y.B., Zhu H.L. (2018). CRISPR/Cas9-mediated mutagenesis of *lncRNA1459* alters tomato fruit ripening. Plant J..

[51] Zhang G.Y., Chen D.G., Zhang T., Duan A.G., Zhang J.G., He C.Y. (2018). Transcriptomic and functional analyses unveil the role of long non-coding RNAs in anthocyanin biosynthesis during sea buckthorn fruit ripening. DNA Res..

[52] Bai L.J., Chen Q., Jiang L.Y., Lin Y.X., Ye Y., Liu P., Wang X.R., Tang H.R. (2019). Comparative transcriptome analysis uncovers the regulatory functions of long noncoding RNAs in fruit development and color changes of *Fragaria pentaphylla*. Hortic. Res..

[53] Chen Y., Cheng C., Feng X., Lai R., Gao M., Chen W., Wu R. (2021). Integrated analysis of lncRNA and mRNA transcriptomes reveals the potential regulatory role of lncRNA in kiwifruit ripening and softening. Sci. Rep..

[54] Tian Y., Bai S., Dang Z., Hao J., Zhang J., Hasi A. (2019). Genome-wide identification and characterization of long non-coding RNAs involved in fruit ripening and the climacteric in *Cucumis melo*. BMC Plant Biol..

[55] An N., Fan S., Wang Y.B., Zhang L.Z., Gao C., Zhang D., Han M.Y. (2018). Genome-wide identification, characterization and expression analysis of long non-coding RNAs in different tissues of apple. Gene.

[56] Chow C.N., Zheng H.Q., Wu N.Y., Chien C.H., Huang H.D., Lee T.Y., Chiang-Hsieh Y.F., Hou P.F., Yang T.Y., Chang W.C. (2016). PlantPAN 2.0: An update of plant promoter analysis navigator for reconstructing transcriptional regulatory networks in plants. Nucleic Acids Res..

[57] Cooper P.S., Lipshultz D., Matten W.T., McGinnis S.D., Pechous S., Romiti M.L., Tao T., Valjavec-Gratian M., Sayers E.W. (2010). Education resources of the National Center for Biotechnology Information. Brief. Bioinform..

[58] Daccord N., Celton J.M., Linsmith G., Becker C., Choisne N., Schijlen E., Henri V.D.G., Bianco L., Micheletti D., Velasco R. (2017). High-quality de novo assembly of the apple genome and methylome dynamics of early fruit development. Nat. Genet..

[59] Zhang L.Y., Hu J., Han X.L., Li J.J., Gao Y., Richards C.M., Zhang C.X., Tian Y., Liu G.M., Gul H. (2019). A high-quality apple genome assembly reveals the association of a retrotransposon and red fruit colour. Nat. Commun..

[60] Jung S., Lee T., Cheng C.H., Buble K., Zheng P., Yu J., Humann J., Ficklin S.P., Gasic K., Scott K. (2019). 15 years of GDR: New data and functionality in the Genome Database for Rosaceae. Nucleic Acids Res..

[61] Jin J., Lu P., Xu Y., Li Z., Yu S., Liu J., Wang H., Chua N.H., Cao P. (2021). PLncDB V2.0: A comprehensive encyclopedia of plant long noncoding RNAs. Nucleic Acids Res..

[62] Kang Y.J., Yang D.C., Kong L., Hou M., Meng Y.Q., Wei L., Gao G. (2017). CPC2: A fast and accurate coding potential calculator based on sequence intrinsic features. Nucleic Acids Res..

[63] Biłas R., Szafran K., Hnatuszko-Konka K., Kononowicz A.K. (2016). *Cis*-regulatory elements used to control gene expression in plants. Plant Cell Tissue Organ Cult..

[64] Li Y., Chen C.Y., Kaye A.M., Wasserman W.W. (2015). The identification of *cis*-regulatory elements: A review from a machine learning perspective. BioSystems.

[65] Ye Z.B., Ye J., Wang X., Hu T.X., Zhang F.X., Wang B., Li C.X., Yang T.X., Li H.X., Lu Y.E. (2017). An InDel in the promoter of *Al-activated malate transporter9* selected during tomato domestication determines fruit malate content and aluminum tolerance. Plant Cell.

[66] Zhao L., Li M.M., Xu C.J., Yang X., Li D.M., Zhao X., Wang K., Li Y.H., Zhang X.M., Liu L.X. (2018). Natural variation in *GmGBP1* promoter affects photoperiod control of flowering time and maturity in soybean. Plant J..

[67] Cheng X., Yao J.L., Qin M.F., Zhang M.Y., Allan A.C., Wang D.F., Wu J. (2019). *PbrmiR397a* regulates lignification during stone cell development in pear fruit. Plant Biotechnol. J..

[68] Solano R., Stepanova A., Chao Q., Ecker J.R. (1998). Nuclear events in ethylene signaling: A transcriptional cascade mediated by ETHYLENE-INSENSITIVE3 and ETHYLENE-RESPONSE-FACTOR1. Genes Dev..

[69] Kazan K. (2015). Diverse roles of jasmonates and ethylene in abiotic stress tolerance. Trends Plant Sci..

[70] Shi Y., Tian S., Hou L., Huang X., Zhang X., Guo H., Yang S. (2012). Ethylene signaling negatively regulates freezing tolerance by repressing expression of CBF and Type-A ARR genes in *Arabidopsis*. Plant Cell.

[71] Ariel F., Romero-Barrios N., Jégu T., Benhamed M., Crespi M. (2015). Battles and hijacks: Noncoding transcription in plants. Trends Plant Sci..

[72] Dimond A., Fraser P. (2013). Long noncoding RNAs Xist in three dimensions. Science.

[73] Heo J.B., Sung S. (2011). Vernalization-mediated epigenetic silencing by a long intronic noncoding RNA. Science.

[74] Engreitz J.M., Haines J.E., Perez E.M., Munson G., Chen J., Kane M., Mcdonel P.E., Guttman M., Lander E.S. (2016). Local regulation of gene expression by lncRNA promoters, transcription and splicing. Nature.

[75] Kopp F., Mendell J.T. (2018). Functional classification and experimental dissection of long noncoding RNAs. Cell.

[76] Huarte M., Guttman M., Feldser D., Garber M., Koziol M.J., Kenzelmann-Broz D., Khalil A.M., Zuk O., Amit I., Rabani M. (2010). A large intergenic noncoding RNA induced by p53 mediates global gene repression in the p53 response. Cell.

[77] Martens J.A., Laprade L., Winston F. (2004). Intergenic transcription is required to repress the Saccharomyces cerevisiae SER3 gene. Nature.

[78] Bassett A.R., Akhtar A., Barlow D.P., Bird A.P., Brockdorff N., Duboule D., Ephrussi A., Ferguson-Smith A.C., Gingeras T.R., Haerty W. (2014). Considerations when investigating lncRNA function in vivo. eLife.

[79] Kearse M., Moir R., Wilson A., Stones-Havas S., Cheung M., Sturrock S., Buxton S., Cooper A., Markowitz S., Duran C. (2012). Geneious Basic: An integrated and extendable desktop software platform for the organization and analysis of sequence data. Bioinformatics.

[80] Weirauch M.T., Yang A., Albu M., Cote A.G., Montenegro-Montero A., Drewe P., Najafabadi H.S., Lambert S.A., Mann I., Cook K. (2014). Determination and inference of eukaryotic transcription factor sequence specificity. Cell.

[81] Amack S.C., Antunes M.S. (2020). CaMV35S promoter—A plant biology and biotechnology workhorse in the era of synthetic biology. Curr. Plant Biol..

[82] Hellens R.P., Allan A.C., Friel E.N., Bolitho K., Grafton K., Templeton M.D., Karunairetnam S., Gleave A.P., Laing W.A. (2005). Transient expression vectors for functional genomics, quantification of promoter activity and RNA silencing in plants. Plant Methods.

[83] Schaffer R.J., Ellen N.F., Souleyre E.J.F., Bolitho K., Thodey K., Ledger S., Bowen J.H., Ma J.-H., Nain B., Cohen D. (2007). A genomics approach reveals that aroma production in apple is controlled by ethylene predominantly at the final step in each biosynthetic pathway. Plant Physiol..

[84] Vandesompele J., Preter K.D., Pattyn F., Poppe B., Roy N.V., Paepe A.D., Speleman F. (2002). Accurate normalization of real-time quantitative RT-PCR data by geometric averaging of multiple internal control genes. Genome Biol..

[85] Gleave A.P. (1992). A versatile binary vector system with a T-DNA organisational structure conducive to efficient integration of cloned DNA into the plant genome. Plant Mol. Biol..

[86] Sun S.H., Hu C.G., Qi X.J., Chen J.Y., Muhammad A., Lin M., Fang J.B. (2021). The *AaCBF4-AaBAM3.1* module enhances freezing tolerance of kiwifruit (*Actinidia arguta*). Hortic. Res..

[87] Sparkes I.A., Runions J., Kearns A., Hawes C. (2006). Rapid, transient expression of fluorescent fusion proteins in tobacco plants and generation of stably transformed plants. Nat. Protoc..

[88] Chen P.Y., Wang C.K., Soong S.C., To K.Y. (2003). Complete sequence of the binary vector pBI121 and its application in cloning T-DNA insertion from transgenic plants. Mol. Breed..

[89] Horsch R.B., Fry J.E., Wallroth M., Eichholtz D., Rogers S.G., Fraley R.T. (1985). A simple and general method for transferring genes into plants. Science.

[90] Park E., Cho M., Ki C.S. (2009). Correct use of repeated measures analysis of variance. Korean J. Lab. Med..

[91] Hayter A.J. (1986). The maximum familywise error rate of Fisher’s least significant difference test. J. Am. Stat. Assoc..

