# Supplementary material for: Promoter Variation of the Key Apple Fruit Texture Related Gene MdPG1 and the Upstream Regulation Analysis
Source: Plants (Basel). 2023 Mar 26;12(7):1452. doi: 10.3390/plants12071452 (PMC10096972; doi:10.3390/plants12071452)
Supplement: Supplementary file 1 [file plants-12-01452-s001.zip › Supplemental figures.pptx]

## Slide 1
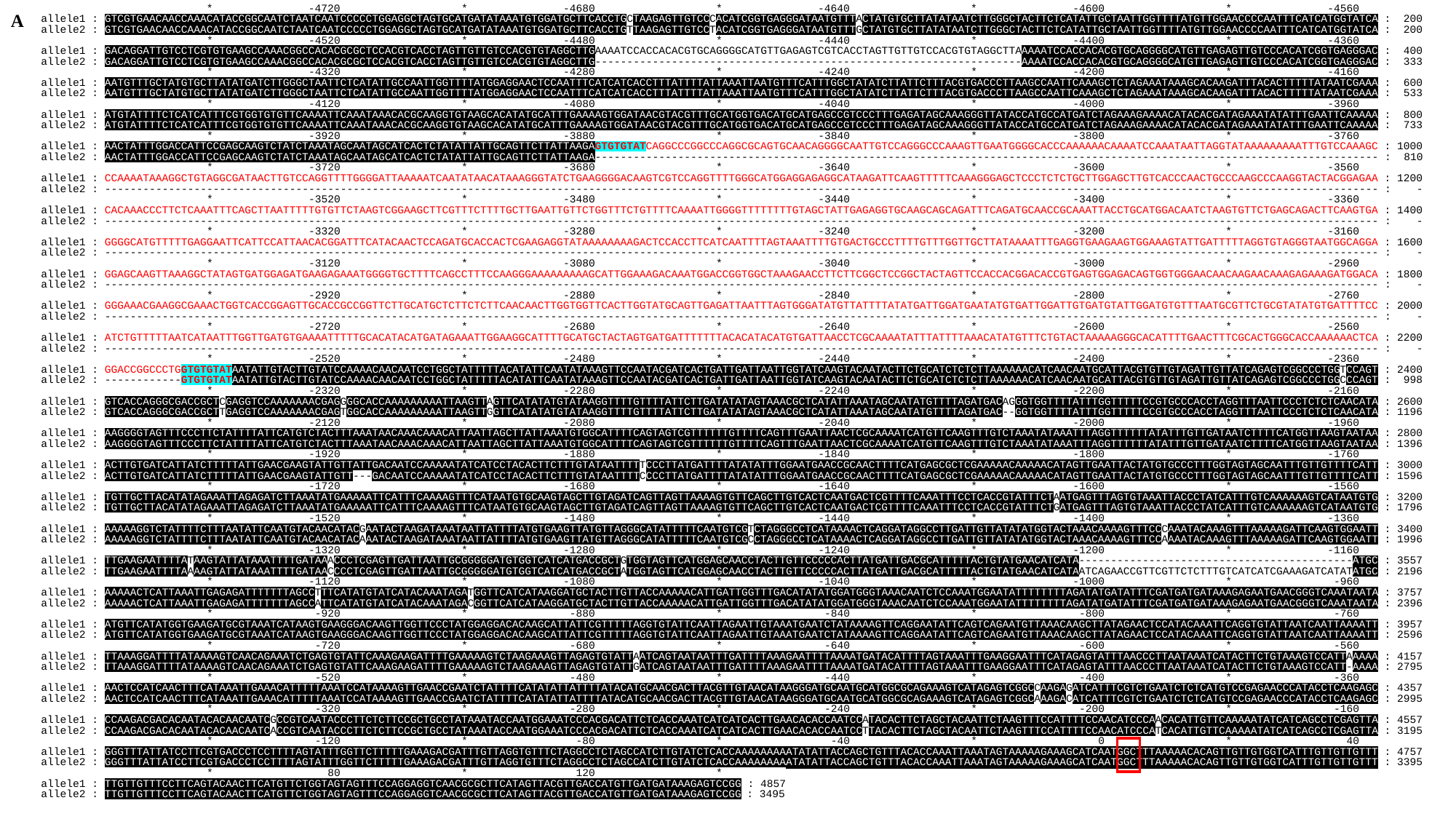

* -4720 * -4680 * -4640 * -4600 * -4560
allele1 : GTCGTGAACAACCAAACATACCGGCAATCTAATCAATCCCCCTGGAGGCTAGTGCATGATATAAATGTGGATGCTTCACCTGCTAAGAGTTGTCCCACATCGGTGAGGGATAATGTTTACTATGTGCTTATATAATCTTGGGCTACTTCTCATATTGCTAATTGGTTTTATGTTGGAACCCCAATTTCATCATGGTATCA : 200
allele2 : GTCGTGAACAACCAAACATACCGGCAATCTAATCAATCCCCCTGGAGGCTAGTGCATGATATAAATGTGGATGCTTCACCTGTTAAGAGTTGTCCTACATCGGTGAGGGATAATGTTTGCTATGTGCTTATATAATCTTGGGCTACTTCTCATATTGCTAATTGGTTTTATGTTGGAACCCCAATTTCATCATGGTATCA : 200
 * -4520 * -4480 * -4440 * -4400 * -4360
allele1 : GACAGGATTGTCCTCGTGTGAAGCCAAACGGCCACACGCGCTCCACGTCACCTAGTTGTTGTCCACGTGTAGGCTTGAAAATCCACCACACGTGCAGGGGCATGTTGAGAGTCGTCACCTAGTTGTTGTCCACGTGTAGGCTTAAAAATCCACCACACGTGCAGGGGCATGTTGAGAGTTGTCCCACATCGGTGAGGGAC : 400
allele2 : GACAGGATTGTCCTCGTGTGAAGCCAAACGGCCACACGCGCTCCACGTCACCTAGTTGTTGTCCACGTGTAGGCTTG-------------------------------------------------------------------AAAATCCACCACACGTGCAGGGGCATGTTGAGAGTTGTCCCACATCGGTGAGGGAC : 333
 * -4320 * -4280 * -4240 * -4200 * -4160
allele1 : AATGTTTGCTATGTGCTTATATGATCTTGGGCTAATTCTCATATTGCCAATTGGTTTTATGGAGGAACTCCAATTTCATCATCACCTTTATTTTATTAAATTAATGTTTCATTTGGCTATATCTTATTCTTTACGTGACCCTTAAGCCAATTCAAAGCTCTAGAAATAAAGCACAAGATTTACACTTTTTATAATCGAAA : 600
allele2 : AATGTTTGCTATGTGCTTATATGATCTTGGGCTAATTCTCATATTGCCAATTGGTTTTATGGAGGAACTCCAATTTCATCATCACCTTTATTTTATTAAATTAATGTTTCATTTGGCTATATCTTATTCTTTACGTGACCCTTAAGCCAATTCAAAGCTCTAGAAATAAAGCACAAGATTTACACTTTTTATAATCGAAA : 533
 * -4120 * -4080 * -4040 * -4000 * -3960
allele1 : ATGTATTTTCTCATCATTTCGTGGTGTGTTCAAAATTCAAATAAACACGCAAGGTGTAAGCACATATGCATTTGAAAAGTGGATAACGTACGTTTGCATGGTGACATGCATGAGCCGTCCCTTTGAGATAGCAAAGGGTTATACCATGCCATGATCTAGAAAGAAAACATACACGATAGAAATATATTTGAATTCAAAAA : 800
allele2 : ATGTATTTTCTCATCATTTCGTGGTGTGTTCAAAATTCAAATAAACACGCAAGGTGTAAGCACATATGCATTTGAAAAGTGGATAACGTACGTTTGCATGGTGACATGCATGAGCCGTCCCTTTGAGATAGCAAAGGGTTATACCATGCCATGATCTAGAAAGAAAACATACACGATAGAAATATATTTGAATTCAAAAA : 733
 * -3920 * -3880 * -3840 * -3800 * -3760
allele1 : AACTATTTGGACCATTCCGAGCAAGTCTATCTAAATAGCAATAGCATCACTCTATATTATTGCAGTTCTTATTAAGAGTGTGTATCAGGCCCGGCCCAGGCGCAGTGCAACAGGGGCAATTGTCCAGGGCCCAAAGTTGAATGGGGCACCCAAAAAACAAAATCCAAATAATTAGGTATAAAAAAAAATTTGTCCAAAGC : 1000
allele2 : AACTATTTGGACCATTCCGAGCAAGTCTATCTAAATAGCAATAGCATCACTCTATATTATTGCAGTTCTTATTAAGA--------------------------------------------------------------------------------------------------------------------------- : 810
 * -3720 * -3680 * -3640 * -3600 * -3560
allele1 : CCAAAATAAAGGCTGTAGGCGATAACTTGTCCAGGTTTTGGGGATTAAAAATCAATATAACATAAAGGGTATCTGAAGGGGACAAGTCGTCCAGGTTTTGGGCATGGAGGAGAGGCATAAGATTCAAGTTTTTCAAAGGGAGCTCCCTCTCTGCTTGGAGCTTGTCACCCAACTGCCCAAGCCCAAGGTACTACGGAGAA : 1200
allele2 : -------------------------------------------------------------------------------------------------------------------------------------------------------------------------------------------------------- : -
 * -3520 * -3480 * -3440 * -3400 * -3360
allele1 : CACAAACCCTTCTCAAATTTCAGCTTAATTTTTGTGTTCTAAGTCGGAAGCTTCGTTTCTTTTGCTTGAATTGTTCTGGTTTCTGTTTTCAAAATTGGGGTTTTTTTTGTAGCTATTGAGAGGTGCAAGCAGCAGATTTCAGATGCAACCGCAAATTACCTGCATGGACAATCTAAGTGTTCTGAGCAGACTTCAAGTGA : 1400
allele2 : -------------------------------------------------------------------------------------------------------------------------------------------------------------------------------------------------------- : -
 * -3320 * -3280 * -3240 * -3200 * -3160
allele1 : GGGGCATGTTTTTGAGGAATTCATTCCATTAACACGGATTTCATACAACTCCAGATGCACCACTCGAAGAGGTATAAAAAAAAGACTCCACCTTCATCAATTTTAGTAAATTTTGTGACTGCCCTTTTGTTTGGTTGCTTATAAAATTTGAGGTGAAGAAGTGGAAAGTATTGATTTTTAGGTGTAGGGTAATGGCAGGA : 1600
allele2 : -------------------------------------------------------------------------------------------------------------------------------------------------------------------------------------------------------- : -
 * -3120 * -3080 * -3040 * -3000 * -2960
allele1 : GGAGCAAGTTAAAGGCTATAGTGATGGAGATGAAGAGAAATGGGGTGCTTTTCAGCCTTTCCAAGGGAAAAAAAAAGCATTGGAAAGACAAATGGACCGGTGGCTAAAGAACCTTCTTCGGCTCCGGCTACTAGTTCCACCACGGACACCGTGAGTGGAGACAGTGGTGGGAACAACAAGAACAAAGAGAAAGATGGACA : 1800
allele2 : -------------------------------------------------------------------------------------------------------------------------------------------------------------------------------------------------------- : -
 * -2920 * -2880 * -2840 * -2800 * -2760
allele1 : GGGAAACGAAGGCGAAACTGGTCACCGGAGTTGCACCGCCGGTTCTTGCATGCTCTTCTCTTCAACAACTTGGTGGTTCACTTGGTATGCAGTTGAGATTAATTTAGTGGGATATGTTATTTTATATGATTGGATGAATATGTGATTGGATTGTGATGTATTGGATGTGTTTAATGCGTTCTGCGTATATGTGATTTTCC : 2000
allele2 : -------------------------------------------------------------------------------------------------------------------------------------------------------------------------------------------------------- : -
 * -2720 * -2680 * -2640 * -2600 * -2560
allele1 : ATCTGTTTTTAATCATAATTTGGTTGATGTGAAAATTTTTGCACATACATGATAGAAATTGGAAGGCATTTTGCATGCTACTAGTGATGATTTTTTTACACATACATGTGATTAACCTCGCAAAATATTTATTTTAAACATATGTTTCTGTACTAAAAAGGGCACATTTTGAACTTTCGCACTGGGCACCAAAAAACTCA : 2200
allele2 : -------------------------------------------------------------------------------------------------------------------------------------------------------------------------------------------------------- : -
 * -2520 * -2480 * -2440 * -2400 * -2360
allele1 : GGACCGGCCCTGGTGTGTATAATATTGTACTTGTATCCAAAACAACAATCCTGGCTATTTTTACATATTCAATATAAAGTTCCAATACGATCACTGATTGATTAATTGGTATCAAGTACAATACTTCTGCATCTCTCTTAAAAAACATCAACAATGCATTACGTGTTGTAGATTGTTATCAGAGTCGGCCCTGGTCCAGT : 2400
allele2 : ------------GTGTGTATAATATTGTACTTGTATCCAAAACAACAATCCTGGCTATTTTTACATATTCAATATAAAGTTCCAATACGATCACTGATTGATTAATTGGTATCAAGTACAATACTTCTGCATCTCTCTTAAAAAACATCAACAATGCATTACGTGTTGTAGATTGTTATCAGAGTCGGCCCTGGCCCAGT : 998
 * -2320 * -2280 * -2240 * -2200 * -2160
allele1 : GTCACCAGGGCGACCGCTCGAGGTCCAAAAAAACGAGGGGCACCAAAAAAAAATTAAGTTAGTTCATATATGTATAAGGTTTTGTTTTATTCTTGATATATAGTAAACGCTCATATTAAATAGCAATATGTTTTAGATGACAGGGTGGTTTTATTTGGTTTTTCCGTGCCCACCTAGGTTTAATTCCCTCTCTCAACATA : 2600
allele2 : GTCACCAGGGCGACCGCTTGAGGTCCAAAAAAACGAGTGGCACCAAAAAAAAATTAAGTTGGTTCATATATGTATAAGGTTTTGTTTTATTCTTGATATATAGTAAACGCTCATATTAAATAGCAATATGTTTTAGATGAC--GGTGGTTTTATTTGGTTTTTCCGTGCCCACCTAGGTTTAATTCCCTCTCTCAACATA : 1196
 * -2120 * -2080 * -2040 * -2000 * -1960
allele1 : AAGGGGTAGTTTCCCTTCTATTTTATTCATGTCTACTTTAAATAACAAACAAACATTAATTAGCTTATTAAATGTGGCATTTTCAGTAGTCGTTTTTTGTTTTCAGTTTGAATTAACTCGCAAAATCATGTTCAAGTTTGTCTAAATATAAATTTAGGTTTTTTATATTTGTTGATAATCTTTTCATGGTTAAGTAATAA : 2800
allele2 : AAGGGGTAGTTTCCCTTCTATTTTATTCATGTCTACTTTAAATAACAAACAAACATTAATTAGCTTATTAAATGTGGCATTTTCAGTAGTCGTTTTTTGTTTTCAGTTTGAATTAACTCGCAAAATCATGTTCAAGTTTGTCTAAATATAAATTTAGGTTTTTTATATTTGTTGATAATCTTTTCATGGTTAAGTAATAA : 1396
 * -1920 * -1880 * -1840 * -1800 * -1760
allele1 : ACTTGTGATCATTATCTTTTTATTGAACGAAGTATTGTTATTGACAATCCAAAAATATCATCCTACACTTCTTTGTATAATTTTTCCCTTATGATTTTATATATTTGGAATGAACCGCAACTTTTCATGAGCGCTCGAAAAACAAAAACATAGTTGAATTACTATGTGCCCTTTGGTAGTAGCAATTTGTTGTTTTCATT : 3000
allele2 : ACTTGTGATCATTATCTTTTTATTGAACGAAGTATTGTT---GACAATCCAAAAATATCATCCTACACTTCTTTGTATAATTTTCCCCTTATGATTTTATATATTTGGAATGAACCGCAACTTTTCATGAGCGCTCGAAAAACAAAAACATAGTTGAATTACTATGTGCCCTTTGGTAGTAGCAATTTGTTGTTTTCATT : 1596
 * -1720 * -1680 * -1640 * -1600 * -1560
allele1 : TGTTGCTTACATATAGAAATTAGAGATCTTAAATATGAAAAATTCATTTCAAAAGTTTCATAATGTGCAAGTAGCTTGTAGATCAGTTAGTTAAAAGTGTTCAGCTTGTCACTCAATGACTCGTTTTCAAATTTCCTCACCGTATTTCTAATGAGTTTAGTGTAAATTACCCTATCATTTGTCAAAAAAGTCATAATGTG : 3200
allele2 : TGTTGCTTACATATAGAAATTAGAGATCTTAAATATGAAAAATTCATTTCAAAAGTTTCATAATGTGCAAGTAGCTTGTAGATCAGTTAGTTAAAAGTGTTCAGCTTGTCACTCAATGACTCGTTTTCAAATTTCCTCACCGTATTTCTGATGAGTTTAGTGTAAATTACCCTATCATTTGTCAAAAAAGTCATAATGTG : 1796
 * -1520 * -1480 * -1440 * -1400 * -1360
allele1 : AAAAAGGTCTATTTTCTTTAATATTCAATGTACAACATACGAATACTAAGATAAATAATTATTTTATGTGAAGTTATGTTAGGGCATATTTTTCAATGTCGTCTAGGGCCTCATAAAACTCAGGATAGGCCTTGATTGTTATATATGGTACTAAACAAAAGTTTCCCAAATACAAAGTTTAAAAAGATTCAAGTGGAATT : 3400
allele2 : AAAAAGGTCTATTTTCTTTAATATTCAATGTACAACATACAAATACTAAGATAAATAATTATTTTATGTGAAGTTATGTTAGGGCATATTTTTCAATGTCGCCTAGGGCCTCATAAAACTCAGGATAGGCCTTGATTGTTATATATGGTACTAAACAAAAGTTTCCAAAATACAAAGTTTAAAAAGATTCAAGTGGAATT : 1996
 * -1320 * -1280 * -1240 * -1200 * -1160
allele1 : TTGAAGAATTTTATAAGTATTATAAATTTTGATAAACCCTCGAGTTGATTAATTGCGGGGGATGTGGTCATCATGACCGCTGTGGTAGTTCATGGAGCAACCTACTTGTTCCCCCACTTATGATTGACGCATTTTTACTGTATGAACATCATA-------------------------------------------ATGC : 3557
allele2 : TTGAAGAATTTTAAAAGTATTATAAATTTTGATAACCCCTCGAGTTGATTAATTGCGGGGGATGTGGTCATCATGACCGCTATGGTAGTTCATGGAGCAACCTACTTGTTCCCCCACTTATGATTGACGCATTTTTACTGTATGAACATCATAATCAGAACCGTTCGTTCTCTTTGTCATCATCGAAAGATCATATATGC : 2196
 * -1120 * -1080 * -1040 * -1000 * -960
allele1 : AAAAACTCATTAAATTGAGAGATTTTTTTAGCCTTTCATATGTATCATACAAATAGATGGTTCATCATAAGGATGCTACTTGTTACCAAAAACATTGATTGGTTTGACATATATGGATGGGTAAACAATCTCCAAATGGAATATTTTTTTTAGATATGATATTTCGATGATGATAAAGAGAATGAACGGGTCAAATAATA : 3757
allele2 : AAAAACTCATTAAATTGAGAGATTTTTTTAGCCATTCATATGTATCATACAAATAGACGGTTCATCATAAGGATGCTACTTGTTACCAAAAACATTGATTGGTTTGACATATATGGATGGGTAAACAATCTCCAAATGGAATATTTTTTTTAGATATGATATTTCGATGATGATAAAGAGAATGAACGGGTCAAATAATA : 2396
 * -920 * -880 * -840 * -800 * -760
allele1 : ATGTTCATATGGTGAAGATGCGTAAATCATAAGTGAAGGGACAAGTTGGTTCCCTATGGAGGACACAAGCATTATTCGTTTTTAGGTGTATTCAATTAGAATTGTAAATGAATCTATAAAAGTTCAGGAATATTCAGTCAGAATGTTAAACAAGCTTATAGAACTCCATACAAATTCAGGTGTATTAATCAATTAAAATT : 3957
allele2 : ATGTTCATATGGTGAAGATGCGTAAATCATAAGTGAAGGGACAAGTTGGTTCCCTATGGAGGACACAAGCATTATTCGTTTTTAGGTGTATTCAATTAGAATTGTAAATGAATCTATAAAAGTTCAGGAATATTCAGTCAGAATGTTAAACAAGCTTATAGAACTCCATACAAATTCAGGTGTATTAATCAATTAAAATT : 2596
 * -720 * -680 * -640 * -600 * -560
allele1 : TTAAAGGATTTTATAAAAGTCAACAGAAATCTGAGTGTATTCAAAGAAGATTTTGAAAAAGTCTAAGAAAGTTAGAGTGTATTAATCAGTAATAATTTGATTTTAAAGAATTTTAAAATGATACATTTTAGTAAATTTGAAGGAATTTCATAGAGTATTTAACCCTTAATAAATCATACTTCTGTAAAGTCCATTAAAAA : 4157
allele2 : TTAAAGGATTTTATAAAAGTCAACAGAAATCTGAGTGTATTCAAAGAAGATTTTGAAAAAGTCTAAGAAAGTTAGAGTGTATTGATCAGTAATAATTTGATTTTAAAGAATTTTAAAATGATACATTTTAGTAAATTTGAAGGAATTTCATAGAGTATTTAACCCTTAATAAATCATACTTCTGTAAAGTCCATT-AAAA : 2795
 * -520 * -480 * -440 * -400 * -360
allele1 : AACTCCATCAACTTTCATAAATTGAAACATTTTTAAATCCATAAAAGTTGAACCGAATCTATTTTCATATATTATTTTATACATGCAACGACTTACGTTGTAACATAAGGGATGCAATGCATGGCGCAGAAAGTCATAGAGTCGGCCAAGAGATCATTTCGTCTGAATCTCTCATGTCCGAGAACCCATACCTCAAGAGC : 4357
allele2 : AACTCCATCAACTTTCATAAATTGAAACATTTTTAAATCCATAAAAGTTGAACCGAATCTATTTTCATATATTATTTTATACATGCAACGACTTACGTTGTAACATAAGGGATGCAATGCATGGCGCAGAAAGTCATAGAGTCGGCAAAGACATCATTTCGTCTGAATCTCTCATGTCCGAGAACCCATACCTCAAGAGC : 2995
 * -320 * -280 * -240 * -200 * -160
allele1 : CCAAGACGACACAATACACAACAATCGCCGTCAATACCCTTCTCTTCCGCTGCCTATAAATACCAATGGAAATCCCACGACATTCTCACCAAATCATCATCACTTGAACACACCAATCCATACACTTCTAGCTACAATTCTAAGTTTCCATTTTCCAACATCCCAACACATTGTTCAAAAATATCATCAGCCTCGAGTTA : 4557
allele2 : CCAAGACGACACAATACACAACAATCACCGTCAATACCCTTCTCTTCCGCTGCCTATAAATACCAATGGAAATCCCACGACATTCTCACCAAATCATCATCACTTGAACACACCAATCCTTACACTTCTAGCTACAATTCTAAGTTTCCATTTTCCAACATCCCATCACATTGTTCAAAAATATCATCAGCCTCGAGTTA : 3195
 * -120 * -80 * -40 * 0 * 40
allele1 : GGGTTTATTATCCTTCGTGACCCTCCTTTTAGTATTTGGTTCTTTTTGAAAGACGATTTGTTAGGTGTTTCTAGGCCTCTAGCCATCTTGTATCTCACCAAAAAAAAATATATTACCAGCTGTTTACACCAAATTAAATAGTAAAAAGAAAGCATCAATGGCTTTAAAAACACAGTTGTTGTGGTCATTTGTTGTTGTTT : 4757
allele2 : GGGTTTATTATCCTTCGTGACCCTCCTTTTAGTATTTGGTTCTTTTTGAAAGACGATTTGTTAGGTGTTTCTAGGCCTCTAGCCATCTTGTATCTCACCAAAAAAAAATATATTACCAGCTGTTTACACCAAATTAAATAGTAAAAAGAAAGCATCAATGGCTTTAAAAACACAGTTGTTGTGGTCATTTGTTGTTGTTT : 3395
 * 80 * 120 *
allele1 : TTGTTGTTTCCTTCAGTACAACTTCATGTTCTGGTAGTAGTTTCCAGGAGGTCAACGCGCTTCATAGTTACGTTGACCATGTTGATGATAAAGAGTCCGG : 4857
allele2 : TTGTTGTTTCCTTCAGTACAACTTCATGTTCTGGTAGTAGTTTCCAGGAGGTCAACGCGCTTCATAGTTACGTTGACCATGTTGATGATAAAGAGTCCGG : 3495
A

## Slide 2
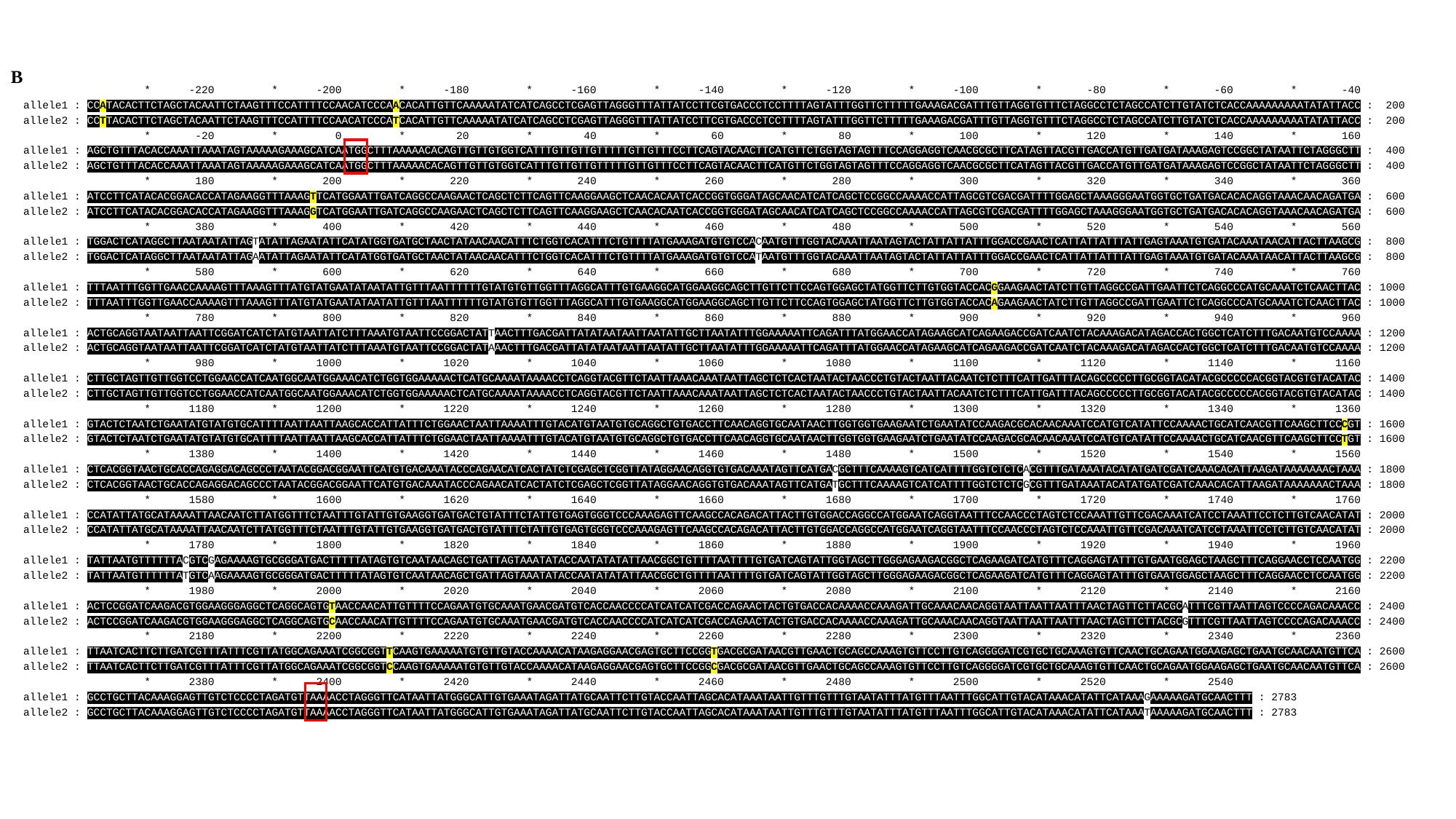

B
 * -220 * -200 * -180 * -160 * -140 * -120 * -100 * -80 * -60 * -40
allele1 : CCATACACTTCTAGCTACAATTCTAAGTTTCCATTTTCCAACATCCCAACACATTGTTCAAAAATATCATCAGCCTCGAGTTAGGGTTTATTATCCTTCGTGACCCTCCTTTTAGTATTTGGTTCTTTTTGAAAGACGATTTGTTAGGTGTTTCTAGGCCTCTAGCCATCTTGTATCTCACCAAAAAAAAATATATTACC : 200
allele2 : CCTTACACTTCTAGCTACAATTCTAAGTTTCCATTTTCCAACATCCCATCACATTGTTCAAAAATATCATCAGCCTCGAGTTAGGGTTTATTATCCTTCGTGACCCTCCTTTTAGTATTTGGTTCTTTTTGAAAGACGATTTGTTAGGTGTTTCTAGGCCTCTAGCCATCTTGTATCTCACCAAAAAAAAATATATTACC : 200
 * -20 * 0 * 20 * 40 * 60 * 80 * 100 * 120 * 140 * 160
allele1 : AGCTGTTTACACCAAATTAAATAGTAAAAAGAAAGCATCAATGGCTTTAAAAACACAGTTGTTGTGGTCATTTGTTGTTGTTTTTGTTGTTTCCTTCAGTACAACTTCATGTTCTGGTAGTAGTTTCCAGGAGGTCAACGCGCTTCATAGTTACGTTGACCATGTTGATGATAAAGAGTCCGGCTATAATTCTAGGGCTT : 400
allele2 : AGCTGTTTACACCAAATTAAATAGTAAAAAGAAAGCATCAATGGCTTTAAAAACACAGTTGTTGTGGTCATTTGTTGTTGTTTTTGTTGTTTCCTTCAGTACAACTTCATGTTCTGGTAGTAGTTTCCAGGAGGTCAACGCGCTTCATAGTTACGTTGACCATGTTGATGATAAAGAGTCCGGCTATAATTCTAGGGCTT : 400
 * 180 * 200 * 220 * 240 * 260 * 280 * 300 * 320 * 340 * 360
allele1 : ATCCTTCATACACGGACACCATAGAAGGTTTAAAGTTCATGGAATTGATCAGGCCAAGAACTCAGCTCTTCAGTTCAAGGAAGCTCAACACAATCACCGGTGGGATAGCAACATCATCAGCTCCGGCCAAAACCATTAGCGTCGACGATTTTGGAGCTAAAGGGAATGGTGCTGATGACACACAGGTAAACAACAGATGA : 600
allele2 : ATCCTTCATACACGGACACCATAGAAGGTTTAAAGGTCATGGAATTGATCAGGCCAAGAACTCAGCTCTTCAGTTCAAGGAAGCTCAACACAATCACCGGTGGGATAGCAACATCATCAGCTCCGGCCAAAACCATTAGCGTCGACGATTTTGGAGCTAAAGGGAATGGTGCTGATGACACACAGGTAAACAACAGATGA : 600
 * 380 * 400 * 420 * 440 * 460 * 480 * 500 * 520 * 540 * 560
allele1 : TGGACTCATAGGCTTAATAATATTAGTATATTAGAATATTCATATGGTGATGCTAACTATAACAACATTTCTGGTCACATTTCTGTTTTATGAAAGATGTGTCCACAATGTTTGGTACAAATTAATAGTACTATTATTATTTGGACCGAACTCATTATTATTTATTGAGTAAATGTGATACAAATAACATTACTTAAGCG : 800
allele2 : TGGACTCATAGGCTTAATAATATTAGAATATTAGAATATTCATATGGTGATGCTAACTATAACAACATTTCTGGTCACATTTCTGTTTTATGAAAGATGTGTCCATAATGTTTGGTACAAATTAATAGTACTATTATTATTTGGACCGAACTCATTATTATTTATTGAGTAAATGTGATACAAATAACATTACTTAAGCG : 800
 * 580 * 600 * 620 * 640 * 660 * 680 * 700 * 720 * 740 * 760
allele1 : TTTAATTTGGTTGAACCAAAAGTTTAAAGTTTATGTATGAATATAATATTGTTTAATTTTTTGTATGTGTTGGTTTAGGCATTTGTGAAGGCATGGAAGGCAGCTTGTTCTTCCAGTGGAGCTATGGTTCTTGTGGTACCACGGAAGAACTATCTTGTTAGGCCGATTGAATTCTCAGGCCCATGCAAATCTCAACTTAC : 1000
allele2 : TTTAATTTGGTTGAACCAAAAGTTTAAAGTTTATGTATGAATATAATATTGTTTAATTTTTTGTATGTGTTGGTTTAGGCATTTGTGAAGGCATGGAAGGCAGCTTGTTCTTCCAGTGGAGCTATGGTTCTTGTGGTACCACAGAAGAACTATCTTGTTAGGCCGATTGAATTCTCAGGCCCATGCAAATCTCAACTTAC : 1000
 * 780 * 800 * 820 * 840 * 860 * 880 * 900 * 920 * 940 * 960
allele1 : ACTGCAGGTAATAATTAATTCGGATCATCTATGTAATTATCTTTAAATGTAATTCCGGACTATTAACTTTGACGATTATATAATAATTAATATTGCTTAATATTTGGAAAAATTCAGATTTATGGAACCATAGAAGCATCAGAAGACCGATCAATCTACAAAGACATAGACCACTGGCTCATCTTTGACAATGTCCAAAA : 1200
allele2 : ACTGCAGGTAATAATTAATTCGGATCATCTATGTAATTATCTTTAAATGTAATTCCGGACTATAAACTTTGACGATTATATAATAATTAATATTGCTTAATATTTGGAAAAATTCAGATTTATGGAACCATAGAAGCATCAGAAGACCGATCAATCTACAAAGACATAGACCACTGGCTCATCTTTGACAATGTCCAAAA : 1200
 * 980 * 1000 * 1020 * 1040 * 1060 * 1080 * 1100 * 1120 * 1140 * 1160
allele1 : CTTGCTAGTTGTTGGTCCTGGAACCATCAATGGCAATGGAAACATCTGGTGGAAAAACTCATGCAAAATAAAACCTCAGGTACGTTCTAATTAAACAAATAATTAGCTCTCACTAATACTAACCCTGTACTAATTACAATCTCTTTCATTGATTTACAGCCCCCTTGCGGTACATACGCCCCCACGGTACGTGTACATAC : 1400
allele2 : CTTGCTAGTTGTTGGTCCTGGAACCATCAATGGCAATGGAAACATCTGGTGGAAAAACTCATGCAAAATAAAACCTCAGGTACGTTCTAATTAAACAAATAATTAGCTCTCACTAATACTAACCCTGTACTAATTACAATCTCTTTCATTGATTTACAGCCCCCTTGCGGTACATACGCCCCCACGGTACGTGTACATAC : 1400
 * 1180 * 1200 * 1220 * 1240 * 1260 * 1280 * 1300 * 1320 * 1340 * 1360
allele1 : GTACTCTAATCTGAATATGTATGTGCATTTTAATTAATTAAGCACCATTATTTCTGGAACTAATTAAAATTTGTACATGTAATGTGCAGGCTGTGACCTTCAACAGGTGCAATAACTTGGTGGTGAAGAATCTGAATATCCAAGACGCACAACAAATCCATGTCATATTCCAAAACTGCATCAACGTTCAAGCTTCCCGT : 1600
allele2 : GTACTCTAATCTGAATATGTATGTGCATTTTAATTAATTAAGCACCATTATTTCTGGAACTAATTAAAATTTGTACATGTAATGTGCAGGCTGTGACCTTCAACAGGTGCAATAACTTGGTGGTGAAGAATCTGAATATCCAAGACGCACAACAAATCCATGTCATATTCCAAAACTGCATCAACGTTCAAGCTTCCTGT : 1600
 * 1380 * 1400 * 1420 * 1440 * 1460 * 1480 * 1500 * 1520 * 1540 * 1560
allele1 : CTCACGGTAACTGCACCAGAGGACAGCCCTAATACGGACGGAATTCATGTGACAAATACCCAGAACATCACTATCTCGAGCTCGGTTATAGGAACAGGTGTGACAAATAGTTCATGACGCTTTCAAAAGTCATCATTTTGGTCTCTCACGTTTGATAAATACATATGATCGATCAAACACATTAAGATAAAAAAACTAAA : 1800
allele2 : CTCACGGTAACTGCACCAGAGGACAGCCCTAATACGGACGGAATTCATGTGACAAATACCCAGAACATCACTATCTCGAGCTCGGTTATAGGAACAGGTGTGACAAATAGTTCATGATGCTTTCAAAAGTCATCATTTTGGTCTCTCGCGTTTGATAAATACATATGATCGATCAAACACATTAAGATAAAAAAACTAAA : 1800
 * 1580 * 1600 * 1620 * 1640 * 1660 * 1680 * 1700 * 1720 * 1740 * 1760
allele1 : CCATATTATGCATAAAATTAACAATCTTATGGTTTCTAATTTGTATTGTGAAGGTGATGACTGTATTTCTATTGTGAGTGGGTCCCAAAGAGTTCAAGCCACAGACATTACTTGTGGACCAGGCCATGGAATCAGGTAATTTCCAACCCTAGTCTCCAAATTGTTCGACAAATCATCCTAAATTCCTCTTGTCAACATAT : 2000
allele2 : CCATATTATGCATAAAATTAACAATCTTATGGTTTCTAATTTGTATTGTGAAGGTGATGACTGTATTTCTATTGTGAGTGGGTCCCAAAGAGTTCAAGCCACAGACATTACTTGTGGACCAGGCCATGGAATCAGGTAATTTCCAACCCTAGTCTCCAAATTGTTCGACAAATCATCCTAAATTCCTCTTGTCAACATAT : 2000
 * 1780 * 1800 * 1820 * 1840 * 1860 * 1880 * 1900 * 1920 * 1940 * 1960
allele1 : TATTAATGTTTTTTACGTCGAGAAAAGTGCGGGATGACTTTTTATAGTGTCAATAACAGCTGATTAGTAAATATACCAATATATATTAACGGCTGTTTTAATTTTGTGATCAGTATTGGTAGCTTGGGAGAAGACGGCTCAGAAGATCATGTTTCAGGAGTATTTGTGAATGGAGCTAAGCTTTCAGGAACCTCCAATGG : 2200
allele2 : TATTAATGTTTTTTATGTCAAGAAAAGTGCGGGATGACTTTTTATAGTGTCAATAACAGCTGATTAGTAAATATACCAATATATATTAACGGCTGTTTTAATTTTGTGATCAGTATTGGTAGCTTGGGAGAAGACGGCTCAGAAGATCATGTTTCAGGAGTATTTGTGAATGGAGCTAAGCTTTCAGGAACCTCCAATGG : 2200
 * 1980 * 2000 * 2020 * 2040 * 2060 * 2080 * 2100 * 2120 * 2140 * 2160
allele1 : ACTCCGGATCAAGACGTGGAAGGGAGGCTCAGGCAGTGTAACCAACATTGTTTTCCAGAATGTGCAAATGAACGATGTCACCAACCCCATCATCATCGACCAGAACTACTGTGACCACAAAACCAAAGATTGCAAACAACAGGTAATTAATTAATTTAACTAGTTCTTACGCATTTCGTTAATTAGTCCCCAGACAAACC : 2400
allele2 : ACTCCGGATCAAGACGTGGAAGGGAGGCTCAGGCAGTGCAACCAACATTGTTTTCCAGAATGTGCAAATGAACGATGTCACCAACCCCATCATCATCGACCAGAACTACTGTGACCACAAAACCAAAGATTGCAAACAACAGGTAATTAATTAATTTAACTAGTTCTTACGCGTTTCGTTAATTAGTCCCCAGACAAACC : 2400
 * 2180 * 2200 * 2220 * 2240 * 2260 * 2280 * 2300 * 2320 * 2340 * 2360
allele1 : TTAATCACTTCTTGATCGTTTATTTCGTTATGGCAGAAATCGGCGGTTCAAGTGAAAAATGTGTTGTACCAAAACATAAGAGGAACGAGTGCTTCCGGTGACGCGATAACGTTGAACTGCAGCCAAAGTGTTCCTTGTCAGGGGATCGTGCTGCAAAGTGTTCAACTGCAGAATGGAAGAGCTGAATGCAACAATGTTCA : 2600
allele2 : TTAATCACTTCTTGATCGTTTATTTCGTTATGGCAGAAATCGGCGGTCCAAGTGAAAAATGTGTTGTACCAAAACATAAGAGGAACGAGTGCTTCCGGCGACGCGATAACGTTGAACTGCAGCCAAAGTGTTCCTTGTCAGGGGATCGTGCTGCAAAGTGTTCAACTGCAGAATGGAAGAGCTGAATGCAACAATGTTCA : 2600
 * 2380 * 2400 * 2420 * 2440 * 2460 * 2480 * 2500 * 2520 * 2540
allele1 : GCCTGCTTACAAAGGAGTTGTCTCCCCTAGATGTTAAAACCTAGGGTTCATAATTATGGGCATTGTGAAATAGATTATGCAATTCTTGTACCAATTAGCACATAAATAATTGTTTGTTTGTAATATTTATGTTTAATTTGGCATTGTACATAAACATATTCATAAAGAAAAAGATGCAACTTT : 2783
allele2 : GCCTGCTTACAAAGGAGTTGTCTCCCCTAGATGTTAAAACCTAGGGTTCATAATTATGGGCATTGTGAAATAGATTATGCAATTCTTGTACCAATTAGCACATAAATAATTGTTTGTTTGTAATATTTATGTTTAATTTGGCATTGTACATAAACATATTCATAAATAAAAAGATGCAACTTT : 2783

## Slide 3
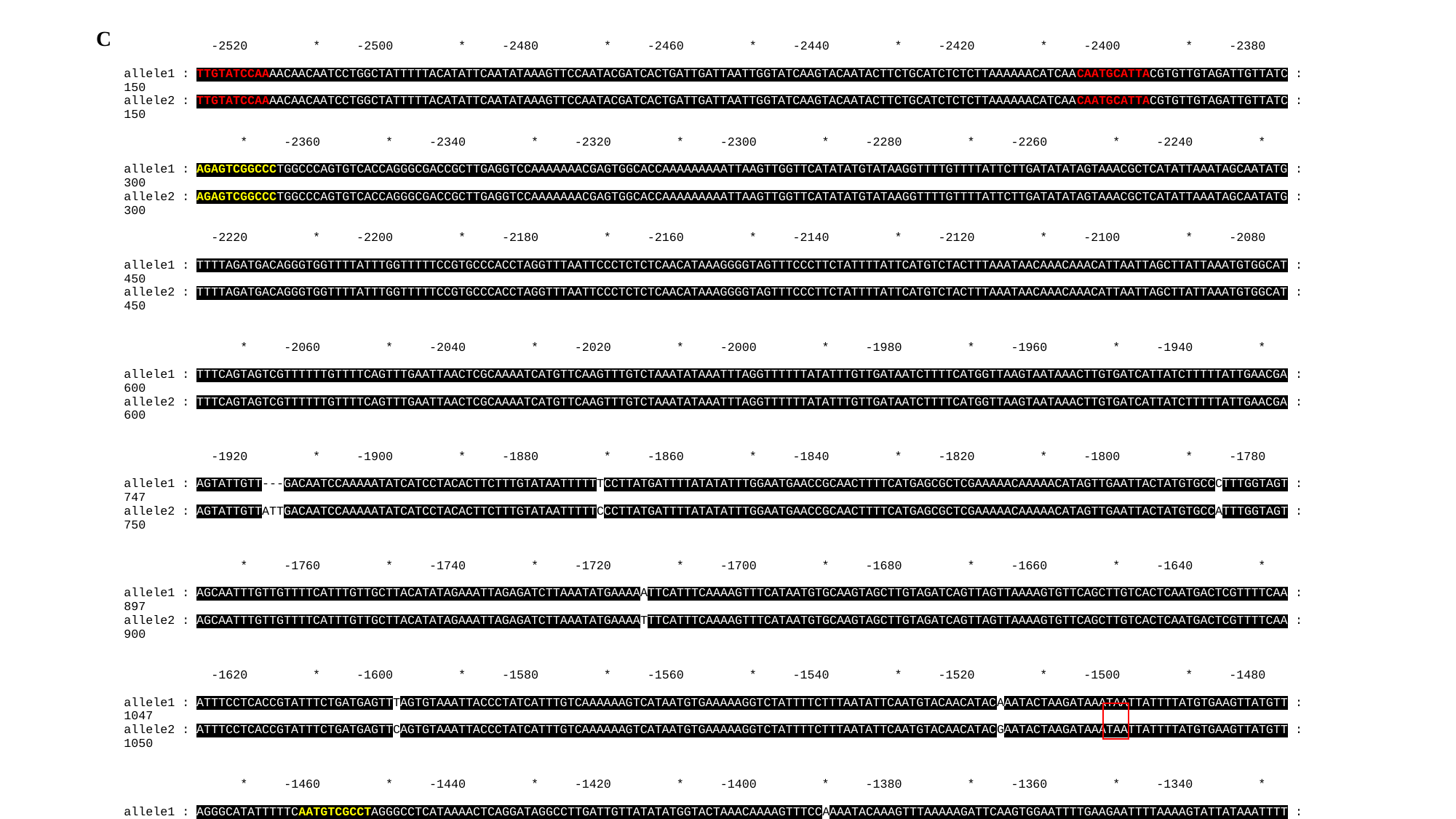

C
 -2520 * -2500 * -2480 * -2460 * -2440 * -2420 * -2400 * -2380
allele1 : TTGTATCCAAAACAACAATCCTGGCTATTTTTACATATTCAATATAAAGTTCCAATACGATCACTGATTGATTAATTGGTATCAAGTACAATACTTCTGCATCTCTCTTAAAAAACATCAACAATGCATTACGTGTTGTAGATTGTTATC : 150
allele2 : TTGTATCCAAAACAACAATCCTGGCTATTTTTACATATTCAATATAAAGTTCCAATACGATCACTGATTGATTAATTGGTATCAAGTACAATACTTCTGCATCTCTCTTAAAAAACATCAACAATGCATTACGTGTTGTAGATTGTTATC : 150
 * -2360 * -2340 * -2320 * -2300 * -2280 * -2260 * -2240 *
allele1 : AGAGTCGGCCCTGGCCCAGTGTCACCAGGGCGACCGCTTGAGGTCCAAAAAAACGAGTGGCACCAAAAAAAAATTAAGTTGGTTCATATATGTATAAGGTTTTGTTTTATTCTTGATATATAGTAAACGCTCATATTAAATAGCAATATG : 300
allele2 : AGAGTCGGCCCTGGCCCAGTGTCACCAGGGCGACCGCTTGAGGTCCAAAAAAACGAGTGGCACCAAAAAAAAATTAAGTTGGTTCATATATGTATAAGGTTTTGTTTTATTCTTGATATATAGTAAACGCTCATATTAAATAGCAATATG : 300
 -2220 * -2200 * -2180 * -2160 * -2140 * -2120 * -2100 * -2080
allele1 : TTTTAGATGACAGGGTGGTTTTATTTGGTTTTTCCGTGCCCACCTAGGTTTAATTCCCTCTCTCAACATAAAGGGGTAGTTTCCCTTCTATTTTATTCATGTCTACTTTAAATAACAAACAAACATTAATTAGCTTATTAAATGTGGCAT : 450
allele2 : TTTTAGATGACAGGGTGGTTTTATTTGGTTTTTCCGTGCCCACCTAGGTTTAATTCCCTCTCTCAACATAAAGGGGTAGTTTCCCTTCTATTTTATTCATGTCTACTTTAAATAACAAACAAACATTAATTAGCTTATTAAATGTGGCAT : 450
 * -2060 * -2040 * -2020 * -2000 * -1980 * -1960 * -1940 *
allele1 : TTTCAGTAGTCGTTTTTTGTTTTCAGTTTGAATTAACTCGCAAAATCATGTTCAAGTTTGTCTAAATATAAATTTAGGTTTTTTATATTTGTTGATAATCTTTTCATGGTTAAGTAATAAACTTGTGATCATTATCTTTTTATTGAACGA : 600
allele2 : TTTCAGTAGTCGTTTTTTGTTTTCAGTTTGAATTAACTCGCAAAATCATGTTCAAGTTTGTCTAAATATAAATTTAGGTTTTTTATATTTGTTGATAATCTTTTCATGGTTAAGTAATAAACTTGTGATCATTATCTTTTTATTGAACGA : 600
 -1920 * -1900 * -1880 * -1860 * -1840 * -1820 * -1800 * -1780
allele1 : AGTATTGTT---GACAATCCAAAAATATCATCCTACACTTCTTTGTATAATTTTTTCCTTATGATTTTATATATTTGGAATGAACCGCAACTTTTCATGAGCGCTCGAAAAACAAAAACATAGTTGAATTACTATGTGCCCTTTGGTAGT : 747
allele2 : AGTATTGTTATTGACAATCCAAAAATATCATCCTACACTTCTTTGTATAATTTTTCCCTTATGATTTTATATATTTGGAATGAACCGCAACTTTTCATGAGCGCTCGAAAAACAAAAACATAGTTGAATTACTATGTGCCATTTGGTAGT : 750
 * -1760 * -1740 * -1720 * -1700 * -1680 * -1660 * -1640 *
allele1 : AGCAATTTGTTGTTTTCATTTGTTGCTTACATATAGAAATTAGAGATCTTAAATATGAAAAATTCATTTCAAAAGTTTCATAATGTGCAAGTAGCTTGTAGATCAGTTAGTTAAAAGTGTTCAGCTTGTCACTCAATGACTCGTTTTCAA : 897
allele2 : AGCAATTTGTTGTTTTCATTTGTTGCTTACATATAGAAATTAGAGATCTTAAATATGAAAATTTCATTTCAAAAGTTTCATAATGTGCAAGTAGCTTGTAGATCAGTTAGTTAAAAGTGTTCAGCTTGTCACTCAATGACTCGTTTTCAA : 900
 -1620 * -1600 * -1580 * -1560 * -1540 * -1520 * -1500 * -1480
allele1 : ATTTCCTCACCGTATTTCTGATGAGTTTAGTGTAAATTACCCTATCATTTGTCAAAAAAGTCATAATGTGAAAAAGGTCTATTTTCTTTAATATTCAATGTACAACATACAAATACTAAGATAAATAATTATTTTATGTGAAGTTATGTT : 1047
allele2 : ATTTCCTCACCGTATTTCTGATGAGTTCAGTGTAAATTACCCTATCATTTGTCAAAAAAGTCATAATGTGAAAAAGGTCTATTTTCTTTAATATTCAATGTACAACATACGAATACTAAGATAAATAATTATTTTATGTGAAGTTATGTT : 1050
 * -1460 * -1440 * -1420 * -1400 * -1380 * -1360 * -1340 *
allele1 : AGGGCATATTTTTCAATGTCGCCTAGGGCCTCATAAAACTCAGGATAGGCCTTGATTGTTATATATGGTACTAAACAAAAGTTTCCAAAATACAAAGTTTAAAAAGATTCAAGTGGAATTTTGAAGAATTTTAAAAGTATTATAAATTTT : 1197
allele2 : AGGGCATATTTTTCAATGTCGCCTAGGGCCTCATAAAACTCAGGATAGGCCTTGATTGTTATATATGGTACTAAACAAAAGTTTCCCAAATACAAAGTTTAAAAAGATTCAAGTGGAATTTTGAAGAATTTTAAAAGTATTATAAATTTT : 1200
 -1320 * -1300 * -1280 * -1260 * -1240 * -1220 * -1200 * -1180
allele1 : GATAACCCCTCGAGTTGATTAATTGCGGGGGATGTGGTCATCATGACCGCTATGGTAGTTCATGGAGCAACCTACTTGTTCCCCCACTTATGATTGACGCATTTTTACTGTATGAACATCATAATCAGAACCGTTCGTTCTCTTTGTCAT : 1347
allele2 : GATAAACCCTCGAGTTGATTAATTGCGGGGGATGTGGTCATCATGACCGCTGTGGTAGTTCATGGAGCAACCTACTTGTTCCCCCACTTATGATTGACGCATTTTTACTATATGAACATCATAATCAGAACCGTTCGTTCTCTTTGTCAT : 1350
 * -1160 * -1140 * -1120 * -1100 * -1080 * -1060 * -1040 *
allele1 : CATCGAAAGATCATATATGCAAAAACTCATTAAATTGAGAGATTTTTTTAGCCATTCATATGTATCATACAAATAGACGGTTCATCATAAGGATGCTACTTGTTACCAAAAACATTGATTGGTTTGACATATATGGATGGGTAAACAATC : 1497
allele2 : CATCGAAAGATCATATATGCAAAAACTCATTAAATTGAGATATTTTTTTAGCCATTCATATGTATCATACAAATAGACGGTTCATCATAAGGATGTTACTTGTTACCAAAAACATTGATTGGTTTGACATATATGGATGGGTAAACAATC : 1500
 -1020 * -1000 * -980 * -960 * -940 * -920 * -900 * -880
allele1 : TCCAAATGGAATATTTTTTTTAGATATGATATTTCGATGATGATAAAGAGAATGAACGGGTCAAATAATAATGTTCATATGGTGAAGATGCGTAAATCATAAGTGAAGGGACAAGTTGGTTCCCTATGGAGGACACAAGCATTATTCGTT : 1647
allele2 : TCCAAATGGAATATTTTTTTTAGATATGATATTTCGATGATGATAAAGAGAATGAACGGGTCAAATAATAATGTTCATATGGTGAAGATGCGTAAATCATAAGTGAAGGGACAAGTTGGTTCCCTATGGAGGACACAAGCATTATTTGTT : 1650
 * -860 * -840 * -820 * -800 * -780 * -760 * -740 *
allele1 : TTTAGGTGTATTCAATTAGAATTGTAAATGAATCTATAAAAGTTCAGGAATATTCAGTCAGAATGTTAAACAAGCTTATAGAACTCCATACAAATTCAGGTGTATTAATCAATTAAAATTTTAAAGGATTTTATAAAAGTCAACAGAAAT : 1797
allele2 : TTTAGGTGTATTCAATTAGAATTGTAAATGAATCTATAAAAGTTCAGGAATATTCAGTCAGAATGTTAAACAAGCTTATAGAACTCCATACAAATTCAGGTGTATTAATCAATTAAAATTTTAAAGGATTTTATAAAAGTCAACAGAAAT : 1800
 -720 * -700 * -680 * -660 * -640 * -620 * -600 * -580
allele1 : CTGAGTGTATTCAAAGAAGATTTTGAAAAAGTCTAAGAAAGTTAGAGTGTATTGATCAGTAATAATTTGATTTTAAAGAATTTTAAAATGATACATTTTAGTAAATTTGAAGGAATTTCATAGAGTATTTAACCCTTAATAAATCATACT : 1947
allele2 : CTGAGTGTATTCAAAGAAGATTTTGAAAAAGTCTAAGAAAGTTAGAGTGTATTAATTAGTAATAATTTGATTTTAAAGAATTTTAAAATGATACATTTTAGTAAATTTGAAGGAATTTCATAGAGTATTTAACCCTTAATAAATCATACT : 1950
 * -560 * -540 * -520 * -500 * -480 * -460 * -440 *
allele1 : TCTGTAAAGTCCATTAAAAAACTCCATCAACTTTCATAAATTGAAACATTTTTAAATCCATAAAAGTTGAACCGAATCTATTTTCATATATTATTTTATACATGCAACGACTTACGTTGTAACATAAGGGATGCAATGCATGGCGCAGAA : 2097
allele2 : TCTGTAAAGTCCATTAAAATACTCCATCAACTTTCATAAATTGAAACATTTTTAAATCCATAAAAGTTGAACCGAATCTATTTTCATATATTATTTTATACATGCAACGACTTACGTTGTAACATAAGGGATGCAATGCATGGCGCAGAA : 2100
 -420 * -400 * -380 * -360 * -340 * -320 * -300 * -280
allele1 : AGTCATAGAGTCGGCAAAGACATCATTTCGTCTGAATCTCTCATGTCCGAGAACCCATACCTCAAGAGCCCAAGACGACACAATACACAACAATCACCGTCAATACCCTTCTCTTCCGCTGCCTATAAATACCAATGGAAATCCCACGAC : 2247
allele2 : AGTCATAGAGTCGGCCAAGAGATCATTTCGTCTGAATCTCTCATGTCCGAGAACCCATACCTCAAGAGCCCAAGACGACACAATACACAACAATCACCGTCAATACCCTTCTCTTCCGCTGCCTATAAATACCAATGGAAATCCCACGAC : 2250
 * -260 * -240 * -220 * -200 * -180 * -160 * -140 *
allele1 : ATTCTCACCAAATCATCATCACTTGAACACACCAATCCTTACACTTCTAGCTACAATTCTAAGTTTCCATTTTCCAACATCCCATCACATTGTTCAAAAATATCATCAGCCTCGAGTTAGGGTTTATTATCCTTCGTGACCCTCCTTTTA : 2397
allele2 : ATTCTCACCAAATCATCATCACTTGAACACACCAATCCTTACACTTCTAGCTACAATTCTAAGTTTCCATTTTCCAACATCCCATCACATTGTTCAAAAATATCATCAGCCTCGAGTTAGGGTTTATTATCCTTCGTGACCCTTCTTTTA : 2400
 -120 * -100 * -80 * -60 * -40 * -20 *
allele1 : GTATTTGGTTCTTTTTGAAAGACGATTTGTTAGGTGTTTCTAGGCCTCTAGCCATCTTGTATCTCACCAAAAAAAAATATATTACCAGCTGTTTACACCAAATTAAATAGTAAAAAGAAAGCATCAATG : 2523
allele2 : GTATTTGGTTCTTTTTGAAAGATGATTTGTTAGGTGTTTCTAGGCCTCTAGCCATCTTGTATCTCACCAAAAAAAAATATATTACCAGCTGTTTACACCAAATTAAATAGTAAAAAGAAAGCATCAATG : 2526

## Slide 4
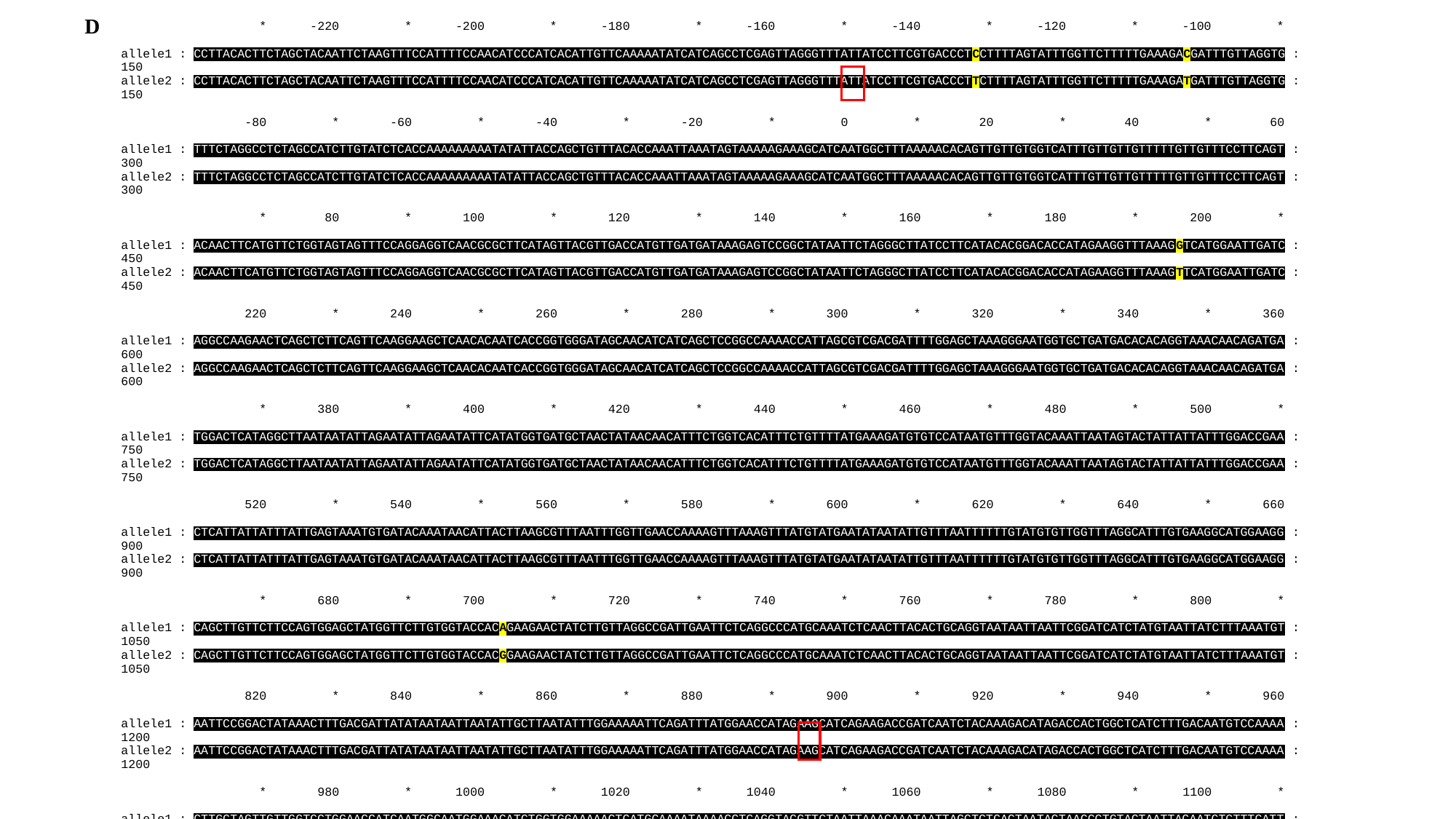

* -220 * -200 * -180 * -160 * -140 * -120 * -100 *
allele1 : CCTTACACTTCTAGCTACAATTCTAAGTTTCCATTTTCCAACATCCCATCACATTGTTCAAAAATATCATCAGCCTCGAGTTAGGGTTTATTATCCTTCGTGACCCTCCTTTTAGTATTTGGTTCTTTTTGAAAGACGATTTGTTAGGTG : 150
allele2 : CCTTACACTTCTAGCTACAATTCTAAGTTTCCATTTTCCAACATCCCATCACATTGTTCAAAAATATCATCAGCCTCGAGTTAGGGTTTATTATCCTTCGTGACCCTTCTTTTAGTATTTGGTTCTTTTTGAAAGATGATTTGTTAGGTG : 150
 -80 * -60 * -40 * -20 * 0 * 20 * 40 * 60
allele1 : TTTCTAGGCCTCTAGCCATCTTGTATCTCACCAAAAAAAAATATATTACCAGCTGTTTACACCAAATTAAATAGTAAAAAGAAAGCATCAATGGCTTTAAAAACACAGTTGTTGTGGTCATTTGTTGTTGTTTTTGTTGTTTCCTTCAGT : 300
allele2 : TTTCTAGGCCTCTAGCCATCTTGTATCTCACCAAAAAAAAATATATTACCAGCTGTTTACACCAAATTAAATAGTAAAAAGAAAGCATCAATGGCTTTAAAAACACAGTTGTTGTGGTCATTTGTTGTTGTTTTTGTTGTTTCCTTCAGT : 300
 * 80 * 100 * 120 * 140 * 160 * 180 * 200 *
allele1 : ACAACTTCATGTTCTGGTAGTAGTTTCCAGGAGGTCAACGCGCTTCATAGTTACGTTGACCATGTTGATGATAAAGAGTCCGGCTATAATTCTAGGGCTTATCCTTCATACACGGACACCATAGAAGGTTTAAAGGTCATGGAATTGATC : 450
allele2 : ACAACTTCATGTTCTGGTAGTAGTTTCCAGGAGGTCAACGCGCTTCATAGTTACGTTGACCATGTTGATGATAAAGAGTCCGGCTATAATTCTAGGGCTTATCCTTCATACACGGACACCATAGAAGGTTTAAAGTTCATGGAATTGATC : 450
 220 * 240 * 260 * 280 * 300 * 320 * 340 * 360
allele1 : AGGCCAAGAACTCAGCTCTTCAGTTCAAGGAAGCTCAACACAATCACCGGTGGGATAGCAACATCATCAGCTCCGGCCAAAACCATTAGCGTCGACGATTTTGGAGCTAAAGGGAATGGTGCTGATGACACACAGGTAAACAACAGATGA : 600
allele2 : AGGCCAAGAACTCAGCTCTTCAGTTCAAGGAAGCTCAACACAATCACCGGTGGGATAGCAACATCATCAGCTCCGGCCAAAACCATTAGCGTCGACGATTTTGGAGCTAAAGGGAATGGTGCTGATGACACACAGGTAAACAACAGATGA : 600
 * 380 * 400 * 420 * 440 * 460 * 480 * 500 *
allele1 : TGGACTCATAGGCTTAATAATATTAGAATATTAGAATATTCATATGGTGATGCTAACTATAACAACATTTCTGGTCACATTTCTGTTTTATGAAAGATGTGTCCATAATGTTTGGTACAAATTAATAGTACTATTATTATTTGGACCGAA : 750
allele2 : TGGACTCATAGGCTTAATAATATTAGAATATTAGAATATTCATATGGTGATGCTAACTATAACAACATTTCTGGTCACATTTCTGTTTTATGAAAGATGTGTCCATAATGTTTGGTACAAATTAATAGTACTATTATTATTTGGACCGAA : 750
 520 * 540 * 560 * 580 * 600 * 620 * 640 * 660
allele1 : CTCATTATTATTTATTGAGTAAATGTGATACAAATAACATTACTTAAGCGTTTAATTTGGTTGAACCAAAAGTTTAAAGTTTATGTATGAATATAATATTGTTTAATTTTTTGTATGTGTTGGTTTAGGCATTTGTGAAGGCATGGAAGG : 900
allele2 : CTCATTATTATTTATTGAGTAAATGTGATACAAATAACATTACTTAAGCGTTTAATTTGGTTGAACCAAAAGTTTAAAGTTTATGTATGAATATAATATTGTTTAATTTTTTGTATGTGTTGGTTTAGGCATTTGTGAAGGCATGGAAGG : 900
 * 680 * 700 * 720 * 740 * 760 * 780 * 800 *
allele1 : CAGCTTGTTCTTCCAGTGGAGCTATGGTTCTTGTGGTACCACAGAAGAACTATCTTGTTAGGCCGATTGAATTCTCAGGCCCATGCAAATCTCAACTTACACTGCAGGTAATAATTAATTCGGATCATCTATGTAATTATCTTTAAATGT : 1050
allele2 : CAGCTTGTTCTTCCAGTGGAGCTATGGTTCTTGTGGTACCACGGAAGAACTATCTTGTTAGGCCGATTGAATTCTCAGGCCCATGCAAATCTCAACTTACACTGCAGGTAATAATTAATTCGGATCATCTATGTAATTATCTTTAAATGT : 1050
 820 * 840 * 860 * 880 * 900 * 920 * 940 * 960
allele1 : AATTCCGGACTATAAACTTTGACGATTATATAATAATTAATATTGCTTAATATTTGGAAAAATTCAGATTTATGGAACCATAGAAGCATCAGAAGACCGATCAATCTACAAAGACATAGACCACTGGCTCATCTTTGACAATGTCCAAAA : 1200
allele2 : AATTCCGGACTATAAACTTTGACGATTATATAATAATTAATATTGCTTAATATTTGGAAAAATTCAGATTTATGGAACCATAGAAGCATCAGAAGACCGATCAATCTACAAAGACATAGACCACTGGCTCATCTTTGACAATGTCCAAAA : 1200
 * 980 * 1000 * 1020 * 1040 * 1060 * 1080 * 1100 *
allele1 : CTTGCTAGTTGTTGGTCCTGGAACCATCAATGGCAATGGAAACATCTGGTGGAAAAACTCATGCAAAATAAAACCTCAGGTACGTTCTAATTAAACAAATAATTAGCTCTCACTAATACTAACCCTGTACTAATTACAATCTCTTTCATT : 1350
allele2 : CTTGCTAGTTGTTGGTCCTGGAACCATCAATGGCAATGGAAACATCTGGTGGAAAAACTCATGCAAAATAAAACCTCAGGTACGTTCTAATTAAACAAATAATTAGCTCTCACTAATACTAACCCTGTACTAATTACAATCTCTTTCATT : 1350
 1120 * 1140 * 1160 * 1180 * 1200 * 1220 * 1240 * 1260
allele1 : GATTTACAGCCCCCTTGCGGTACATACGCCCCCACGGTACGTGTACATACGTACTCTAATCTGAATATGTATGTGCATTTTAATTAATTAAGCACCATTATTTCTGGAACTAATTAAAATTTGTACATGTAATGTGCAGGCTGTGACCTT : 1500
allele2 : GATTTACAGCCCCCTTGCGGTACATACGCCCCCACAGTACGTGTACATACGTACTCTAATCTGAATATGTATGTGCATTTTAATTAATTAAGCACCATTATTTCTGGAACTAATTAAAATTTGTACATGTAATGTGCAGGCTGTGACCTT : 1500
 * 1280 * 1300 * 1320 * 1340 * 1360 * 1380 * 1400 *
allele1 : CAACAGGTGCAATAACTTGGTGGTGAAGAATCTGAATATCCAAGACGCACAACAAATCCATGTCATATTCCAAAACTGCATCAACGTTCAAGCTTCCTGTCTCACGGTAACTGCACCAGAGGACAGCCCTAATACGGACGGAATTCATGT : 1650
allele2 : CAACAGGTGCAATAACTTGGTGGTGAAGAATCTGAATATCCAAGACGCACAACAAATCCATGTCATATTCCAAAACTGCATCAACGTTCAAGCTTCCCGTCTCACGGTAACTGCACCAGAGGACAGCCCTAATACGGACGGAATTCATGT : 1650
 1420 * 1440 * 1460 * 1480 * 1500 * 1520 * 1540 * 1560
allele1 : GACAAATACCCAGAACATCACTATCTCGAGCTCGGTTATAGGAACAGGTGTGACAAATAGTTCATGATGCTTTCAAAAGTCATCATTTTGGTCTCTCGCGTTTGATAAATACATATGATCGATCAAACACATTAAGATAAAAAAACTAAA : 1800
allele2 : GACAAATACCCAGAACATCACTATCTCGAGCTCGGTTATAGGAACAGGTGTGACAAATAGTTCATGACGCTTTCAAAAGTCATCATTTTGGTCTCTCGCGTTTGATAAATACATATGATCGATCAAACACATTAAGATAAAAAAACTAAA : 1800
 * 1580 * 1600 * 1620 * 1640 * 1660 * 1680 * 1700 *
allele1 : CCATATTATGCATAAAATTAACAATCTTATGGTTTCTAATTTGTATTGTGAAGGTGATGACTGTATTTCTATTGTGAGTGGGTCCCAAAGAGTTCAAGCCACAGACATTACTTGTGGACCAGGCCATGGAATCAGGTAATTTCCAACCCT : 1950
allele2 : CCATATTATGCATAAAATTAACAATCTTATGGTTTCTAATTTGTATTGTGAAGGTGATGACTGTATTTCTATTGTGAGTGGGTCCCAAAGAGTTCAAGCCACAGACATTACTTGTGGACCAGGCCATGGAATCAGGTAATTTCCAACCCT : 1950
 1720 * 1740 * 1760 * 1780 * 1800 * 1820 * 1840 * 1860
allele1 : AGTCTCCAAATTGTTCGACAAATCATCCTAAATTCCTCTTGTCAACATATTATTAATGTTTTTTATGTCAAGAAAAGTGCGGGATGACTTTTTATAGTGTCAATAACAGCTGATTAGTAAATATACCAATATATATTAACGGCTGTTTTA : 2100
allele2 : AGTCTCCAAATTGTTCGACAAATCATCCTAAATTCCTCTTGTCAACATATTATTAATGTTTTTTATGTCGAGAAAAGTGCGGGATGACTTTTTATAGTGTCAATAACAGCTGATTAGTAAATATACCAATATATATTAACGGCTGTTTTA : 2100
 * 1880 * 1900 * 1920 * 1940 * 1960 * 1980 * 2000 *
allele1 : ATTTTGTGATCAGTATTGGTAGCTTGGGAGAAGACGGCTCAGAAGATCATGTTTCAGGAGTATTTGTGAATGGAGCTAAGCTTTCAGGAACCTCCAATGGACTCCGGATCAAGACGTGGAAGGGAGGCTCAGGCAGTGCAACCAACATTG : 2250
allele2 : ATTTTGTGATCAGTATCGGTAGCTTGGGAGAAGACGGCTCAGAAGATCATGTTTCAGGAGTATTTGTGAATGGAGCTAAGCTTTCAGGAACCTCCAATGGACTCCGGATCAAGACGTGGAAGGGAGGCTCAGGCAGTGTAACCAACATTG : 2250
 2020 * 2040 * 2060 * 2080 * 2100 * 2120 * 2140 * 2160
allele1 : TTTTCCAGAATGTGCAAATGAACGATGTCACCAACCCCATCATCATCGACCAGAACTACTGTGACCACAAAACCAAAGATTGCAAACAACAGGTAATTAATTAATTTAACTAGTTCTTACGCGTTTCGTTAATTAGTCCCCAGACAAACC : 2400
allele2 : TTTTCCAGAATGTGCAAATGAACGATGTCACCAACCCCATCATCATCGACCAGAACTACTGTGACCACAAAACCAAAGATTGCAAACAACAGGTAATTAATTAATTTAACTAGTTCTTACGCATTTCGTTAATTAGTCCCCAGACAAACC : 2400
 * 2180 * 2200 * 2220 * 2240 * 2260 * 2280 * 2300 *
allele1 : TTAATCACTTCTTGATCGTTTATTTCGTTATGGCAGAAATCGGCGGTCCAAGTGAAAAATGTGTTGTACCAAAACATAAGAGGAACGAGTGCTTCCGGCGACGCGATAACGTTGAACTGCAGCCAAAGTGTTCCTTGTCAGGGGATCGTG : 2550
allele2 : TTAATCACTTCTTGATCGTCTATTTCGTTATGGCAGAAATCGGCGGTTCAAGTGAAAAATGTGTTGTACCAAAACATAAGAGGAACGAGTGCTTCCGGCGACGCGATAACGTTGAACTGCAGCCAAAGTGTTCCTTGTCAGGGGATCGTG : 2550
 2320 * 2340 * 2360 * 2380 * 2400 * 2420 * 2440 * 2460
allele1 : CTGCAAAGTGTTCAACTGCAGAATGGAAGAGCTGAATGCAACAATGTTCAGCCTGCTTACAAAGGAGTTGTCTCCCCTAGATGTTAAAACCTAGGGTTCATAATTATGGGCATTGTGAAATAGATTATGCAATTCTTGTACCAATTAGCA : 2700
allele2 : CTGCAAAGTGTTCAACTGCAGAATGGAAGAGCTGAATGCAACAATGTTCAGCCTGCTTACAAAGGAGTTGTCTCCCCTAGATGTTAAAACCTAGGGTTCATAATTATGGGCATTGTGAAATAGATTATGCAATTCTTGTACCAATTAGCA : 2700
 * 2480 * 2500 * 2520 * 2540
allele1 : CATAAATAATTGTTTGTTTGTAATATTTATGTTTAATTTGGCATTGTACATAAACATATTCATAAATAAAAAGATGCAACTTT : 2783
allele2 : CATAAATAATTGTTTGTTTGTAATATTTATGTTTAATTTGGCATTGTACATAAACATATTCATAAAGAAAAAGATGCAACTGT : 2783
D

## Slide 5
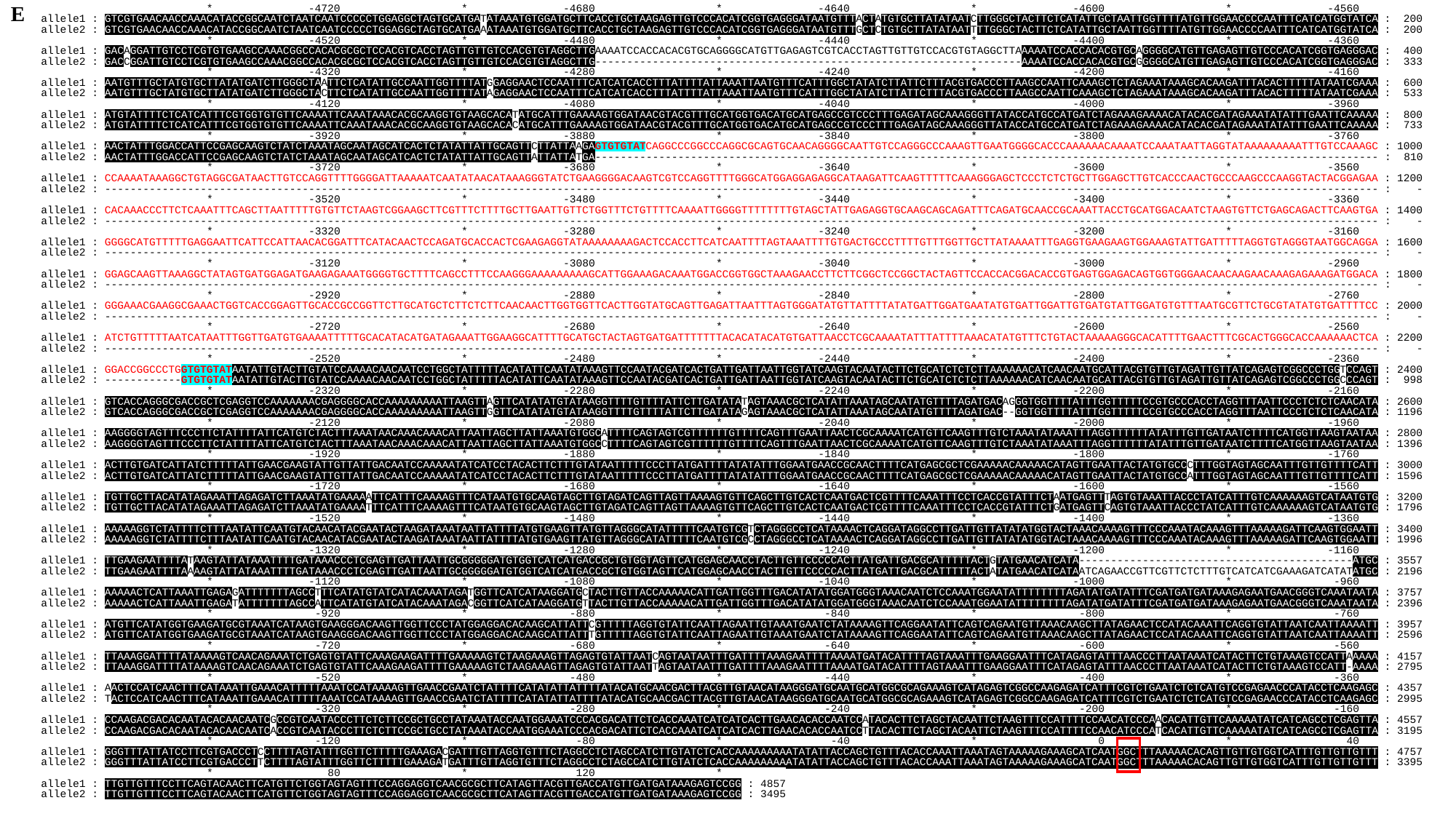

* -4720 * -4680 * -4640 * -4600 * -4560
allele1 : GTCGTGAACAACCAAACATACCGGCAATCTAATCAATCCCCCTGGAGGCTAGTGCATGATATAAATGTGGATGCTTCACCTGCTAAGAGTTGTCCCACATCGGTGAGGGATAATGTTTACTATGTGCTTATATAATCTTGGGCTACTTCTCATATTGCTAATTGGTTTTATGTTGGAACCCCAATTTCATCATGGTATCA : 200
allele2 : GTCGTGAACAACCAAACATACCGGCAATCTAATCAATCCCCCTGGAGGCTAGTGCATGAAATAAATGTGGATGCTTCACCTGCTAAGAGTTGTCCCACATCGGTGAGGGATAATGTTTGCTCTGTGCTTATATAATTTTGGGCTACTTCTCATATTGCTAATTGGTTTTATGTTGGAACCCCAATTTCATCATGGTATCA : 200
 * -4520 * -4480 * -4440 * -4400 * -4360
allele1 : GACAGGATTGTCCTCGTGTGAAGCCAAACGGCCACACGCGCTCCACGTCACCTAGTTGTTGTCCACGTGTAGGCTTGAAAATCCACCACACGTGCAGGGGCATGTTGAGAGTCGTCACCTAGTTGTTGTCCACGTGTAGGCTTAAAAATCCACCACACGTGCAGGGGCATGTTGAGAGTTGTCCCACATCGGTGAGGGAC : 400
allele2 : GACCGGATTGTCCTCGTGTGAAGCCAAACGGCCACACGCGCTCCACGTCACCTAGTTGTTGTCCACGTGTAGGCTTG-------------------------------------------------------------------AAAATCCACCACACGTGCGGGGGCATGTTGAGAGTTGTCCCACATCGGTGAGGGAC : 333
 * -4320 * -4280 * -4240 * -4200 * -4160
allele1 : AATGTTTGCTATGTGCTTATATGATCTTGGGCTAATTCTCATATTGCCAATTGGTTTTATGGAGGAACTCCAATTTCATCATCACCTTTATTTTATTAAATTAATGTTTCATTTGGCTATATCTTATTCTTTACGTGACCCTTAAGCCAATTCAAAGCTCTAGAAATAAAGCACAAGATTTACACTTTTTATAATCGAAA : 600
allele2 : AATGTTTGCTATGTGCTTATATGATCTTGGGCTACTTCTCATATTGCCAATTGGTTTTATAGAGGAACTCCAATTTCATCATCACCTTTATTTTATTAAATTAATGTTTCATTTGGCTATATCTTATTCTTTACGTGACCCTTAAGCCAATTCAAAGCTCTAGAAATAAAGCACAAGATTTACACTTTTTATAATCGAAA : 533
 * -4120 * -4080 * -4040 * -4000 * -3960
allele1 : ATGTATTTTCTCATCATTTCGTGGTGTGTTCAAAATTCAAATAAACACGCAAGGTGTAAGCACATATGCATTTGAAAAGTGGATAACGTACGTTTGCATGGTGACATGCATGAGCCGTCCCTTTGAGATAGCAAAGGGTTATACCATGCCATGATCTAGAAAGAAAACATACACGATAGAAATATATTTGAATTCAAAAA : 800
allele2 : ATGTATTTTCTCATCATTTCGTGGTGTGTTCAAAATTCAAATAAACACGCAAGGTGTAAGCACACATGCATTTGAAAAGTGGATAACGTACGTTTGCATGGTGACATGCATGAGCCGTCCCTTTGAGATAGCAAAGGGTTATACCATGCCATGATCTAGAAAGAAAACATACACGATAGAAATATATTTGAATTCAAAAA : 733
 * -3920 * -3880 * -3840 * -3800 * -3760
allele1 : AACTATTTGGACCATTCCGAGCAAGTCTATCTAAATAGCAATAGCATCACTCTATATTATTGCAGTTCTTATTAAGAGTGTGTATCAGGCCCGGCCCAGGCGCAGTGCAACAGGGGCAATTGTCCAGGGCCCAAAGTTGAATGGGGCACCCAAAAAACAAAATCCAAATAATTAGGTATAAAAAAAAATTTGTCCAAAGC : 1000
allele2 : AACTATTTGGACCATTCCGAGCAAGTCTATCTAAATAGCAATAGCATCACTCTATATTATTGCAGTTATTATTATGA--------------------------------------------------------------------------------------------------------------------------- : 810
 * -3720 * -3680 * -3640 * -3600 * -3560
allele1 : CCAAAATAAAGGCTGTAGGCGATAACTTGTCCAGGTTTTGGGGATTAAAAATCAATATAACATAAAGGGTATCTGAAGGGGACAAGTCGTCCAGGTTTTGGGCATGGAGGAGAGGCATAAGATTCAAGTTTTTCAAAGGGAGCTCCCTCTCTGCTTGGAGCTTGTCACCCAACTGCCCAAGCCCAAGGTACTACGGAGAA : 1200
allele2 : -------------------------------------------------------------------------------------------------------------------------------------------------------------------------------------------------------- : -
 * -3520 * -3480 * -3440 * -3400 * -3360
allele1 : CACAAACCCTTCTCAAATTTCAGCTTAATTTTTGTGTTCTAAGTCGGAAGCTTCGTTTCTTTTGCTTGAATTGTTCTGGTTTCTGTTTTCAAAATTGGGGTTTTTTTTGTAGCTATTGAGAGGTGCAAGCAGCAGATTTCAGATGCAACCGCAAATTACCTGCATGGACAATCTAAGTGTTCTGAGCAGACTTCAAGTGA : 1400
allele2 : -------------------------------------------------------------------------------------------------------------------------------------------------------------------------------------------------------- : -
 * -3320 * -3280 * -3240 * -3200 * -3160
allele1 : GGGGCATGTTTTTGAGGAATTCATTCCATTAACACGGATTTCATACAACTCCAGATGCACCACTCGAAGAGGTATAAAAAAAAGACTCCACCTTCATCAATTTTAGTAAATTTTGTGACTGCCCTTTTGTTTGGTTGCTTATAAAATTTGAGGTGAAGAAGTGGAAAGTATTGATTTTTAGGTGTAGGGTAATGGCAGGA : 1600
allele2 : -------------------------------------------------------------------------------------------------------------------------------------------------------------------------------------------------------- : -
 * -3120 * -3080 * -3040 * -3000 * -2960
allele1 : GGAGCAAGTTAAAGGCTATAGTGATGGAGATGAAGAGAAATGGGGTGCTTTTCAGCCTTTCCAAGGGAAAAAAAAAGCATTGGAAAGACAAATGGACCGGTGGCTAAAGAACCTTCTTCGGCTCCGGCTACTAGTTCCACCACGGACACCGTGAGTGGAGACAGTGGTGGGAACAACAAGAACAAAGAGAAAGATGGACA : 1800
allele2 : -------------------------------------------------------------------------------------------------------------------------------------------------------------------------------------------------------- : -
 * -2920 * -2880 * -2840 * -2800 * -2760
allele1 : GGGAAACGAAGGCGAAACTGGTCACCGGAGTTGCACCGCCGGTTCTTGCATGCTCTTCTCTTCAACAACTTGGTGGTTCACTTGGTATGCAGTTGAGATTAATTTAGTGGGATATGTTATTTTATATGATTGGATGAATATGTGATTGGATTGTGATGTATTGGATGTGTTTAATGCGTTCTGCGTATATGTGATTTTCC : 2000
allele2 : -------------------------------------------------------------------------------------------------------------------------------------------------------------------------------------------------------- : -
 * -2720 * -2680 * -2640 * -2600 * -2560
allele1 : ATCTGTTTTTAATCATAATTTGGTTGATGTGAAAATTTTTGCACATACATGATAGAAATTGGAAGGCATTTTGCATGCTACTAGTGATGATTTTTTTACACATACATGTGATTAACCTCGCAAAATATTTATTTTAAACATATGTTTCTGTACTAAAAAGGGCACATTTTGAACTTTCGCACTGGGCACCAAAAAACTCA : 2200
allele2 : -------------------------------------------------------------------------------------------------------------------------------------------------------------------------------------------------------- : -
 * -2520 * -2480 * -2440 * -2400 * -2360
allele1 : GGACCGGCCCTGGTGTGTATAATATTGTACTTGTATCCAAAACAACAATCCTGGCTATTTTTACATATTCAATATAAAGTTCCAATACGATCACTGATTGATTAATTGGTATCAAGTACAATACTTCTGCATCTCTCTTAAAAAACATCAACAATGCATTACGTGTTGTAGATTGTTATCAGAGTCGGCCCTGGTCCAGT : 2400
allele2 : ------------GTGTGTATAATATTGTACTTGTATCCAAAACAACAATCCTGGCTATTTTTACATATTCAATATAAAGTTCCAATACGATCACTGATTGATTAATTGGTATCAAGTACAATACTTCTGCATCTCTCTTAAAAAACATCAACAATGCATTACGTGTTGTAGATTGTTATCAGAGTCGGCCCTGGCCCAGT : 998
 * -2320 * -2280 * -2240 * -2200 * -2160
allele1 : GTCACCAGGGCGACCGCTCGAGGTCCAAAAAAACGAGGGGCACCAAAAAAAAATTAAGTTAGTTCATATATGTATAAGGTTTTGTTTTATTCTTGATATATAGTAAACGCTCATATTAAATAGCAATATGTTTTAGATGACAGGGTGGTTTTATTTGGTTTTTCCGTGCCCACCTAGGTTTAATTCCCTCTCTCAACATA : 2600
allele2 : GTCACCAGGGCGACCGCTCGAGGTCCAAAAAAACGAGGGGCACCAAAAAAAAATTAAGTTGGTTCATATATGTATAAGGTTTTGTTTTATTCTTGATATAGAGTAAACGCTCATATTAAATAGCAATATGTTTTAGATGAC--GGTGGTTTTATTTGGTTTTTCCGTGCCCACCTAGGTTTAATTCCCTCTCTCAACATA : 1196
 * -2120 * -2080 * -2040 * -2000 * -1960
allele1 : AAGGGGTAGTTTCCCTTCTATTTTATTCATGTCTACTTTAAATAACAAACAAACATTAATTAGCTTATTAAATGTGGCATTTTCAGTAGTCGTTTTTTGTTTTCAGTTTGAATTAACTCGCAAAATCATGTTCAAGTTTGTCTAAATATAAATTTAGGTTTTTTATATTTGTTGATAATCTTTTCATGGTTAAGTAATAA : 2800
allele2 : AAGGGGTAGTTTCCCTTCTATTTTATTCATGTCTACTTTAAATAACAAACAAACATTAATTAGCTTATTAAATGTGGCCTTTTCAGTAGTCGTTTTTTGTTTTCAGTTTGAATTAACTCGCAAAATCATGTTCAAGTTTGTCTAAATATAAATTTAGGTTTTTTATATTTGTTGATAATCTTTTCATGGTTAAGTAATAA : 1396
 * -1920 * -1880 * -1840 * -1800 * -1760
allele1 : ACTTGTGATCATTATCTTTTTATTGAACGAAGTATTGTTATTGACAATCCAAAAATATCATCCTACACTTCTTTGTATAATTTTTCCCTTATGATTTTATATATTTGGAATGAACCGCAACTTTTCATGAGCGCTCGAAAAACAAAAACATAGTTGAATTACTATGTGCCCTTTGGTAGTAGCAATTTGTTGTTTTCATT : 3000
allele2 : ACTTGTGATCATTATCTTTTTATTGAACGAAGTATTGTTATTGACAATCCAAAAATATCATCCTACACTTCTTTGTATAATTTTTCCCTTATGATTTTATATATTTGGAATGAACCGCAACTTTTCATGAGCGCTCGAAAAACAAAAACATAGTTGAATTACTATGTGCCATTTGGTAGTAGCAATTTGTTGTTTTCATT : 1596
 * -1720 * -1680 * -1640 * -1600 * -1560
allele1 : TGTTGCTTACATATAGAAATTAGAGATCTTAAATATGAAAAATTCATTTCAAAAGTTTCATAATGTGCAAGTAGCTTGTAGATCAGTTAGTTAAAAGTGTTCAGCTTGTCACTCAATGACTCGTTTTCAAATTTCCTCACCGTATTTCTAATGAGTTTAGTGTAAATTACCCTATCATTTGTCAAAAAAGTCATAATGTG : 3200
allele2 : TGTTGCTTACATATAGAAATTAGAGATCTTAAATATGAAAATTTCATTTCAAAAGTTTCATAATGTGCAAGTAGCTTGTAGATCAGTTAGTTAAAAGTGTTCAGCTTGTCACTCAATGACTCGTTTTCAAATTTCCTCACCGTATTTCTGATGAGTTCAGTGTAAATTACCCTATCATTTGTCAAAAAAGTCATAATGTG : 1796
 * -1520 * -1480 * -1440 * -1400 * -1360
allele1 : AAAAAGGTCTATTTTCTTTAATATTCAATGTACAACATACGAATACTAAGATAAATAATTATTTTATGTGAAGTTATGTTAGGGCATATTTTTCAATGTCGTCTAGGGCCTCATAAAACTCAGGATAGGCCTTGATTGTTATATATGGTACTAAACAAAAGTTTCCCAAATACAAAGTTTAAAAAGATTCAAGTGGAATT : 3400
allele2 : AAAAAGGTCTATTTTCTTTAATATTCAATGTACAACATACGAATACTAAGATAAATAATTATTTTATGTGAAGTTATGTTAGGGCATATTTTTCAATGTCGCCTAGGGCCTCATAAAACTCAGGATAGGCCTTGATTGTTATATATGGTACTAAACAAAAGTTTCCCAAATACAAAGTTTAAAAAGATTCAAGTGGAATT : 1996
 * -1320 * -1280 * -1240 * -1200 * -1160
allele1 : TTGAAGAATTTTATAAGTATTATAAATTTTGATAAACCCTCGAGTTGATTAATTGCGGGGGATGTGGTCATCATGACCGCTGTGGTAGTTCATGGAGCAACCTACTTGTTCCCCCACTTATGATTGACGCATTTTTACTGTATGAACATCATA-------------------------------------------ATGC : 3557
allele2 : TTGAAGAATTTTAAAAGTATTATAAATTTTGATAAACCCTCGAGTTGATTAATTGCGGGGGATGTGGTCATCATGACCGCTGTGGTAGTTCATGGAGCAACCTACTTGTTCCCCCACTTATGATTGACGCATTTTTACTATATGAACATCATAATCAGAACCGTTCGTTCTCTTTGTCATCATCGAAAGATCATATATGC : 2196
 * -1120 * -1080 * -1040 * -1000 * -960
allele1 : AAAAACTCATTAAATTGAGAGATTTTTTTAGCCTTTCATATGTATCATACAAATAGATGGTTCATCATAAGGATGCTACTTGTTACCAAAAACATTGATTGGTTTGACATATATGGATGGGTAAACAATCTCCAAATGGAATATTTTTTTTAGATATGATATTTCGATGATGATAAAGAGAATGAACGGGTCAAATAATA : 3757
allele2 : AAAAACTCATTAAATTGAGATATTTTTTTAGCCATTCATATGTATCATACAAATAGACGGTTCATCATAAGGATGTTACTTGTTACCAAAAACATTGATTGGTTTGACATATATGGATGGGTAAACAATCTCCAAATGGAATATTTTTTTTAGATATGATATTTCGATGATGATAAAGAGAATGAACGGGTCAAATAATA : 2396
 * -920 * -880 * -840 * -800 * -760
allele1 : ATGTTCATATGGTGAAGATGCGTAAATCATAAGTGAAGGGACAAGTTGGTTCCCTATGGAGGACACAAGCATTATTCGTTTTTAGGTGTATTCAATTAGAATTGTAAATGAATCTATAAAAGTTCAGGAATATTCAGTCAGAATGTTAAACAAGCTTATAGAACTCCATACAAATTCAGGTGTATTAATCAATTAAAATT : 3957
allele2 : ATGTTCATATGGTGAAGATGCGTAAATCATAAGTGAAGGGACAAGTTGGTTCCCTATGGAGGACACAAGCATTATTTGTTTTTAGGTGTATTCAATTAGAATTGTAAATGAATCTATAAAAGTTCAGGAATATTCAGTCAGAATGTTAAACAAGCTTATAGAACTCCATACAAATTCAGGTGTATTAATCAATTAAAATT : 2596
 * -720 * -680 * -640 * -600 * -560
allele1 : TTAAAGGATTTTATAAAAGTCAACAGAAATCTGAGTGTATTCAAAGAAGATTTTGAAAAAGTCTAAGAAAGTTAGAGTGTATTAATCAGTAATAATTTGATTTTAAAGAATTTTAAAATGATACATTTTAGTAAATTTGAAGGAATTTCATAGAGTATTTAACCCTTAATAAATCATACTTCTGTAAAGTCCATTAAAAA : 4157
allele2 : TTAAAGGATTTTATAAAAGTCAACAGAAATCTGAGTGTATTCAAAGAAGATTTTGAAAAAGTCTAAGAAAGTTAGAGTGTATTAATTAGTAATAATTTGATTTTAAAGAATTTTAAAATGATACATTTTAGTAAATTTGAAGGAATTTCATAGAGTATTTAACCCTTAATAAATCATACTTCTGTAAAGTCCATT-AAAA : 2795
 * -520 * -480 * -440 * -400 * -360
allele1 : AACTCCATCAACTTTCATAAATTGAAACATTTTTAAATCCATAAAAGTTGAACCGAATCTATTTTCATATATTATTTTATACATGCAACGACTTACGTTGTAACATAAGGGATGCAATGCATGGCGCAGAAAGTCATAGAGTCGGCCAAGAGATCATTTCGTCTGAATCTCTCATGTCCGAGAACCCATACCTCAAGAGC : 4357
allele2 : TACTCCATCAACTTTCATAAATTGAAACATTTTTAAATCCATAAAAGTTGAACCGAATCTATTTTCATATATTATTTTATACATGCAACGACTTACGTTGTAACATAAGGGATGCAATGCATGGCGCAGAAAGTCATAGAGTCGGCCAAGAGATCATTTCGTCTGAATCTCTCATGTCCGAGAACCCATACCTCAAGAGC : 2995
 * -320 * -280 * -240 * -200 * -160
allele1 : CCAAGACGACACAATACACAACAATCGCCGTCAATACCCTTCTCTTCCGCTGCCTATAAATACCAATGGAAATCCCACGACATTCTCACCAAATCATCATCACTTGAACACACCAATCCATACACTTCTAGCTACAATTCTAAGTTTCCATTTTCCAACATCCCAACACATTGTTCAAAAATATCATCAGCCTCGAGTTA : 4557
allele2 : CCAAGACGACACAATACACAACAATCACCGTCAATACCCTTCTCTTCCGCTGCCTATAAATACCAATGGAAATCCCACGACATTCTCACCAAATCATCATCACTTGAACACACCAATCCTTACACTTCTAGCTACAATTCTAAGTTTCCATTTTCCAACATCCCATCACATTGTTCAAAAATATCATCAGCCTCGAGTTA : 3195
 * -120 * -80 * -40 * 0 * 40
allele1 : GGGTTTATTATCCTTCGTGACCCTCCTTTTAGTATTTGGTTCTTTTTGAAAGACGATTTGTTAGGTGTTTCTAGGCCTCTAGCCATCTTGTATCTCACCAAAAAAAAATATATTACCAGCTGTTTACACCAAATTAAATAGTAAAAAGAAAGCATCAATGGCTTTAAAAACACAGTTGTTGTGGTCATTTGTTGTTGTTT : 4757
allele2 : GGGTTTATTATCCTTCGTGACCCTTCTTTTAGTATTTGGTTCTTTTTGAAAGATGATTTGTTAGGTGTTTCTAGGCCTCTAGCCATCTTGTATCTCACCAAAAAAAAATATATTACCAGCTGTTTACACCAAATTAAATAGTAAAAAGAAAGCATCAATGGCTTTAAAAACACAGTTGTTGTGGTCATTTGTTGTTGTTT : 3395
 * 80 * 120 *
allele1 : TTGTTGTTTCCTTCAGTACAACTTCATGTTCTGGTAGTAGTTTCCAGGAGGTCAACGCGCTTCATAGTTACGTTGACCATGTTGATGATAAAGAGTCCGG : 4857
allele2 : TTGTTGTTTCCTTCAGTACAACTTCATGTTCTGGTAGTAGTTTCCAGGAGGTCAACGCGCTTCATAGTTACGTTGACCATGTTGATGATAAAGAGTCCGG : 3495
E

## Slide 6
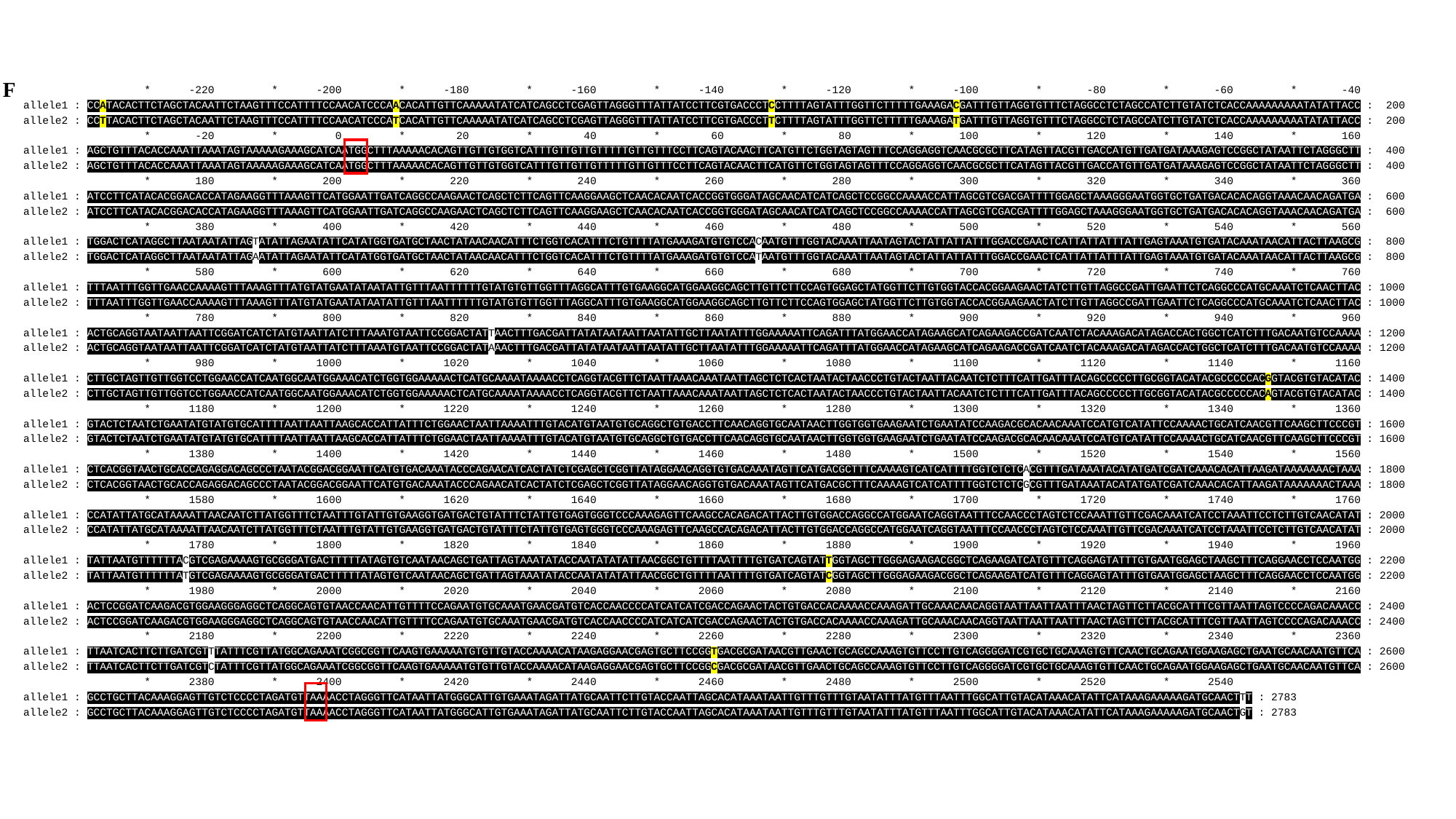

* -220 * -200 * -180 * -160 * -140 * -120 * -100 * -80 * -60 * -40
allele1 : CCATACACTTCTAGCTACAATTCTAAGTTTCCATTTTCCAACATCCCAACACATTGTTCAAAAATATCATCAGCCTCGAGTTAGGGTTTATTATCCTTCGTGACCCTCCTTTTAGTATTTGGTTCTTTTTGAAAGACGATTTGTTAGGTGTTTCTAGGCCTCTAGCCATCTTGTATCTCACCAAAAAAAAATATATTACC : 200
allele2 : CCTTACACTTCTAGCTACAATTCTAAGTTTCCATTTTCCAACATCCCATCACATTGTTCAAAAATATCATCAGCCTCGAGTTAGGGTTTATTATCCTTCGTGACCCTTCTTTTAGTATTTGGTTCTTTTTGAAAGATGATTTGTTAGGTGTTTCTAGGCCTCTAGCCATCTTGTATCTCACCAAAAAAAAATATATTACC : 200
 * -20 * 0 * 20 * 40 * 60 * 80 * 100 * 120 * 140 * 160
allele1 : AGCTGTTTACACCAAATTAAATAGTAAAAAGAAAGCATCAATGGCTTTAAAAACACAGTTGTTGTGGTCATTTGTTGTTGTTTTTGTTGTTTCCTTCAGTACAACTTCATGTTCTGGTAGTAGTTTCCAGGAGGTCAACGCGCTTCATAGTTACGTTGACCATGTTGATGATAAAGAGTCCGGCTATAATTCTAGGGCTT : 400
allele2 : AGCTGTTTACACCAAATTAAATAGTAAAAAGAAAGCATCAATGGCTTTAAAAACACAGTTGTTGTGGTCATTTGTTGTTGTTTTTGTTGTTTCCTTCAGTACAACTTCATGTTCTGGTAGTAGTTTCCAGGAGGTCAACGCGCTTCATAGTTACGTTGACCATGTTGATGATAAAGAGTCCGGCTATAATTCTAGGGCTT : 400
 * 180 * 200 * 220 * 240 * 260 * 280 * 300 * 320 * 340 * 360
allele1 : ATCCTTCATACACGGACACCATAGAAGGTTTAAAGTTCATGGAATTGATCAGGCCAAGAACTCAGCTCTTCAGTTCAAGGAAGCTCAACACAATCACCGGTGGGATAGCAACATCATCAGCTCCGGCCAAAACCATTAGCGTCGACGATTTTGGAGCTAAAGGGAATGGTGCTGATGACACACAGGTAAACAACAGATGA : 600
allele2 : ATCCTTCATACACGGACACCATAGAAGGTTTAAAGTTCATGGAATTGATCAGGCCAAGAACTCAGCTCTTCAGTTCAAGGAAGCTCAACACAATCACCGGTGGGATAGCAACATCATCAGCTCCGGCCAAAACCATTAGCGTCGACGATTTTGGAGCTAAAGGGAATGGTGCTGATGACACACAGGTAAACAACAGATGA : 600
 * 380 * 400 * 420 * 440 * 460 * 480 * 500 * 520 * 540 * 560
allele1 : TGGACTCATAGGCTTAATAATATTAGTATATTAGAATATTCATATGGTGATGCTAACTATAACAACATTTCTGGTCACATTTCTGTTTTATGAAAGATGTGTCCACAATGTTTGGTACAAATTAATAGTACTATTATTATTTGGACCGAACTCATTATTATTTATTGAGTAAATGTGATACAAATAACATTACTTAAGCG : 800
allele2 : TGGACTCATAGGCTTAATAATATTAGAATATTAGAATATTCATATGGTGATGCTAACTATAACAACATTTCTGGTCACATTTCTGTTTTATGAAAGATGTGTCCATAATGTTTGGTACAAATTAATAGTACTATTATTATTTGGACCGAACTCATTATTATTTATTGAGTAAATGTGATACAAATAACATTACTTAAGCG : 800
 * 580 * 600 * 620 * 640 * 660 * 680 * 700 * 720 * 740 * 760
allele1 : TTTAATTTGGTTGAACCAAAAGTTTAAAGTTTATGTATGAATATAATATTGTTTAATTTTTTGTATGTGTTGGTTTAGGCATTTGTGAAGGCATGGAAGGCAGCTTGTTCTTCCAGTGGAGCTATGGTTCTTGTGGTACCACGGAAGAACTATCTTGTTAGGCCGATTGAATTCTCAGGCCCATGCAAATCTCAACTTAC : 1000
allele2 : TTTAATTTGGTTGAACCAAAAGTTTAAAGTTTATGTATGAATATAATATTGTTTAATTTTTTGTATGTGTTGGTTTAGGCATTTGTGAAGGCATGGAAGGCAGCTTGTTCTTCCAGTGGAGCTATGGTTCTTGTGGTACCACGGAAGAACTATCTTGTTAGGCCGATTGAATTCTCAGGCCCATGCAAATCTCAACTTAC : 1000
 * 780 * 800 * 820 * 840 * 860 * 880 * 900 * 920 * 940 * 960
allele1 : ACTGCAGGTAATAATTAATTCGGATCATCTATGTAATTATCTTTAAATGTAATTCCGGACTATTAACTTTGACGATTATATAATAATTAATATTGCTTAATATTTGGAAAAATTCAGATTTATGGAACCATAGAAGCATCAGAAGACCGATCAATCTACAAAGACATAGACCACTGGCTCATCTTTGACAATGTCCAAAA : 1200
allele2 : ACTGCAGGTAATAATTAATTCGGATCATCTATGTAATTATCTTTAAATGTAATTCCGGACTATAAACTTTGACGATTATATAATAATTAATATTGCTTAATATTTGGAAAAATTCAGATTTATGGAACCATAGAAGCATCAGAAGACCGATCAATCTACAAAGACATAGACCACTGGCTCATCTTTGACAATGTCCAAAA : 1200
 * 980 * 1000 * 1020 * 1040 * 1060 * 1080 * 1100 * 1120 * 1140 * 1160
allele1 : CTTGCTAGTTGTTGGTCCTGGAACCATCAATGGCAATGGAAACATCTGGTGGAAAAACTCATGCAAAATAAAACCTCAGGTACGTTCTAATTAAACAAATAATTAGCTCTCACTAATACTAACCCTGTACTAATTACAATCTCTTTCATTGATTTACAGCCCCCTTGCGGTACATACGCCCCCACGGTACGTGTACATAC : 1400
allele2 : CTTGCTAGTTGTTGGTCCTGGAACCATCAATGGCAATGGAAACATCTGGTGGAAAAACTCATGCAAAATAAAACCTCAGGTACGTTCTAATTAAACAAATAATTAGCTCTCACTAATACTAACCCTGTACTAATTACAATCTCTTTCATTGATTTACAGCCCCCTTGCGGTACATACGCCCCCACAGTACGTGTACATAC : 1400
 * 1180 * 1200 * 1220 * 1240 * 1260 * 1280 * 1300 * 1320 * 1340 * 1360
allele1 : GTACTCTAATCTGAATATGTATGTGCATTTTAATTAATTAAGCACCATTATTTCTGGAACTAATTAAAATTTGTACATGTAATGTGCAGGCTGTGACCTTCAACAGGTGCAATAACTTGGTGGTGAAGAATCTGAATATCCAAGACGCACAACAAATCCATGTCATATTCCAAAACTGCATCAACGTTCAAGCTTCCCGT : 1600
allele2 : GTACTCTAATCTGAATATGTATGTGCATTTTAATTAATTAAGCACCATTATTTCTGGAACTAATTAAAATTTGTACATGTAATGTGCAGGCTGTGACCTTCAACAGGTGCAATAACTTGGTGGTGAAGAATCTGAATATCCAAGACGCACAACAAATCCATGTCATATTCCAAAACTGCATCAACGTTCAAGCTTCCCGT : 1600
 * 1380 * 1400 * 1420 * 1440 * 1460 * 1480 * 1500 * 1520 * 1540 * 1560
allele1 : CTCACGGTAACTGCACCAGAGGACAGCCCTAATACGGACGGAATTCATGTGACAAATACCCAGAACATCACTATCTCGAGCTCGGTTATAGGAACAGGTGTGACAAATAGTTCATGACGCTTTCAAAAGTCATCATTTTGGTCTCTCACGTTTGATAAATACATATGATCGATCAAACACATTAAGATAAAAAAACTAAA : 1800
allele2 : CTCACGGTAACTGCACCAGAGGACAGCCCTAATACGGACGGAATTCATGTGACAAATACCCAGAACATCACTATCTCGAGCTCGGTTATAGGAACAGGTGTGACAAATAGTTCATGACGCTTTCAAAAGTCATCATTTTGGTCTCTCGCGTTTGATAAATACATATGATCGATCAAACACATTAAGATAAAAAAACTAAA : 1800
 * 1580 * 1600 * 1620 * 1640 * 1660 * 1680 * 1700 * 1720 * 1740 * 1760
allele1 : CCATATTATGCATAAAATTAACAATCTTATGGTTTCTAATTTGTATTGTGAAGGTGATGACTGTATTTCTATTGTGAGTGGGTCCCAAAGAGTTCAAGCCACAGACATTACTTGTGGACCAGGCCATGGAATCAGGTAATTTCCAACCCTAGTCTCCAAATTGTTCGACAAATCATCCTAAATTCCTCTTGTCAACATAT : 2000
allele2 : CCATATTATGCATAAAATTAACAATCTTATGGTTTCTAATTTGTATTGTGAAGGTGATGACTGTATTTCTATTGTGAGTGGGTCCCAAAGAGTTCAAGCCACAGACATTACTTGTGGACCAGGCCATGGAATCAGGTAATTTCCAACCCTAGTCTCCAAATTGTTCGACAAATCATCCTAAATTCCTCTTGTCAACATAT : 2000
 * 1780 * 1800 * 1820 * 1840 * 1860 * 1880 * 1900 * 1920 * 1940 * 1960
allele1 : TATTAATGTTTTTTACGTCGAGAAAAGTGCGGGATGACTTTTTATAGTGTCAATAACAGCTGATTAGTAAATATACCAATATATATTAACGGCTGTTTTAATTTTGTGATCAGTATTGGTAGCTTGGGAGAAGACGGCTCAGAAGATCATGTTTCAGGAGTATTTGTGAATGGAGCTAAGCTTTCAGGAACCTCCAATGG : 2200
allele2 : TATTAATGTTTTTTATGTCGAGAAAAGTGCGGGATGACTTTTTATAGTGTCAATAACAGCTGATTAGTAAATATACCAATATATATTAACGGCTGTTTTAATTTTGTGATCAGTATCGGTAGCTTGGGAGAAGACGGCTCAGAAGATCATGTTTCAGGAGTATTTGTGAATGGAGCTAAGCTTTCAGGAACCTCCAATGG : 2200
 * 1980 * 2000 * 2020 * 2040 * 2060 * 2080 * 2100 * 2120 * 2140 * 2160
allele1 : ACTCCGGATCAAGACGTGGAAGGGAGGCTCAGGCAGTGTAACCAACATTGTTTTCCAGAATGTGCAAATGAACGATGTCACCAACCCCATCATCATCGACCAGAACTACTGTGACCACAAAACCAAAGATTGCAAACAACAGGTAATTAATTAATTTAACTAGTTCTTACGCATTTCGTTAATTAGTCCCCAGACAAACC : 2400
allele2 : ACTCCGGATCAAGACGTGGAAGGGAGGCTCAGGCAGTGTAACCAACATTGTTTTCCAGAATGTGCAAATGAACGATGTCACCAACCCCATCATCATCGACCAGAACTACTGTGACCACAAAACCAAAGATTGCAAACAACAGGTAATTAATTAATTTAACTAGTTCTTACGCATTTCGTTAATTAGTCCCCAGACAAACC : 2400
 * 2180 * 2200 * 2220 * 2240 * 2260 * 2280 * 2300 * 2320 * 2340 * 2360
allele1 : TTAATCACTTCTTGATCGTTTATTTCGTTATGGCAGAAATCGGCGGTTCAAGTGAAAAATGTGTTGTACCAAAACATAAGAGGAACGAGTGCTTCCGGTGACGCGATAACGTTGAACTGCAGCCAAAGTGTTCCTTGTCAGGGGATCGTGCTGCAAAGTGTTCAACTGCAGAATGGAAGAGCTGAATGCAACAATGTTCA : 2600
allele2 : TTAATCACTTCTTGATCGTCTATTTCGTTATGGCAGAAATCGGCGGTTCAAGTGAAAAATGTGTTGTACCAAAACATAAGAGGAACGAGTGCTTCCGGCGACGCGATAACGTTGAACTGCAGCCAAAGTGTTCCTTGTCAGGGGATCGTGCTGCAAAGTGTTCAACTGCAGAATGGAAGAGCTGAATGCAACAATGTTCA : 2600
 * 2380 * 2400 * 2420 * 2440 * 2460 * 2480 * 2500 * 2520 * 2540
allele1 : GCCTGCTTACAAAGGAGTTGTCTCCCCTAGATGTTAAAACCTAGGGTTCATAATTATGGGCATTGTGAAATAGATTATGCAATTCTTGTACCAATTAGCACATAAATAATTGTTTGTTTGTAATATTTATGTTTAATTTGGCATTGTACATAAACATATTCATAAAGAAAAAGATGCAACTTT : 2783
allele2 : GCCTGCTTACAAAGGAGTTGTCTCCCCTAGATGTTAAAACCTAGGGTTCATAATTATGGGCATTGTGAAATAGATTATGCAATTCTTGTACCAATTAGCACATAAATAATTGTTTGTTTGTAATATTTATGTTTAATTTGGCATTGTACATAAACATATTCATAAAGAAAAAGATGCAACTGT : 2783
F

## Slide 7
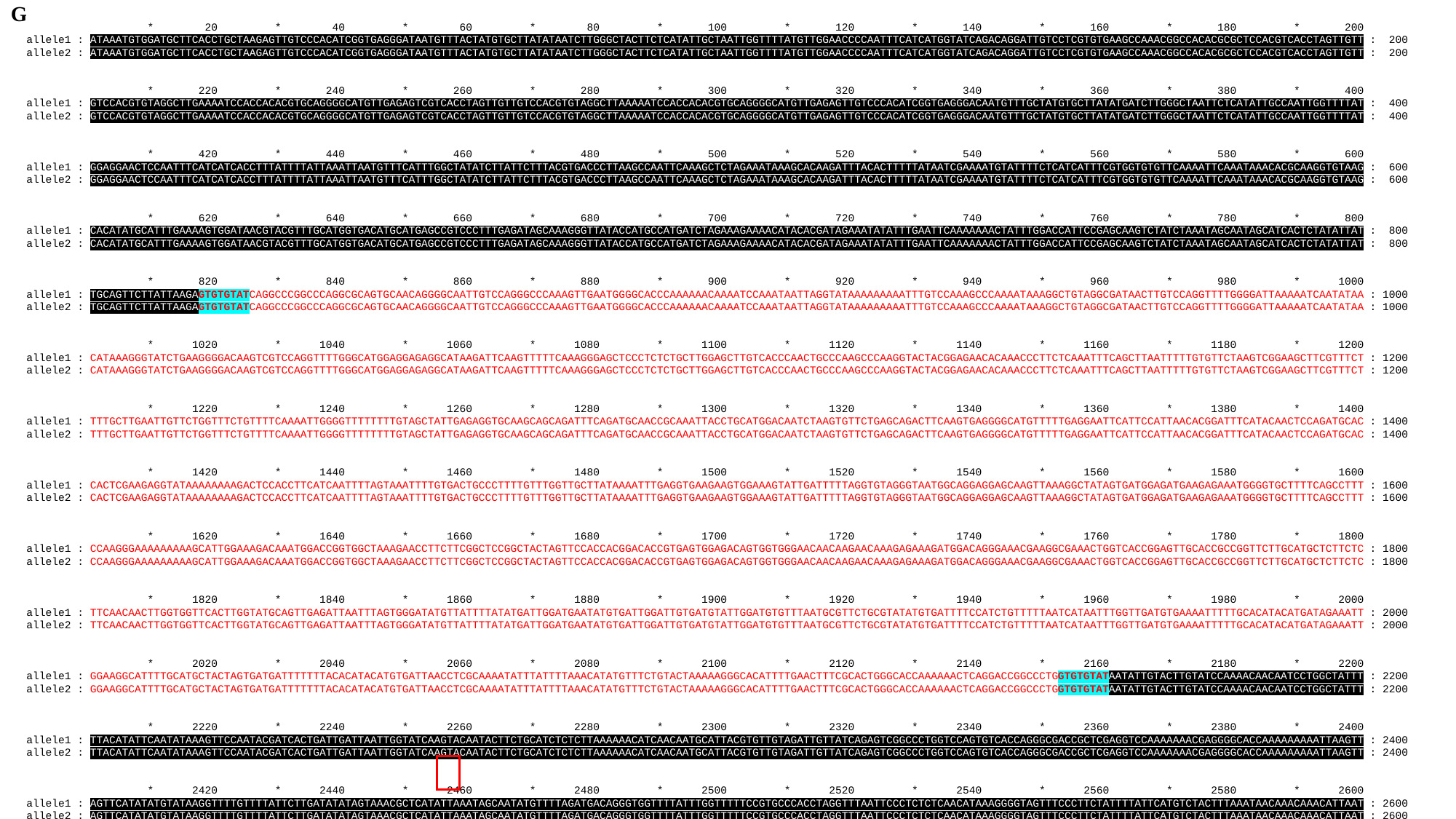

G
 * 20 * 40 * 60 * 80 * 100 * 120 * 140 * 160 * 180 * 200
allele1 : ATAAATGTGGATGCTTCACCTGCTAAGAGTTGTCCCACATCGGTGAGGGATAATGTTTACTATGTGCTTATATAATCTTGGGCTACTTCTCATATTGCTAATTGGTTTTATGTTGGAACCCCAATTTCATCATGGTATCAGACAGGATTGTCCTCGTGTGAAGCCAAACGGCCACACGCGCTCCACGTCACCTAGTTGTT : 200
allele2 : ATAAATGTGGATGCTTCACCTGCTAAGAGTTGTCCCACATCGGTGAGGGATAATGTTTACTATGTGCTTATATAATCTTGGGCTACTTCTCATATTGCTAATTGGTTTTATGTTGGAACCCCAATTTCATCATGGTATCAGACAGGATTGTCCTCGTGTGAAGCCAAACGGCCACACGCGCTCCACGTCACCTAGTTGTT : 200
 * 220 * 240 * 260 * 280 * 300 * 320 * 340 * 360 * 380 * 400
allele1 : GTCCACGTGTAGGCTTGAAAATCCACCACACGTGCAGGGGCATGTTGAGAGTCGTCACCTAGTTGTTGTCCACGTGTAGGCTTAAAAATCCACCACACGTGCAGGGGCATGTTGAGAGTTGTCCCACATCGGTGAGGGACAATGTTTGCTATGTGCTTATATGATCTTGGGCTAATTCTCATATTGCCAATTGGTTTTAT : 400
allele2 : GTCCACGTGTAGGCTTGAAAATCCACCACACGTGCAGGGGCATGTTGAGAGTCGTCACCTAGTTGTTGTCCACGTGTAGGCTTAAAAATCCACCACACGTGCAGGGGCATGTTGAGAGTTGTCCCACATCGGTGAGGGACAATGTTTGCTATGTGCTTATATGATCTTGGGCTAATTCTCATATTGCCAATTGGTTTTAT : 400
 * 420 * 440 * 460 * 480 * 500 * 520 * 540 * 560 * 580 * 600
allele1 : GGAGGAACTCCAATTTCATCATCACCTTTATTTTATTAAATTAATGTTTCATTTGGCTATATCTTATTCTTTACGTGACCCTTAAGCCAATTCAAAGCTCTAGAAATAAAGCACAAGATTTACACTTTTTATAATCGAAAATGTATTTTCTCATCATTTCGTGGTGTGTTCAAAATTCAAATAAACACGCAAGGTGTAAG : 600
allele2 : GGAGGAACTCCAATTTCATCATCACCTTTATTTTATTAAATTAATGTTTCATTTGGCTATATCTTATTCTTTACGTGACCCTTAAGCCAATTCAAAGCTCTAGAAATAAAGCACAAGATTTACACTTTTTATAATCGAAAATGTATTTTCTCATCATTTCGTGGTGTGTTCAAAATTCAAATAAACACGCAAGGTGTAAG : 600
 * 620 * 640 * 660 * 680 * 700 * 720 * 740 * 760 * 780 * 800
allele1 : CACATATGCATTTGAAAAGTGGATAACGTACGTTTGCATGGTGACATGCATGAGCCGTCCCTTTGAGATAGCAAAGGGTTATACCATGCCATGATCTAGAAAGAAAACATACACGATAGAAATATATTTGAATTCAAAAAAACTATTTGGACCATTCCGAGCAAGTCTATCTAAATAGCAATAGCATCACTCTATATTAT : 800
allele2 : CACATATGCATTTGAAAAGTGGATAACGTACGTTTGCATGGTGACATGCATGAGCCGTCCCTTTGAGATAGCAAAGGGTTATACCATGCCATGATCTAGAAAGAAAACATACACGATAGAAATATATTTGAATTCAAAAAAACTATTTGGACCATTCCGAGCAAGTCTATCTAAATAGCAATAGCATCACTCTATATTAT : 800
 * 820 * 840 * 860 * 880 * 900 * 920 * 940 * 960 * 980 * 1000
allele1 : TGCAGTTCTTATTAAGAGTGTGTATCAGGCCCGGCCCAGGCGCAGTGCAACAGGGGCAATTGTCCAGGGCCCAAAGTTGAATGGGGCACCCAAAAAACAAAATCCAAATAATTAGGTATAAAAAAAAATTTGTCCAAAGCCCAAAATAAAGGCTGTAGGCGATAACTTGTCCAGGTTTTGGGGATTAAAAATCAATATAA : 1000
allele2 : TGCAGTTCTTATTAAGAGTGTGTATCAGGCCCGGCCCAGGCGCAGTGCAACAGGGGCAATTGTCCAGGGCCCAAAGTTGAATGGGGCACCCAAAAAACAAAATCCAAATAATTAGGTATAAAAAAAAATTTGTCCAAAGCCCAAAATAAAGGCTGTAGGCGATAACTTGTCCAGGTTTTGGGGATTAAAAATCAATATAA : 1000
 * 1020 * 1040 * 1060 * 1080 * 1100 * 1120 * 1140 * 1160 * 1180 * 1200
allele1 : CATAAAGGGTATCTGAAGGGGACAAGTCGTCCAGGTTTTGGGCATGGAGGAGAGGCATAAGATTCAAGTTTTTCAAAGGGAGCTCCCTCTCTGCTTGGAGCTTGTCACCCAACTGCCCAAGCCCAAGGTACTACGGAGAACACAAACCCTTCTCAAATTTCAGCTTAATTTTTGTGTTCTAAGTCGGAAGCTTCGTTTCT : 1200
allele2 : CATAAAGGGTATCTGAAGGGGACAAGTCGTCCAGGTTTTGGGCATGGAGGAGAGGCATAAGATTCAAGTTTTTCAAAGGGAGCTCCCTCTCTGCTTGGAGCTTGTCACCCAACTGCCCAAGCCCAAGGTACTACGGAGAACACAAACCCTTCTCAAATTTCAGCTTAATTTTTGTGTTCTAAGTCGGAAGCTTCGTTTCT : 1200
 * 1220 * 1240 * 1260 * 1280 * 1300 * 1320 * 1340 * 1360 * 1380 * 1400
allele1 : TTTGCTTGAATTGTTCTGGTTTCTGTTTTCAAAATTGGGGTTTTTTTTGTAGCTATTGAGAGGTGCAAGCAGCAGATTTCAGATGCAACCGCAAATTACCTGCATGGACAATCTAAGTGTTCTGAGCAGACTTCAAGTGAGGGGCATGTTTTTGAGGAATTCATTCCATTAACACGGATTTCATACAACTCCAGATGCAC : 1400
allele2 : TTTGCTTGAATTGTTCTGGTTTCTGTTTTCAAAATTGGGGTTTTTTTTGTAGCTATTGAGAGGTGCAAGCAGCAGATTTCAGATGCAACCGCAAATTACCTGCATGGACAATCTAAGTGTTCTGAGCAGACTTCAAGTGAGGGGCATGTTTTTGAGGAATTCATTCCATTAACACGGATTTCATACAACTCCAGATGCAC : 1400
 * 1420 * 1440 * 1460 * 1480 * 1500 * 1520 * 1540 * 1560 * 1580 * 1600
allele1 : CACTCGAAGAGGTATAAAAAAAAGACTCCACCTTCATCAATTTTAGTAAATTTTGTGACTGCCCTTTTGTTTGGTTGCTTATAAAATTTGAGGTGAAGAAGTGGAAAGTATTGATTTTTAGGTGTAGGGTAATGGCAGGAGGAGCAAGTTAAAGGCTATAGTGATGGAGATGAAGAGAAATGGGGTGCTTTTCAGCCTTT : 1600
allele2 : CACTCGAAGAGGTATAAAAAAAAGACTCCACCTTCATCAATTTTAGTAAATTTTGTGACTGCCCTTTTGTTTGGTTGCTTATAAAATTTGAGGTGAAGAAGTGGAAAGTATTGATTTTTAGGTGTAGGGTAATGGCAGGAGGAGCAAGTTAAAGGCTATAGTGATGGAGATGAAGAGAAATGGGGTGCTTTTCAGCCTTT : 1600
 * 1620 * 1640 * 1660 * 1680 * 1700 * 1720 * 1740 * 1760 * 1780 * 1800
allele1 : CCAAGGGAAAAAAAAAGCATTGGAAAGACAAATGGACCGGTGGCTAAAGAACCTTCTTCGGCTCCGGCTACTAGTTCCACCACGGACACCGTGAGTGGAGACAGTGGTGGGAACAACAAGAACAAAGAGAAAGATGGACAGGGAAACGAAGGCGAAACTGGTCACCGGAGTTGCACCGCCGGTTCTTGCATGCTCTTCTC : 1800
allele2 : CCAAGGGAAAAAAAAAGCATTGGAAAGACAAATGGACCGGTGGCTAAAGAACCTTCTTCGGCTCCGGCTACTAGTTCCACCACGGACACCGTGAGTGGAGACAGTGGTGGGAACAACAAGAACAAAGAGAAAGATGGACAGGGAAACGAAGGCGAAACTGGTCACCGGAGTTGCACCGCCGGTTCTTGCATGCTCTTCTC : 1800
 * 1820 * 1840 * 1860 * 1880 * 1900 * 1920 * 1940 * 1960 * 1980 * 2000
allele1 : TTCAACAACTTGGTGGTTCACTTGGTATGCAGTTGAGATTAATTTAGTGGGATATGTTATTTTATATGATTGGATGAATATGTGATTGGATTGTGATGTATTGGATGTGTTTAATGCGTTCTGCGTATATGTGATTTTCCATCTGTTTTTAATCATAATTTGGTTGATGTGAAAATTTTTGCACATACATGATAGAAATT : 2000
allele2 : TTCAACAACTTGGTGGTTCACTTGGTATGCAGTTGAGATTAATTTAGTGGGATATGTTATTTTATATGATTGGATGAATATGTGATTGGATTGTGATGTATTGGATGTGTTTAATGCGTTCTGCGTATATGTGATTTTCCATCTGTTTTTAATCATAATTTGGTTGATGTGAAAATTTTTGCACATACATGATAGAAATT : 2000
 * 2020 * 2040 * 2060 * 2080 * 2100 * 2120 * 2140 * 2160 * 2180 * 2200
allele1 : GGAAGGCATTTTGCATGCTACTAGTGATGATTTTTTTACACATACATGTGATTAACCTCGCAAAATATTTATTTTAAACATATGTTTCTGTACTAAAAAGGGCACATTTTGAACTTTCGCACTGGGCACCAAAAAACTCAGGACCGGCCCTGGTGTGTATAATATTGTACTTGTATCCAAAACAACAATCCTGGCTATTT : 2200
allele2 : GGAAGGCATTTTGCATGCTACTAGTGATGATTTTTTTACACATACATGTGATTAACCTCGCAAAATATTTATTTTAAACATATGTTTCTGTACTAAAAAGGGCACATTTTGAACTTTCGCACTGGGCACCAAAAAACTCAGGACCGGCCCTGGTGTGTATAATATTGTACTTGTATCCAAAACAACAATCCTGGCTATTT : 2200
 * 2220 * 2240 * 2260 * 2280 * 2300 * 2320 * 2340 * 2360 * 2380 * 2400
allele1 : TTACATATTCAATATAAAGTTCCAATACGATCACTGATTGATTAATTGGTATCAAGTACAATACTTCTGCATCTCTCTTAAAAAACATCAACAATGCATTACGTGTTGTAGATTGTTATCAGAGTCGGCCCTGGTCCAGTGTCACCAGGGCGACCGCTCGAGGTCCAAAAAAACGAGGGGCACCAAAAAAAAATTAAGTT : 2400
allele2 : TTACATATTCAATATAAAGTTCCAATACGATCACTGATTGATTAATTGGTATCAAGTACAATACTTCTGCATCTCTCTTAAAAAACATCAACAATGCATTACGTGTTGTAGATTGTTATCAGAGTCGGCCCTGGTCCAGTGTCACCAGGGCGACCGCTCGAGGTCCAAAAAAACGAGGGGCACCAAAAAAAAATTAAGTT : 2400
 * 2420 * 2440 * 2460 * 2480 * 2500 * 2520 * 2540 * 2560 * 2580 * 2600
allele1 : AGTTCATATATGTATAAGGTTTTGTTTTATTCTTGATATATAGTAAACGCTCATATTAAATAGCAATATGTTTTAGATGACAGGGTGGTTTTATTTGGTTTTTCCGTGCCCACCTAGGTTTAATTCCCTCTCTCAACATAAAGGGGTAGTTTCCCTTCTATTTTATTCATGTCTACTTTAAATAACAAACAAACATTAAT : 2600
allele2 : AGTTCATATATGTATAAGGTTTTGTTTTATTCTTGATATATAGTAAACGCTCATATTAAATAGCAATATGTTTTAGATGACAGGGTGGTTTTATTTGGTTTTTCCGTGCCCACCTAGGTTTAATTCCCTCTCTCAACATAAAGGGGTAGTTTCCCTTCTATTTTATTCATGTCTACTTTAAATAACAAACAAACATTAAT : 2600
 * 2620 * 2640 * 2660 * 2680 * 2700 * 2720 * 2740 * 2760 * 2780 * 2800
allele1 : TAGCTTATTAAATGTGGCATTTTCAGTAGTCGTTTTTTGTTTTCAGTTTGAATTAACTCGCAAAATCATGTTCAAGTTTGTCTAAATATAAATTTAGGTTTTTTATATTTGTTGATAATCTTTTCATGGTTAAGTAATAAACTTGTGATCATTATCTTTTTATTGAACGAAGTATTGTTATTGACAATCCAAAAATATCA : 2800
allele2 : TAGCTTATTAAATGTGGCATTTTCAGTAGTCGTTTTTTGTTTTCAGTTTGAATTAACTCGCAAAATCATGTTCAAGTTTGTCTAAATATAAATTTAGGTTTTTTATATTTGTTGATAATCTTTTCATGGTTAAGTAATAAACTTGTGATCATTATCTTTTTATTGAACGAAGTATTGTTATTGACAATCCAAAAATATCA : 2800
 * 2820 * 2840 * 2860 * 2880 * 2900 * 2920 * 2940 * 2960 * 2980 * 3000
allele1 : TCCTACACTTCTTTGTATAATTTTTCCCTTATGATTTTATATATTTGGAATGAACCGCAACTTTTCATGAGCGCTCGAAAAACAAAAACATAGTTGAATTACTATGTGCCCTTTGGTAGTAGCAATTTGTTGTTTTCATTTGTTGCTTACATATAGAAATTAGAGATCTTAAATATGAAAAATTCATTTCAAAAGTTTCA : 3000
allele2 : TCCTACACTTCTTTGTATAATTTTTCCCTTATGATTTTATATATTTGGAATGAACCGCAACTTTTCATGAGCGCTCGAAAAACAAAAACATAGTTGAATTACTATGTGCCCTTTGGTAGTAGCAATTTGTTGTTTTCATTTGTTGCTTACATATAGAAATTAGAGATCTTAAATATGAAAAATTCATTTCAAAAGTTTCA : 3000
 * 3020 * 3040 * 3060 * 3080 * 3100 * 3120 * 3140 * 3160 * 3180 * 3200
allele1 : TAATGTGCAAGTAGCTTGTAGATCAGTTAGTTAAAAGTGTTCAGCTTGTCACTCAATGACTCGTTTTCAAATTTCCTCACCGTATTTCTAATGAGTTTAGTGTAAATTACCCTATCATTTGTCAAAAAAGTCATAATGTGAAAAAGGTCTATTTTCTTTAATATTCAATGTACAACATACGAATACTAAGATAAATAATT : 3200
allele2 : TAATGTGCAAGTAGCTTGTAGATCAGTTAGTTAAAAGTGTTCAGCTTGTCACTCAATGACTCGTTTTCAAATTTCCTCACCGTATTTCTAATGAGTTTAGTGTAAATTACCCTATCATTTGTCAAAAAAGTCATAATGTGAAAAAGGTCTATTTTCTTTAATATTCAATGTACAACATACGAATACTAAGATAAATAATT : 3200
 * 3220 * 3240 * 3260 * 3280 * 3300 * 3320 * 3340 * 3360 * 3380 * 3400
allele1 : ATTTTATGTGAAGTTATGTTAGGGCATATTTTTCAATGTCGTCTAGGGCCTCATAAAACTCAGGATAGGCCTTGATTGTTATATATGGTACTAAACAAAAGTTTCCCAAATACAAAGTTTAAAAAGATTCAAGTGGAATTTTGAAGAATTTTATAAGTATTATAAATTTTGATAAACCCTCGAGTTGATTAATTGCGGGG : 3400
allele2 : ATTTTATGTGAAGTTATGTTAGGGCATATTTTTCAATGTCGTCTAGGGCCTCATAAAACTCAGGATAGGCCTTGATTGTTATATATGGTACTAAACAAAAGTTTCCCAAATACAAAGTTTAAAAAGATTCAAGTGGAATTTTGAAGAATTTTATAAGTATTATAAATTTTGATAAACCCTCGAGTTGATTAATTGCGGGG : 3400
 * 3420 * 3440 * 3460 * 3480 * 3500 * 3520 * 3540 * 3560 * 3580 * 3600
allele1 : GATGTGGTCATCATGACCGCTGTGGTAGTTCATGGAGCAACCTACTTGTTCCCCCACTTATGATTGACGCATTTTTACTGTATGAACATCATAATGCAAAAACTCATTAAATTGAGAGATTTTTTTAGCCTTTCATATGTATCATACAAATAGATGGTTCATCATAAGGATGCTACTTGTTACCAAAAACATTGATTGGT : 3600
allele2 : GATGTGGTCATCATGACCGCTGTGGTAGTTCATGGAGCAACCTACTTGTTCCCCCACTTATGATTGACGCATTTTTACTGTATGAACATCATAATGCAAAAACTCATTAAATTGAGAGATTTTTTTAGCCTTTCATATGTATCATACAAATAGATGGTTCATCATAAGGATGCTACTTGTTACCAAAAACATTGATTGGT : 3600
 * 3620 * 3640 * 3660 * 3680 * 3700 * 3720 * 3740 * 3760 * 3780 * 3800
allele1 : TTGACATATATGGATGGGTAAACAATCTCCAAATGGAATATTTTTTTTAGATATGATATTTCGATGATGATAAAGAGAATGAACGGGTCAAATAATAATGTTCATATGGTGAAGATGCGTAAATCATAAGTGAAGGGACAAGTTGGTTCCCTATGGAGGACACAAGCATTATTCGTTTTTAGGTGTATTCAATTAGAATT : 3800
allele2 : TTGACATATATGGATGGGTAAACAATCTCCAAATGGAATATTTTTTTTAGATATGATATTTCGATGATGATAAAGAGAATGAACGGGTCAAATAATAATGTTCATATGGTGAAGATGCGTAAATCATAAGTGAAGGGACAAGTTGGTTCCCTATGGAGGACACAAGCATTATTCGTTTTTAGGTGTATTCAATTAGAATT : 3800
 * 3820 * 3840 * 3860 * 3880 * 3900 * 3920 * 3940 * 3960 * 3980 * 4000
allele1 : GTAAATGAATCTATAAAAGTTCAGGAATATTCAGTCAGAATGTTAAACAAGCTTATAGAACTCCATACAAATTCAGGTGTATTAATCAATTAAAATTTTAAAGGATTTTATAAAAGTCAACAGAAATCTGAGTGTATTCAAAGAAGATTTTGAAAAAGTCTAAGAAAGTTAGAGTGTATTAATCAGTAATAATTTGATTT : 4000
allele2 : GTAAATGAATCTATAAAAGTTCAGGAATATTCAGTCAGAATGTTAAACAAGCTTATAGAACTCCATACAAATTCAGGTGTATTAATCAATTAAAATTTTAAAGGATTTTATAAAAGTCAACAGAAATCTGAGTGTATTCAAAGAAGATTTTGAAAAAGTCTAAGAAAGTTAGAGTGTATTAATCAGTAATAATTTGATTT : 4000
 * 4020 * 4040 * 4060 * 4080 * 4100 * 4120 * 4140 * 4160 * 4180 * 4200
allele1 : TAAAGAATTTTAAAATGATACATTTTAGTAAATTTGAAGGAATTTCATAGAGTATTTAACCCTTAATAAATCATACTTCTGTAAAGTCCATTAAAAAAACTCCATCAACTTTCATAAATTGAAACATTTTTAAATCCATAAAAGTTGAACCGAATCTATTTTCATATATTATTTTATACATGCAACGACTTACGTTGTAA : 4200
allele2 : TAAAGAATTTTAAAATGATACATTTTAGTAAATTTGAAGGAATTTCATAGAGTATTTAACCCTTAATAAATCATACTTCTGTAAAGTCCATTAAAAAAACTCCATCAACTTTCATAAATTGAAACATTTTTAAATCCATAAAAGTTGAACCGAATCTATTTTCATATATTATTTTATACATGCAACGACTTACGTTGTAA : 4200
 * 4220 * 4240 * 4260 * 4280 * 4300 * 4320 * 4340 * 4360 * 4380 * 4400
allele1 : CATAAGGGATGCAATGCATGGCGCAGAAAGTCATAGAGTCGGCCAAGAGATCATTTCGTCTGAATCTCTCATGTCCGAGAACCCATACCTCAAGAGCCCAAGACGACACAATACACAACAATCGCCGTCAATACCCTTCTCTTCCGCTGCCTATAAATACCAATGGAAATCCCACGACATTCTCACCAAATCATCATCAC : 4400
allele2 : CATAAGGGATGCAATGCATGGCGCAGAAAGTCATAGAGTCGGCCAAGAGATCATTTCGTCTGAATCTCTCATGTCCGAGAACCCATACCTCAAGAGCCCAAGACGACACAATACACAACAATCGCCGTCAATACCCTTCTCTTCCGCTGCCTATAAATACCAATGGAAATCCCACGACATTCTCACCAAATCATCATCAC : 4400
 * 4420 * 4440 * 4460 * 4480 * 4500 * 4520 * 4540 * 4560 * 4580 * 4600
allele1 : TTGAACACACCAATCCATACACTTCTAGCTACAATTCTAAGTTTCCATTTTCCAACATCCCAACACATTGTTCAAAAATATCATCAGCCTCGAGTTAGGGTTTATTATCCTTCGTGACCCTCCTTTTAGTATTTGGTTCTTTTTGAAAGACGATTTGTTAGGTGTTTCTAGGCCTCTAGCCATCTTGTATCTCACCAAAA : 4600
allele2 : TTGAACACACCAATCCATACACTTCTAGCTACAATTCTAAGTTTCCATTTTCCAACATCCCAACACATTGTTCAAAAATATCATCAGCCTCGAGTTAGGGTTTATTATCCTTCGTGACCCTCCTTTTAGTATTTGGTTCTTTTTGAAAGACGATTTGTTAGGTGTTTCTAGGCCTCTAGCCATCTTGTATCTCACCAAAA : 4600
 * 4620 * 4640 *
allele1 : AAAAATATATTACCAGCTGTTTACACCAAATTAAATAGTAAAAAGAAAGCATCAATG : 4657
allele2 : AAAAATATATTACCAGCTGTTTACACCAAATTAAATAGTAAAAAGAAAGCATCAATG : 4657

## Slide 8
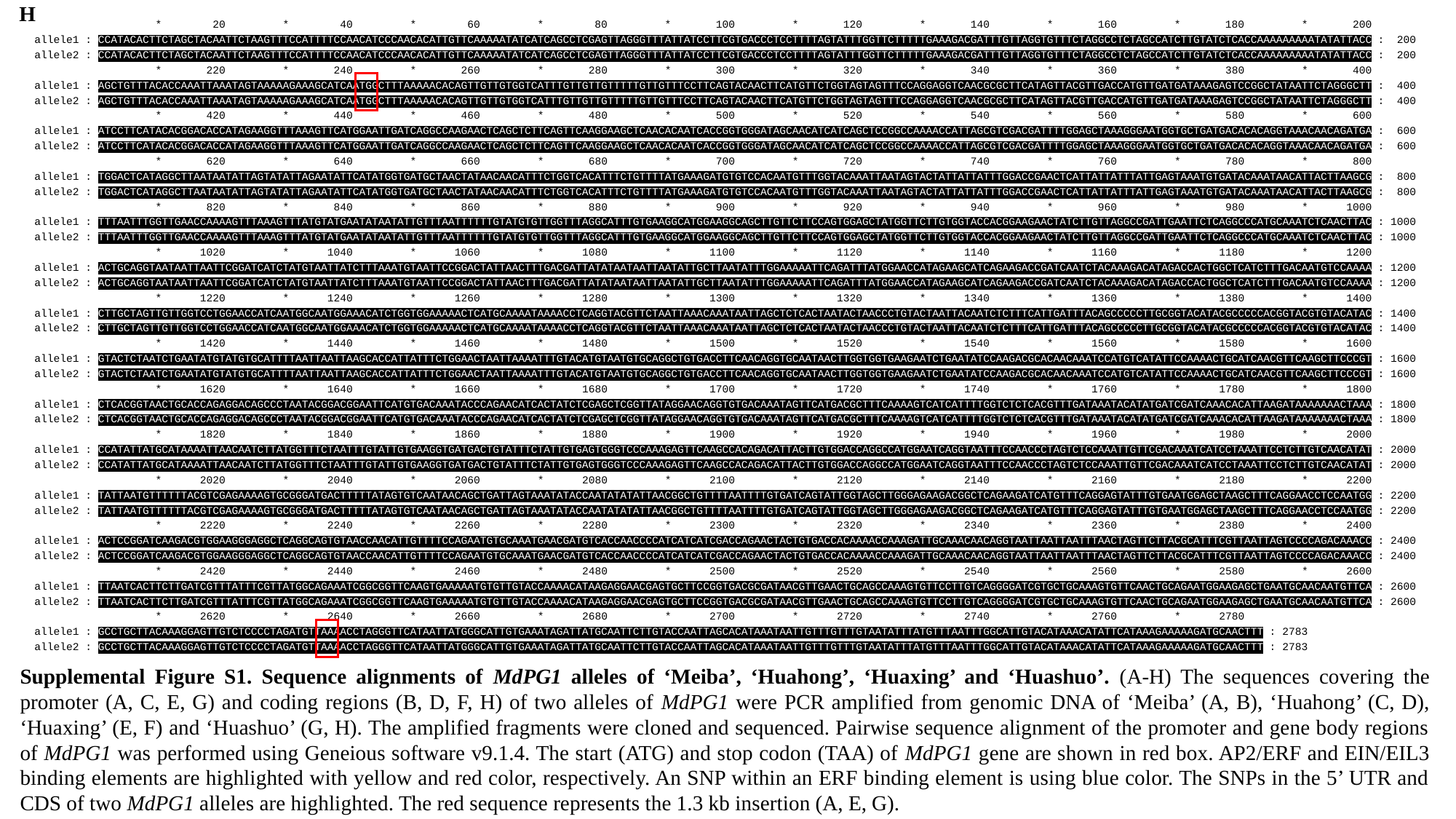

H
 * 20 * 40 * 60 * 80 * 100 * 120 * 140 * 160 * 180 * 200
allele1 : CCATACACTTCTAGCTACAATTCTAAGTTTCCATTTTCCAACATCCCAACACATTGTTCAAAAATATCATCAGCCTCGAGTTAGGGTTTATTATCCTTCGTGACCCTCCTTTTAGTATTTGGTTCTTTTTGAAAGACGATTTGTTAGGTGTTTCTAGGCCTCTAGCCATCTTGTATCTCACCAAAAAAAAATATATTACC : 200
allele2 : CCATACACTTCTAGCTACAATTCTAAGTTTCCATTTTCCAACATCCCAACACATTGTTCAAAAATATCATCAGCCTCGAGTTAGGGTTTATTATCCTTCGTGACCCTCCTTTTAGTATTTGGTTCTTTTTGAAAGACGATTTGTTAGGTGTTTCTAGGCCTCTAGCCATCTTGTATCTCACCAAAAAAAAATATATTACC : 200
 * 220 * 240 * 260 * 280 * 300 * 320 * 340 * 360 * 380 * 400
allele1 : AGCTGTTTACACCAAATTAAATAGTAAAAAGAAAGCATCAATGGCTTTAAAAACACAGTTGTTGTGGTCATTTGTTGTTGTTTTTGTTGTTTCCTTCAGTACAACTTCATGTTCTGGTAGTAGTTTCCAGGAGGTCAACGCGCTTCATAGTTACGTTGACCATGTTGATGATAAAGAGTCCGGCTATAATTCTAGGGCTT : 400
allele2 : AGCTGTTTACACCAAATTAAATAGTAAAAAGAAAGCATCAATGGCTTTAAAAACACAGTTGTTGTGGTCATTTGTTGTTGTTTTTGTTGTTTCCTTCAGTACAACTTCATGTTCTGGTAGTAGTTTCCAGGAGGTCAACGCGCTTCATAGTTACGTTGACCATGTTGATGATAAAGAGTCCGGCTATAATTCTAGGGCTT : 400
 * 420 * 440 * 460 * 480 * 500 * 520 * 540 * 560 * 580 * 600
allele1 : ATCCTTCATACACGGACACCATAGAAGGTTTAAAGTTCATGGAATTGATCAGGCCAAGAACTCAGCTCTTCAGTTCAAGGAAGCTCAACACAATCACCGGTGGGATAGCAACATCATCAGCTCCGGCCAAAACCATTAGCGTCGACGATTTTGGAGCTAAAGGGAATGGTGCTGATGACACACAGGTAAACAACAGATGA : 600
allele2 : ATCCTTCATACACGGACACCATAGAAGGTTTAAAGTTCATGGAATTGATCAGGCCAAGAACTCAGCTCTTCAGTTCAAGGAAGCTCAACACAATCACCGGTGGGATAGCAACATCATCAGCTCCGGCCAAAACCATTAGCGTCGACGATTTTGGAGCTAAAGGGAATGGTGCTGATGACACACAGGTAAACAACAGATGA : 600
 * 620 * 640 * 660 * 680 * 700 * 720 * 740 * 760 * 780 * 800
allele1 : TGGACTCATAGGCTTAATAATATTAGTATATTAGAATATTCATATGGTGATGCTAACTATAACAACATTTCTGGTCACATTTCTGTTTTATGAAAGATGTGTCCACAATGTTTGGTACAAATTAATAGTACTATTATTATTTGGACCGAACTCATTATTATTTATTGAGTAAATGTGATACAAATAACATTACTTAAGCG : 800
allele2 : TGGACTCATAGGCTTAATAATATTAGTATATTAGAATATTCATATGGTGATGCTAACTATAACAACATTTCTGGTCACATTTCTGTTTTATGAAAGATGTGTCCACAATGTTTGGTACAAATTAATAGTACTATTATTATTTGGACCGAACTCATTATTATTTATTGAGTAAATGTGATACAAATAACATTACTTAAGCG : 800
 * 820 * 840 * 860 * 880 * 900 * 920 * 940 * 960 * 980 * 1000
allele1 : TTTAATTTGGTTGAACCAAAAGTTTAAAGTTTATGTATGAATATAATATTGTTTAATTTTTTGTATGTGTTGGTTTAGGCATTTGTGAAGGCATGGAAGGCAGCTTGTTCTTCCAGTGGAGCTATGGTTCTTGTGGTACCACGGAAGAACTATCTTGTTAGGCCGATTGAATTCTCAGGCCCATGCAAATCTCAACTTAC : 1000
allele2 : TTTAATTTGGTTGAACCAAAAGTTTAAAGTTTATGTATGAATATAATATTGTTTAATTTTTTGTATGTGTTGGTTTAGGCATTTGTGAAGGCATGGAAGGCAGCTTGTTCTTCCAGTGGAGCTATGGTTCTTGTGGTACCACGGAAGAACTATCTTGTTAGGCCGATTGAATTCTCAGGCCCATGCAAATCTCAACTTAC : 1000
 * 1020 * 1040 * 1060 * 1080 * 1100 * 1120 * 1140 * 1160 * 1180 * 1200
allele1 : ACTGCAGGTAATAATTAATTCGGATCATCTATGTAATTATCTTTAAATGTAATTCCGGACTATTAACTTTGACGATTATATAATAATTAATATTGCTTAATATTTGGAAAAATTCAGATTTATGGAACCATAGAAGCATCAGAAGACCGATCAATCTACAAAGACATAGACCACTGGCTCATCTTTGACAATGTCCAAAA : 1200
allele2 : ACTGCAGGTAATAATTAATTCGGATCATCTATGTAATTATCTTTAAATGTAATTCCGGACTATTAACTTTGACGATTATATAATAATTAATATTGCTTAATATTTGGAAAAATTCAGATTTATGGAACCATAGAAGCATCAGAAGACCGATCAATCTACAAAGACATAGACCACTGGCTCATCTTTGACAATGTCCAAAA : 1200
 * 1220 * 1240 * 1260 * 1280 * 1300 * 1320 * 1340 * 1360 * 1380 * 1400
allele1 : CTTGCTAGTTGTTGGTCCTGGAACCATCAATGGCAATGGAAACATCTGGTGGAAAAACTCATGCAAAATAAAACCTCAGGTACGTTCTAATTAAACAAATAATTAGCTCTCACTAATACTAACCCTGTACTAATTACAATCTCTTTCATTGATTTACAGCCCCCTTGCGGTACATACGCCCCCACGGTACGTGTACATAC : 1400
allele2 : CTTGCTAGTTGTTGGTCCTGGAACCATCAATGGCAATGGAAACATCTGGTGGAAAAACTCATGCAAAATAAAACCTCAGGTACGTTCTAATTAAACAAATAATTAGCTCTCACTAATACTAACCCTGTACTAATTACAATCTCTTTCATTGATTTACAGCCCCCTTGCGGTACATACGCCCCCACGGTACGTGTACATAC : 1400
 * 1420 * 1440 * 1460 * 1480 * 1500 * 1520 * 1540 * 1560 * 1580 * 1600
allele1 : GTACTCTAATCTGAATATGTATGTGCATTTTAATTAATTAAGCACCATTATTTCTGGAACTAATTAAAATTTGTACATGTAATGTGCAGGCTGTGACCTTCAACAGGTGCAATAACTTGGTGGTGAAGAATCTGAATATCCAAGACGCACAACAAATCCATGTCATATTCCAAAACTGCATCAACGTTCAAGCTTCCCGT : 1600
allele2 : GTACTCTAATCTGAATATGTATGTGCATTTTAATTAATTAAGCACCATTATTTCTGGAACTAATTAAAATTTGTACATGTAATGTGCAGGCTGTGACCTTCAACAGGTGCAATAACTTGGTGGTGAAGAATCTGAATATCCAAGACGCACAACAAATCCATGTCATATTCCAAAACTGCATCAACGTTCAAGCTTCCCGT : 1600
 * 1620 * 1640 * 1660 * 1680 * 1700 * 1720 * 1740 * 1760 * 1780 * 1800
allele1 : CTCACGGTAACTGCACCAGAGGACAGCCCTAATACGGACGGAATTCATGTGACAAATACCCAGAACATCACTATCTCGAGCTCGGTTATAGGAACAGGTGTGACAAATAGTTCATGACGCTTTCAAAAGTCATCATTTTGGTCTCTCACGTTTGATAAATACATATGATCGATCAAACACATTAAGATAAAAAAACTAAA : 1800
allele2 : CTCACGGTAACTGCACCAGAGGACAGCCCTAATACGGACGGAATTCATGTGACAAATACCCAGAACATCACTATCTCGAGCTCGGTTATAGGAACAGGTGTGACAAATAGTTCATGACGCTTTCAAAAGTCATCATTTTGGTCTCTCACGTTTGATAAATACATATGATCGATCAAACACATTAAGATAAAAAAACTAAA : 1800
 * 1820 * 1840 * 1860 * 1880 * 1900 * 1920 * 1940 * 1960 * 1980 * 2000
allele1 : CCATATTATGCATAAAATTAACAATCTTATGGTTTCTAATTTGTATTGTGAAGGTGATGACTGTATTTCTATTGTGAGTGGGTCCCAAAGAGTTCAAGCCACAGACATTACTTGTGGACCAGGCCATGGAATCAGGTAATTTCCAACCCTAGTCTCCAAATTGTTCGACAAATCATCCTAAATTCCTCTTGTCAACATAT : 2000
allele2 : CCATATTATGCATAAAATTAACAATCTTATGGTTTCTAATTTGTATTGTGAAGGTGATGACTGTATTTCTATTGTGAGTGGGTCCCAAAGAGTTCAAGCCACAGACATTACTTGTGGACCAGGCCATGGAATCAGGTAATTTCCAACCCTAGTCTCCAAATTGTTCGACAAATCATCCTAAATTCCTCTTGTCAACATAT : 2000
 * 2020 * 2040 * 2060 * 2080 * 2100 * 2120 * 2140 * 2160 * 2180 * 2200
allele1 : TATTAATGTTTTTTACGTCGAGAAAAGTGCGGGATGACTTTTTATAGTGTCAATAACAGCTGATTAGTAAATATACCAATATATATTAACGGCTGTTTTAATTTTGTGATCAGTATTGGTAGCTTGGGAGAAGACGGCTCAGAAGATCATGTTTCAGGAGTATTTGTGAATGGAGCTAAGCTTTCAGGAACCTCCAATGG : 2200
allele2 : TATTAATGTTTTTTACGTCGAGAAAAGTGCGGGATGACTTTTTATAGTGTCAATAACAGCTGATTAGTAAATATACCAATATATATTAACGGCTGTTTTAATTTTGTGATCAGTATTGGTAGCTTGGGAGAAGACGGCTCAGAAGATCATGTTTCAGGAGTATTTGTGAATGGAGCTAAGCTTTCAGGAACCTCCAATGG : 2200
 * 2220 * 2240 * 2260 * 2280 * 2300 * 2320 * 2340 * 2360 * 2380 * 2400
allele1 : ACTCCGGATCAAGACGTGGAAGGGAGGCTCAGGCAGTGTAACCAACATTGTTTTCCAGAATGTGCAAATGAACGATGTCACCAACCCCATCATCATCGACCAGAACTACTGTGACCACAAAACCAAAGATTGCAAACAACAGGTAATTAATTAATTTAACTAGTTCTTACGCATTTCGTTAATTAGTCCCCAGACAAACC : 2400
allele2 : ACTCCGGATCAAGACGTGGAAGGGAGGCTCAGGCAGTGTAACCAACATTGTTTTCCAGAATGTGCAAATGAACGATGTCACCAACCCCATCATCATCGACCAGAACTACTGTGACCACAAAACCAAAGATTGCAAACAACAGGTAATTAATTAATTTAACTAGTTCTTACGCATTTCGTTAATTAGTCCCCAGACAAACC : 2400
 * 2420 * 2440 * 2460 * 2480 * 2500 * 2520 * 2540 * 2560 * 2580 * 2600
allele1 : TTAATCACTTCTTGATCGTTTATTTCGTTATGGCAGAAATCGGCGGTTCAAGTGAAAAATGTGTTGTACCAAAACATAAGAGGAACGAGTGCTTCCGGTGACGCGATAACGTTGAACTGCAGCCAAAGTGTTCCTTGTCAGGGGATCGTGCTGCAAAGTGTTCAACTGCAGAATGGAAGAGCTGAATGCAACAATGTTCA : 2600
allele2 : TTAATCACTTCTTGATCGTTTATTTCGTTATGGCAGAAATCGGCGGTTCAAGTGAAAAATGTGTTGTACCAAAACATAAGAGGAACGAGTGCTTCCGGTGACGCGATAACGTTGAACTGCAGCCAAAGTGTTCCTTGTCAGGGGATCGTGCTGCAAAGTGTTCAACTGCAGAATGGAAGAGCTGAATGCAACAATGTTCA : 2600
 * 2620 * 2640 * 2660 * 2680 * 2700 * 2720 * 2740 * 2760 * 2780
allele1 : GCCTGCTTACAAAGGAGTTGTCTCCCCTAGATGTTAAAACCTAGGGTTCATAATTATGGGCATTGTGAAATAGATTATGCAATTCTTGTACCAATTAGCACATAAATAATTGTTTGTTTGTAATATTTATGTTTAATTTGGCATTGTACATAAACATATTCATAAAGAAAAAGATGCAACTTT : 2783
allele2 : GCCTGCTTACAAAGGAGTTGTCTCCCCTAGATGTTAAAACCTAGGGTTCATAATTATGGGCATTGTGAAATAGATTATGCAATTCTTGTACCAATTAGCACATAAATAATTGTTTGTTTGTAATATTTATGTTTAATTTGGCATTGTACATAAACATATTCATAAAGAAAAAGATGCAACTTT : 2783
Supplemental Figure S1. Sequence alignments of MdPG1 alleles of ‘Meiba’, ‘Huahong’, ‘Huaxing’ and ‘Huashuo’. (A-H) The sequences covering the promoter (A, C, E, G) and coding regions (B, D, F, H) of two alleles of MdPG1 were PCR amplified from genomic DNA of ‘Meiba’ (A, B), ‘Huahong’ (C, D), ‘Huaxing’ (E, F) and ‘Huashuo’ (G, H). The amplified fragments were cloned and sequenced. Pairwise sequence alignment of the promoter and gene body regions of MdPG1 was performed using Geneious software v9.1.4. The start (ATG) and stop codon (TAA) of MdPG1 gene are shown in red box. AP2/ERF and EIN/EIL3 binding elements are highlighted with yellow and red color, respectively. An SNP within an ERF binding element is using blue color. The SNPs in the 5’ UTR and CDS of two MdPG1 alleles are highlighted. The red sequence represents the 1.3 kb insertion (A, E, G).

## Slide 9
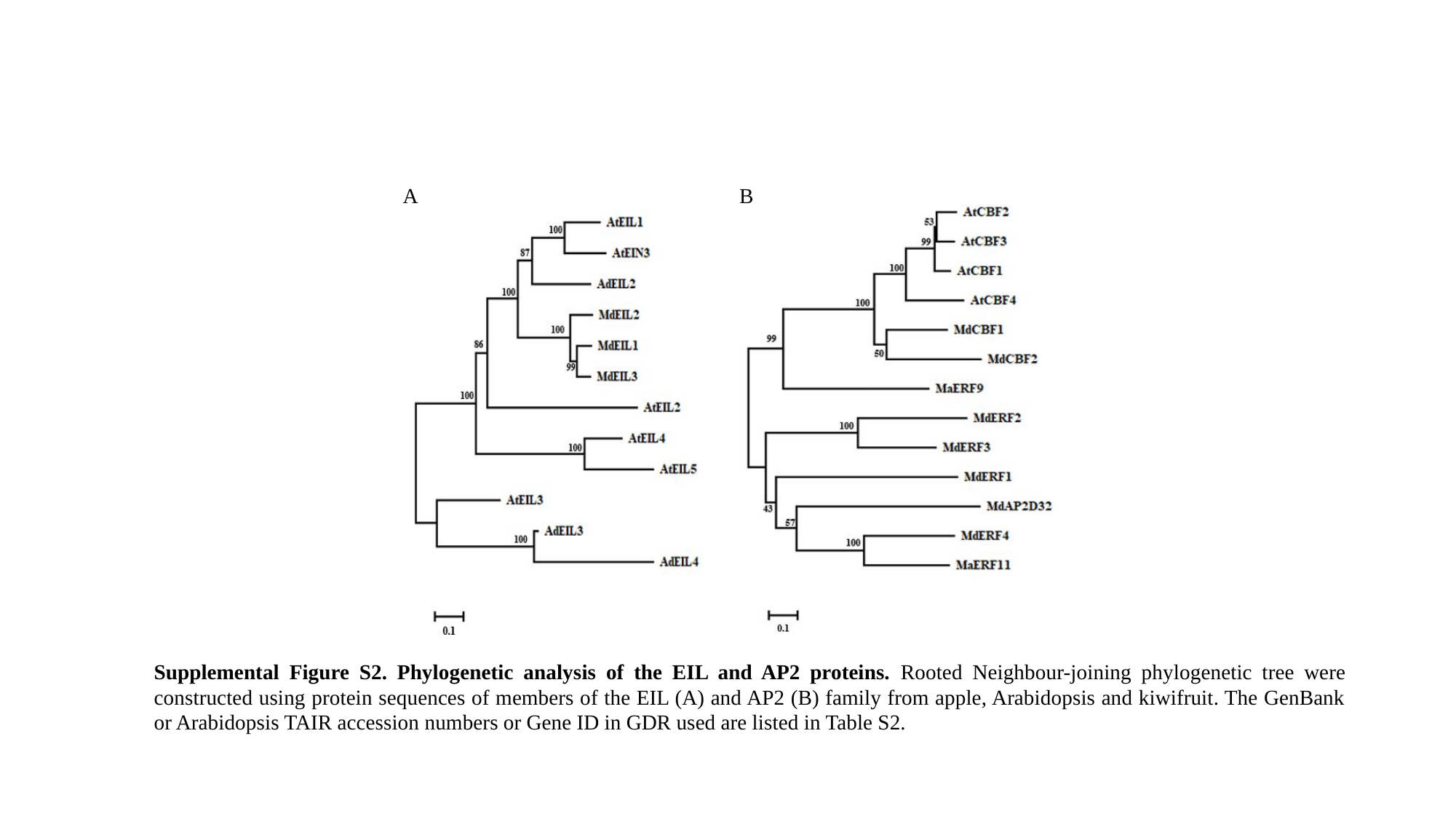

A
B
Supplemental Figure S2. Phylogenetic analysis of the EIL and AP2 proteins. Rooted Neighbour-joining phylogenetic tree were constructed using protein sequences of members of the EIL (A) and AP2 (B) family from apple, Arabidopsis and kiwifruit. The GenBank or Arabidopsis TAIR accession numbers or Gene ID in GDR used are listed in Table S2.

## Slide 10
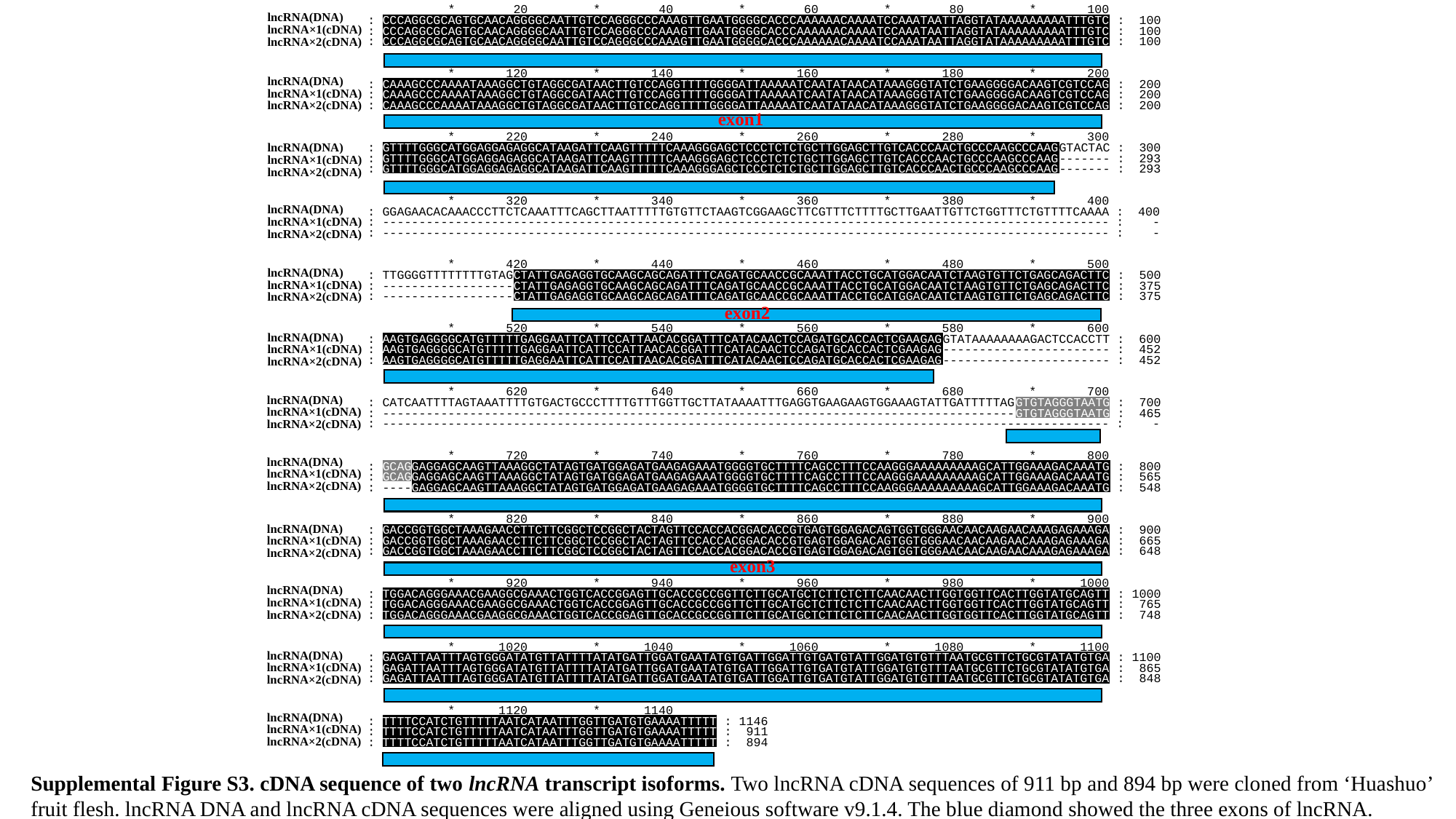

* 20 * 40 * 60 * 80 * 100
 : CCCAGGCGCAGTGCAACAGGGGCAATTGTCCAGGGCCCAAAGTTGAATGGGGCACCCAAAAAACAAAATCCAAATAATTAGGTATAAAAAAAAATTTGTC : 100
 : CCCAGGCGCAGTGCAACAGGGGCAATTGTCCAGGGCCCAAAGTTGAATGGGGCACCCAAAAAACAAAATCCAAATAATTAGGTATAAAAAAAAATTTGTC : 100
 : CCCAGGCGCAGTGCAACAGGGGCAATTGTCCAGGGCCCAAAGTTGAATGGGGCACCCAAAAAACAAAATCCAAATAATTAGGTATAAAAAAAAATTTGTC : 100
 * 120 * 140 * 160 * 180 * 200
 : CAAAGCCCAAAATAAAGGCTGTAGGCGATAACTTGTCCAGGTTTTGGGGATTAAAAATCAATATAACATAAAGGGTATCTGAAGGGGACAAGTCGTCCAG : 200
 : CAAAGCCCAAAATAAAGGCTGTAGGCGATAACTTGTCCAGGTTTTGGGGATTAAAAATCAATATAACATAAAGGGTATCTGAAGGGGACAAGTCGTCCAG : 200
 : CAAAGCCCAAAATAAAGGCTGTAGGCGATAACTTGTCCAGGTTTTGGGGATTAAAAATCAATATAACATAAAGGGTATCTGAAGGGGACAAGTCGTCCAG : 200
 * 220 * 240 * 260 * 280 * 300
 : GTTTTGGGCATGGAGGAGAGGCATAAGATTCAAGTTTTTCAAAGGGAGCTCCCTCTCTGCTTGGAGCTTGTCACCCAACTGCCCAAGCCCAAGGTACTAC : 300
 : GTTTTGGGCATGGAGGAGAGGCATAAGATTCAAGTTTTTCAAAGGGAGCTCCCTCTCTGCTTGGAGCTTGTCACCCAACTGCCCAAGCCCAAG------- : 293
 : GTTTTGGGCATGGAGGAGAGGCATAAGATTCAAGTTTTTCAAAGGGAGCTCCCTCTCTGCTTGGAGCTTGTCACCCAACTGCCCAAGCCCAAG------- : 293
 * 320 * 340 * 360 * 380 * 400
 : GGAGAACACAAACCCTTCTCAAATTTCAGCTTAATTTTTGTGTTCTAAGTCGGAAGCTTCGTTTCTTTTGCTTGAATTGTTCTGGTTTCTGTTTTCAAAA : 400
 : ---------------------------------------------------------------------------------------------------- : -
 : ---------------------------------------------------------------------------------------------------- : -
 * 420 * 440 * 460 * 480 * 500
 : TTGGGGTTTTTTTTGTAGCTATTGAGAGGTGCAAGCAGCAGATTTCAGATGCAACCGCAAATTACCTGCATGGACAATCTAAGTGTTCTGAGCAGACTTC : 500
 : ------------------CTATTGAGAGGTGCAAGCAGCAGATTTCAGATGCAACCGCAAATTACCTGCATGGACAATCTAAGTGTTCTGAGCAGACTTC : 375
 : ------------------CTATTGAGAGGTGCAAGCAGCAGATTTCAGATGCAACCGCAAATTACCTGCATGGACAATCTAAGTGTTCTGAGCAGACTTC : 375
 * 520 * 540 * 560 * 580 * 600
 : AAGTGAGGGGCATGTTTTTGAGGAATTCATTCCATTAACACGGATTTCATACAACTCCAGATGCACCACTCGAAGAGGTATAAAAAAAAGACTCCACCTT : 600
 : AAGTGAGGGGCATGTTTTTGAGGAATTCATTCCATTAACACGGATTTCATACAACTCCAGATGCACCACTCGAAGAG----------------------- : 452
 : AAGTGAGGGGCATGTTTTTGAGGAATTCATTCCATTAACACGGATTTCATACAACTCCAGATGCACCACTCGAAGAG----------------------- : 452
 * 620 * 640 * 660 * 680 * 700
 : CATCAATTTTAGTAAATTTTGTGACTGCCCTTTTGTTTGGTTGCTTATAAAATTTGAGGTGAAGAAGTGGAAAGTATTGATTTTTAGGTGTAGGGTAATG : 700
 : ---------------------------------------------------------------------------------------GTGTAGGGTAATG : 465
 : ---------------------------------------------------------------------------------------------------- : -
 * 720 * 740 * 760 * 780 * 800
 : GCAGGAGGAGCAAGTTAAAGGCTATAGTGATGGAGATGAAGAGAAATGGGGTGCTTTTCAGCCTTTCCAAGGGAAAAAAAAAGCATTGGAAAGACAAATG : 800
 : GCAGGAGGAGCAAGTTAAAGGCTATAGTGATGGAGATGAAGAGAAATGGGGTGCTTTTCAGCCTTTCCAAGGGAAAAAAAAAGCATTGGAAAGACAAATG : 565
 : ----GAGGAGCAAGTTAAAGGCTATAGTGATGGAGATGAAGAGAAATGGGGTGCTTTTCAGCCTTTCCAAGGGAAAAAAAAAGCATTGGAAAGACAAATG : 548
 * 820 * 840 * 860 * 880 * 900
 : GACCGGTGGCTAAAGAACCTTCTTCGGCTCCGGCTACTAGTTCCACCACGGACACCGTGAGTGGAGACAGTGGTGGGAACAACAAGAACAAAGAGAAAGA : 900
 : GACCGGTGGCTAAAGAACCTTCTTCGGCTCCGGCTACTAGTTCCACCACGGACACCGTGAGTGGAGACAGTGGTGGGAACAACAAGAACAAAGAGAAAGA : 665
 : GACCGGTGGCTAAAGAACCTTCTTCGGCTCCGGCTACTAGTTCCACCACGGACACCGTGAGTGGAGACAGTGGTGGGAACAACAAGAACAAAGAGAAAGA : 648
 * 920 * 940 * 960 * 980 * 1000
 : TGGACAGGGAAACGAAGGCGAAACTGGTCACCGGAGTTGCACCGCCGGTTCTTGCATGCTCTTCTCTTCAACAACTTGGTGGTTCACTTGGTATGCAGTT : 1000
 : TGGACAGGGAAACGAAGGCGAAACTGGTCACCGGAGTTGCACCGCCGGTTCTTGCATGCTCTTCTCTTCAACAACTTGGTGGTTCACTTGGTATGCAGTT : 765
 : TGGACAGGGAAACGAAGGCGAAACTGGTCACCGGAGTTGCACCGCCGGTTCTTGCATGCTCTTCTCTTCAACAACTTGGTGGTTCACTTGGTATGCAGTT : 748
 * 1020 * 1040 * 1060 * 1080 * 1100
 : GAGATTAATTTAGTGGGATATGTTATTTTATATGATTGGATGAATATGTGATTGGATTGTGATGTATTGGATGTGTTTAATGCGTTCTGCGTATATGTGA : 1100
 : GAGATTAATTTAGTGGGATATGTTATTTTATATGATTGGATGAATATGTGATTGGATTGTGATGTATTGGATGTGTTTAATGCGTTCTGCGTATATGTGA : 865
 : GAGATTAATTTAGTGGGATATGTTATTTTATATGATTGGATGAATATGTGATTGGATTGTGATGTATTGGATGTGTTTAATGCGTTCTGCGTATATGTGA : 848
 * 1120 * 1140
 : TTTTCCATCTGTTTTTAATCATAATTTGGTTGATGTGAAAATTTTT : 1146
 : TTTTCCATCTGTTTTTAATCATAATTTGGTTGATGTGAAAATTTTT : 911
 : TTTTCCATCTGTTTTTAATCATAATTTGGTTGATGTGAAAATTTTT : 894
lncRNA(DNA)
lncRNA×1(cDNA)
lncRNA×2(cDNA)
lncRNA(DNA)
lncRNA×1(cDNA)
lncRNA×2(cDNA)
lncRNA(DNA)
lncRNA×1(cDNA)
lncRNA×2(cDNA)
lncRNA(DNA)
lncRNA×1(cDNA)
lncRNA×2(cDNA)
lncRNA(DNA)
lncRNA×1(cDNA)
lncRNA×2(cDNA)
lncRNA(DNA)
lncRNA×1(cDNA)
lncRNA×2(cDNA)
lncRNA(DNA)
lncRNA×1(cDNA)
lncRNA×2(cDNA)
lncRNA(DNA)
lncRNA×1(cDNA)
lncRNA×2(cDNA)
exon1
exon2
lncRNA(DNA)
lncRNA×1(cDNA)
lncRNA×2(cDNA)
lncRNA(DNA)
lncRNA×1(cDNA)
lncRNA×2(cDNA)
exon3
lncRNA(DNA)
lncRNA×1(cDNA)
lncRNA×2(cDNA)
lncRNA(DNA)
lncRNA×1(cDNA)
lncRNA×2(cDNA)
Supplemental Figure S3. cDNA sequence of two lncRNA transcript isoforms. Two lncRNA cDNA sequences of 911 bp and 894 bp were cloned from ‘Huashuo’ fruit flesh. lncRNA DNA and lncRNA cDNA sequences were aligned using Geneious software v9.1.4. The blue diamond showed the three exons of lncRNA.

## Slide 11
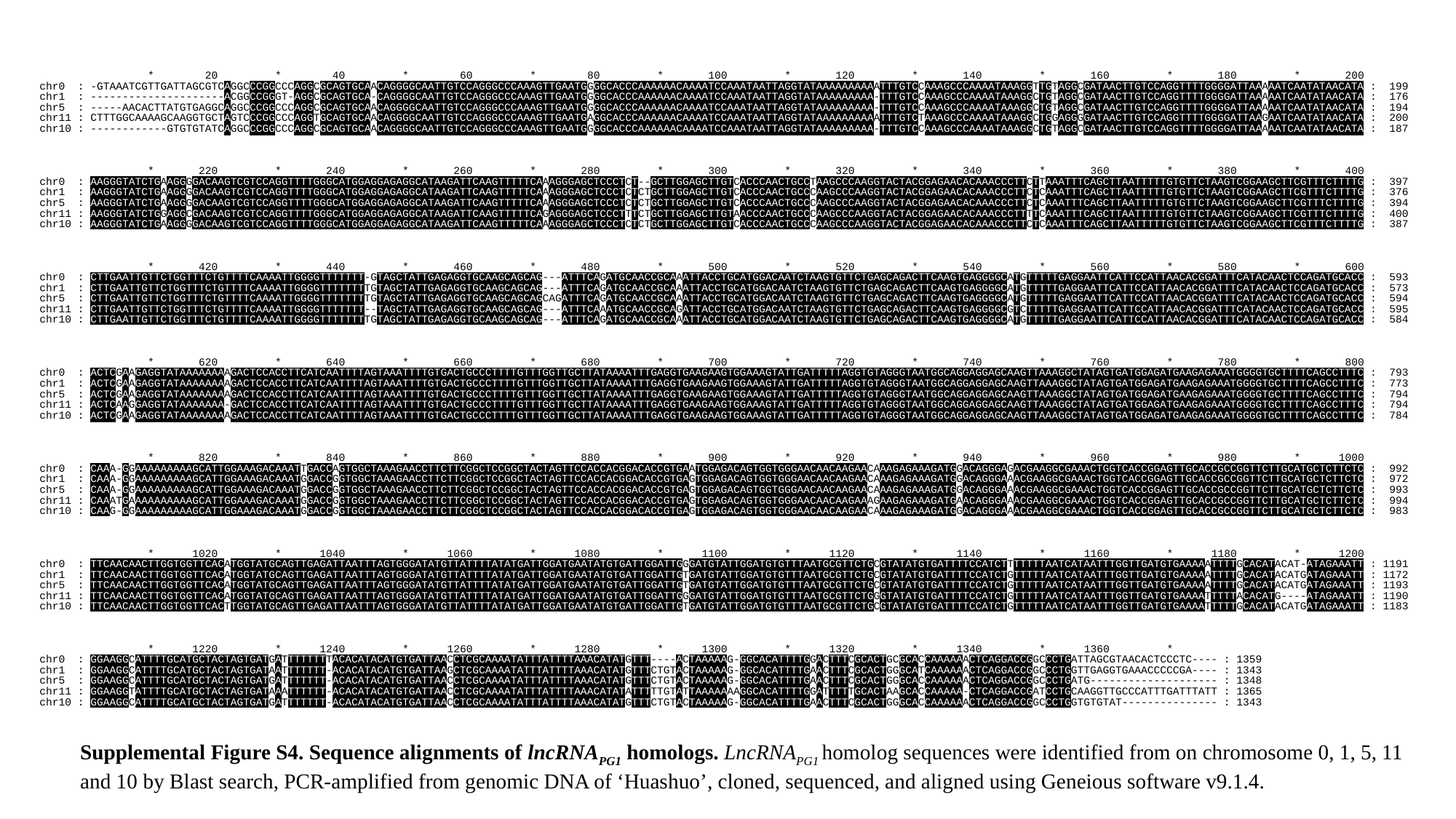

* 20 * 40 * 60 * 80 * 100 * 120 * 140 * 160 * 180 * 200
chr0 : -GTAAATCGTTGATTAGCGTCAGGCCCGGCCCAGGCGCAGTGCAACAGGGGCAATTGTCCAGGGCCCAAAGTTGAATGGGGCACCCAAAAAACAAAATCCAAATAATTAGGTATAAAAAAAAAATTTGTCCAAAGCCCAAAATAAAGGTTGTAGGCGATAACTTGTCCAGGTTTTGGGGATTAAAAATCAATATAACATA : 199
chr1 : ---------------------ACGGCCGGGT-AGGCGCAGTGCA-CAGGGGCAATTGTCCAGGGCCCAAAGTTGAATGGGGCACCCAAAAAACAAAATCCAAATAATTAGGTATAAAAAAAAA-TTTGTCCAAAGCCCAAAATAAAGGCTGTAGGCGATAACTTGTCCAGGTTTTGGGGATTAAAAATCAATATAACATA : 176
chr5 : -----AACACTTATGTGAGGCAGGCCCGGCCCAGGCGCAGTGCAACAGGGGCAATTGTCCAGGGCCCAAAGTTGAATGGGGCACCCAAAAAACAAAATCCAAATAATTAGGTATAAAAAAAAA-TTTGTCCAAAGCCCAAAATAAAGGCTGTAGGCGATAACTTGTCCAGGTTTTGGGGATTAAAAATCAATATAACATA : 194
chr11 : CTTTGGCAAAAGCAAGGTGCTAGTCCCGGCCCAGGTGCAGTGCAACAGGGGCAATTGTCCAGGGCCCAAAGTTGAATGAGGCACCCAAAAAACAAAATCCAAATAATTAGGTATAAAAAAAAAATTTGTCTAAAGCCCAAAATAAAGGCTGGAGGGGATAACTTGTCCAGGTTTTGGGGATTAAGAATCAATATAACATA : 200
chr10 : ------------GTGTGTATCAGGCCCGGCCCAGGCGCAGTGCAACAGGGGCAATTGTCCAGGGCCCAAAGTTGAATGGGGCACCCAAAAAACAAAATCCAAATAATTAGGTATAAAAAAAAA-TTTGTCCAAAGCCCAAAATAAAGGCTGTAGGCGATAACTTGTCCAGGTTTTGGGGATTAAAAATCAATATAACATA : 187
 * 220 * 240 * 260 * 280 * 300 * 320 * 340 * 360 * 380 * 400
chr0 : AAGGGTATCTGAAGGGGACAAGTCGTCCAGGTTTTGGGCATGGAGGAGAGGCATAAGATTCAAGTTTTTCAAAGGGAGCTCCCTCT--GCTTGGAGCTTGTCACCCAACTGCCTAAGCCCAAGGTACTACGGAGAACACAAACCCTTCTTAAATTTCAGCTTAATTTTTGTGTTCTAAGTCGGAAGCTTCGTTTCTTTTG : 397
chr1 : AAGGGTATCTGAAGGGGACAAGTCGTCCAGGTTTTGGGCATGGAGGAGAGGCATAAGATTCAAGTTTTTCAAAGGGAGCTCCCTCTCTGCTTGGAGCTTGTCACCCAACTGCCCAAGCCCAAGGTACTACGGAGAACACAAACCCTTCTCAAATTTCAGCTTAATTTTTGTGTTCTAAGTCGGAAGCTTCGTTTCTTTTG : 376
chr5 : AAGGGTATCTGAAGGGGACAAGTCGTCCAGGTTTTGGGCATGGAGGAGAGGCATAAGATTCAAGTTTTTCAAAGGGAGCTCCCTCTCTGCTTGGAGCTTGTCACCCAACTGCCCAAGCCCAAGGTACTACGGAGAACACAAACCCTTCTCAAATTTCAGCTTAATTTTTGTGTTCTAAGTCGGAAGCTTCGTTTCTTTTG : 394
chr11 : AAGGGTATCTGGAGGCGACAAGTCGTCCAGGTTTTGGGCATGGAGGAGAGGCATAAGATTCAAGTTTTTCAGAGGGAGCTCCCTTTCTGCTTGGAGCTTGTAACCCAACTGCCCAAGCCCAAGGTACTACGGAGAACACAAACCCTTTTCAAATTTCAGCTTAATTTTTGTGTTCTAAGTCGGAAGCTTCGTTTCTTTTG : 400
chr10 : AAGGGTATCTGAAGGGGACAAGTCGTCCAGGTTTTGGGCATGGAGGAGAGGCATAAGATTCAAGTTTTTCAAAGGGAGCTCCCTCTCTGCTTGGAGCTTGTCACCCAACTGCCCAAGCCCAAGGTACTACGGAGAACACAAACCCTTCTCAAATTTCAGCTTAATTTTTGTGTTCTAAGTCGGAAGCTTCGTTTCTTTTG : 387
 * 420 * 440 * 460 * 480 * 500 * 520 * 540 * 560 * 580 * 600
chr0 : CTTGAATTGTTCTGGTTTCTGTTTTCAAAATTGGGGTTTTTTT-GTAGCTATTGAGAGGTGCAAGCAGCAG---ATTTCAGATGCAACCGCAAATTACCTGCATGGACAATCTAAGTGTTCTGAGCAGACTTCAAGTGAGGGGCATGTTTTTGAGGAATTCATTCCATTAACACGGATTTCATACAACTCCAGATGCACC : 593
chr1 : CTTGAATTGTTCTGGTTTCTGTTTTCAAAATTGGGGTTTTTTTTGTAGCTATTGAGAGGTGCAAGCAGCAG---ATTTCAGATGCAACCGCAAATTACCTGCATGGACAATCTAAGTGTTCTGAGCAGACTTCAAGTGAGGGGCATGTTTTTGAGGAATTCATTCCATTAACACGGATTTCATACAACTCCAGATGCACC : 573
chr5 : CTTGAATTGTTCTGGTTTCTGTTTTCAAAATTGGGGTTTTTTTTGTAGCTATTGAGAGGTGCAAGCAGCAGCAGATTTCAGATGCAACCGCAAATTACCTGCATGGACAATCTAAGTGTTCTGAGCAGACTTCAAGTGAGGGGCATGTTTTTGAGGAATTCATTCCATTAACACGGATTTCATACAACTCCAGATGCACC : 594
chr11 : CTTGAATTGTTCTGGTTTCTGTTTTCAAAATTGGGGTTTTTTT--TAGCTATTGAGAGGTGCAAGCAGCAG---ATTTCAAATGCAACCGCAGATTACCTGCATGGACAATCTAAGTGTTCTGAGCAGACTTCAAGTGAGGGGCGTCTTTTTGAGGAATTCATTCCATTAACACGGATTTCATACAACTCCAGATGCACC : 595
chr10 : CTTGAATTGTTCTGGTTTCTGTTTTCAAAATTGGGGTTTTTTTTGTAGCTATTGAGAGGTGCAAGCAGCAG---ATTTCAGATGCAACCGCAAATTACCTGCATGGACAATCTAAGTGTTCTGAGCAGACTTCAAGTGAGGGGCATGTTTTTGAGGAATTCATTCCATTAACACGGATTTCATACAACTCCAGATGCACC : 584
 * 620 * 640 * 660 * 680 * 700 * 720 * 740 * 760 * 780 * 800
chr0 : ACTCGAAGAGGTATAAAAAAAAGACTCCACCTTCATCAATTTTAGTAAATTTTGTGACTGCCCTTTTGTTTGGTTGCTTATAAAATTTGAGGTGAAGAAGTGGAAAGTATTGATTTTTAGGTGTAGGGTAATGGCAGGAGGAGCAAGTTAAAGGCTATAGTGATGGAGATGAAGAGAAATGGGGTGCTTTTCAGCCTTTC : 793
chr1 : ACTCGAAGAGGTATAAAAAAAAGACTCCACCTTCATCAATTTTAGTAAATTTTGTGACTGCCCTTTTGTTTGGTTGCTTATAAAATTTGAGGTGAAGAAGTGGAAAGTATTGATTTTTAGGTGTAGGGTAATGGCAGGAGGAGCAAGTTAAAGGCTATAGTGATGGAGATGAAGAGAAATGGGGTGCTTTTCAGCCTTTC : 773
chr5 : ACTCGAAGAGGTATAAAAAAAAGACTCCACCTTCATCAATTTTAGTAAATTTTGTGACTGCCCTTTTGTTTGGTTGCTTATAAAATTTGAGGTGAAGAAGTGGAAAGTATTGATTTTTAGGTGTAGGGTAATGGCAGGAGGAGCAAGTTAAAGGCTATAGTGATGGAGATGAAGAGAAATGGGGTGCTTTTCAGCCTTTC : 794
chr11 : ACTCAAGGAGGTATAAAAAAA-GACTCCACCTTCATCAATTTTAGTAAATTTTGTGACTGCCCTTTTGTTTGGTTGCTTATAAAATTTGAGGTGAAGAAGTGGAAAGTATTGATTTTTAGGTGTAGGGTAATGGCAGGAGGAGCAAGTTAAAGGCTATAGTGATGGAGATGAAGAGAAATGGGGTGCTTTTCAGCCTTTC : 794
chr10 : ACTCGAAGAGGTATAAAAAAAAGACTCCACCTTCATCAATTTTAGTAAATTTTGTGACTGCCCTTTTGTTTGGTTGCTTATAAAATTTGAGGTGAAGAAGTGGAAAGTATTGATTTTTAGGTGTAGGGTAATGGCAGGAGGAGCAAGTTAAAGGCTATAGTGATGGAGATGAAGAGAAATGGGGTGCTTTTCAGCCTTTC : 784
 * 820 * 840 * 860 * 880 * 900 * 920 * 940 * 960 * 980 * 1000
chr0 : CAAA-GGAAAAAAAAAGCATTGGAAAGACAAATTGACCAGTGGCTAAAGAACCTTCTTCGGCTCCGGCTACTAGTTCCACCACGGACACCGTGAATGGAGACAGTGGTGGGAACAACAAGAACAAAGAGAAAGATGGACAGGGAGACGAAGGCGAAACTGGTCACCGGAGTTGCACCGCCGGTTCTTGCATGCTCTTCTC : 992
chr1 : CAAA-GGAAAAAAAAAGCATTGGAAAGACAAATGGACCGGTGGCTAAAGAACCTTCTTCGGCTCCGGCTACTAGTTCCACCACGGACACCGTGAGTGGAGACAGTGGTGGGAACAACAAGAACAAAGAGAAAGATGGACAGGGAAACGAAGGCGAAACTGGTCACCGGAGTTGCACCGCCGGTTCTTGCATGCTCTTCTC : 972
chr5 : CAAA-GGAAAAAAAAAGCATTGGAAAGACAAATGGACCGGTGGCTAAAGAACCTTCTTCGGCTCCGGCTACTAGTTCCACCACGGACACCGTGAGTGGAGACAGTGGTGGGAACAACAAGAACAAAGAGAAAGATGGACAGGGAAACGAAGGCGAAACTGGTCACCGGAGTTGCACCGCCGGTTCTTGCATGCTCTTCTC : 993
chr11 : CAAATGAAAAAAAAAAGCATTGGAAAGACAAATGGACCGGTGGCTAAAGAACCTTCTTCGGCTCCGGCTACTAGTTCCACCACGGACACCGTGAGTGGAGACAGTGGTGGGAACAACAAGAAAGAAGAGAAAGATGAACAGGGAAACGAAGGCGAAACTGGTCACCGGAGTTGCACCGCCGGTTCTTGCATGCTCTTCTC : 994
chr10 : CAAG-GGAAAAAAAAAGCATTGGAAAGACAAATGGACCGGTGGCTAAAGAACCTTCTTCGGCTCCGGCTACTAGTTCCACCACGGACACCGTGAGTGGAGACAGTGGTGGGAACAACAAGAACAAAGAGAAAGATGGACAGGGAAACGAAGGCGAAACTGGTCACCGGAGTTGCACCGCCGGTTCTTGCATGCTCTTCTC : 983
 * 1020 * 1040 * 1060 * 1080 * 1100 * 1120 * 1140 * 1160 * 1180 * 1200
chr0 : TTCAACAACTTGGTGGTTCACATGGTATGCAGTTGAGATTAATTTAGTGGGATATGTTATTTTATATGATTGGATGAATATGTGATTGGATTGGGATGTATTGGATGTGTTTAATGCGTTCTGCGTATATGTGATTTTCCATCTTTTTTTAATCATAATTTGGTTGATGTGAAAAATTTTGCACATACAT-ATAGAAATT : 1191
chr1 : TTCAACAACTTGGTGGTTCACATGGTATGCAGTTGAGATTAATTTAGTGGGATATGTTATTTTATATGATTGGATGAATATGTGATTGGATTGTGATGTATTGGATGTGTTTAATGCGTTCTGCGTATATGTGATTTTCCATCTGTTTTTAATCATAATTTGGTTGATGTGAAAAATTTTGCACATACATGATAGAAATT : 1172
chr5 : TTCAACAACTTGGTGGTTCACATGGTATGCAGTTGAGATTAATTTAGTGGGATATGTTATTTTATATGATTGGATGAATATGTGATTGGATTGTGATGTATTGGATGTGTTTAATGCGTTCTGCGTATATGTGATTTTCCATCTGTTTTTAATCATAATTTGGTTGATGTGAAAAATTTTGCACATACATGATAGAAATT : 1193
chr11 : TTCAACAACTTGGTGGTTCACATGGTATGCAGTTGAGATTAATTTAGTGGGATATGTTATTTTATATGATTGGATGAATATGTGATTGGATTGGGATGTATTGGATGTGTTTAATGCGTTCTGGGTATATGTGATTTTCCATCTGTTTTTAATCATAATTTGGTTGATGTGAAAATTTTTACACATG----ATAGAAATT : 1190
chr10 : TTCAACAACTTGGTGGTTCACTTGGTATGCAGTTGAGATTAATTTAGTGGGATATGTTATTTTATATGATTGGATGAATATGTGATTGGATTGTGATGTATTGGATGTGTTTAATGCGTTCTGCGTATATGTGATTTTCCATCTGTTTTTAATCATAATTTGGTTGATGTGAAAATTTTTGCACATACATGATAGAAATT : 1183
 * 1220 * 1240 * 1260 * 1280 * 1300 * 1320 * 1340 * 1360 *
chr0 : GGAAGGCATTTTGCATGCTACTAGTGATGATTTTTTTTACACATACATGTGATTAACCTCGCAAAATATTTATTTTAAACATATGTTT----ACTAAAAAG-GGCACATTTTGGACTTTCGCACTGCGCACCAAAAAACTCAGGACCGGCCCTGATTAGCGTAACACTCCCTC---- : 1359
chr1 : GGAAGGCATTTTGCATGCTACTAGTGATAATTTTTTT-ACACATACATGTGATTAAGCTCGCAAAATATTTATTTTAAACATATGTTTCTGTACTAAAAAG-GGCACATTTTGAACTTTCGCACTGGGCATCAAAAAACTCAGGACCGGCCCTGGTTGAGGTGAAACCCCCGA---- : 1343
chr5 : GGAAGGCATTTTGCATGCTACTAGTGATGATTTTTTT-ACACATACATGTGATTAACCTCGCAAAATATTTATTTTAAACATATGTTTCTGTACTAAAAAG-GGCACATTTTGAACTTTCGCACTGGGCACCAAAAAACTCAGGACCGGCCCTGATG-------------------- : 1348
chr11 : GGAAGGTATTTTGCATGCTACTAGTGATAAATTTTTT-ACACATACATGTGATTAACCTCGCAAAATATTTATTTTAAACATATATTTTTGTATTAAAAAAAGGCACATTTTGGATTTTTGCACTAAGCACCAAAAA-CTCAGGACCGATCCTGCAAGGTTGCCCATTTGATTTATT : 1365
chr10 : GGAAGGCATTTTGCATGCTACTAGTGATGATTTTTTT-ACACATACATGTGATTAACCTCGCAAAATATTTATTTTAAACATATGTTTCTGTACTAAAAAG-GGCACATTTTGAACTTTCGCACTGGGCACCAAAAAACTCAGGACCGGCCCTGGTGTGTAT--------------- : 1343
Supplemental Figure S4. Sequence alignments of lncRNAPG1 homologs. LncRNAPG1 homolog sequences were identified from on chromosome 0, 1, 5, 11 and 10 by Blast search, PCR-amplified from genomic DNA of ‘Huashuo’, cloned, sequenced, and aligned using Geneious software v9.1.4.

## Slide 12
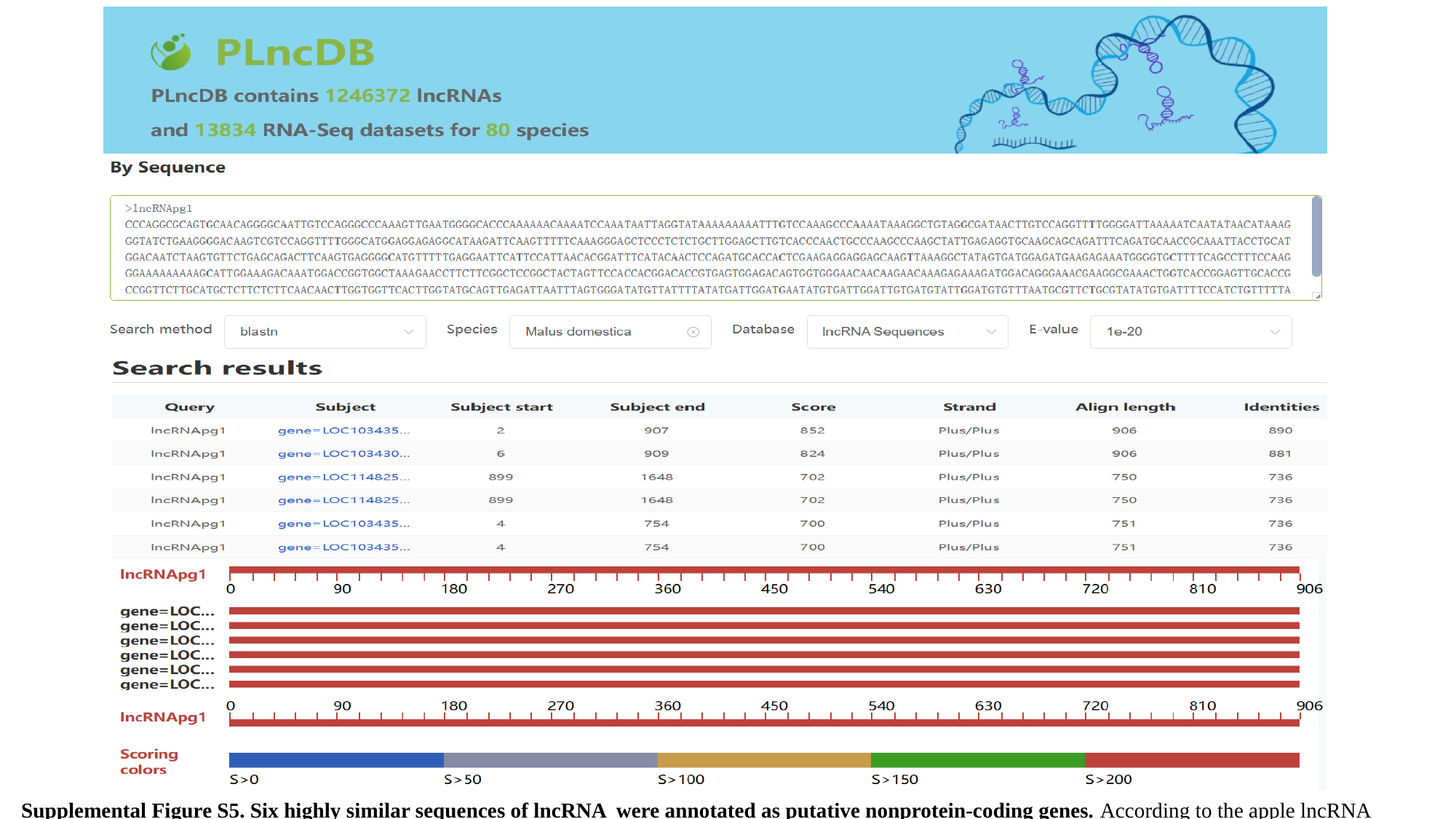

Supplemental Figure S5. Six highly similar sequences of lncRNA were annotated as putative nonprotein-coding genes. According to the apple lncRNA database information, six highly similar sequences of lncRNA were annotated as putative nonprotein-coding genes. PLnc DB (https://www.tobaccodb.org/plncdb/)
